# Direct and Indirect Pathways of CdTeSe Magic-Size Cluster Isomerization Induced by Surface Ligands at Room Temperature

**DOI:** 10.1021/acscentsci.2c01394

**Published:** 2023-03-08

**Authors:** Yusha Yang, Qiu Shen, Chunchun Zhang, Nelson Rowell, Meng Zhang, Xiaoqin Chen, Chaoran Luan, Kui Yu

**Affiliations:** † Engineering Research Center in Biomaterials, Sichuan University, Chengdu, Sichuan 610065, P. R. China; ‡ Analytical & Testing Center, Sichuan University, Chengdu, Sichuan 610065, P. R. China; § Metrology Research Centre, National Research Council Canada, Ottawa, Ontario K1A 0R6, Canada; ∥ Institute of Atomic and Molecular Physics, Sichuan University, Chengdu, Sichuan 610065, P. R. China; ⊥ Laboratory of Ethnopharmacology, West China School of Medicine, West China Hospital, Sichuan University, Chengdu, Sichuan 610065, P. R. China

## Abstract

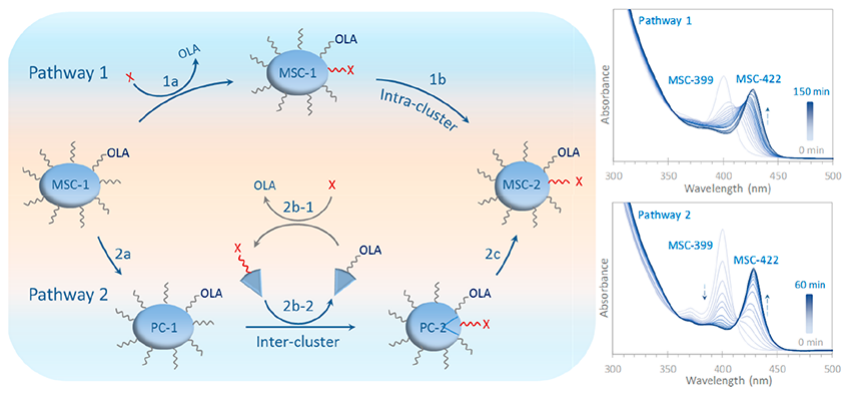

The field of isomerization reactions for colloidal semiconductor magic-size clusters (MSCs) remains largely unexplored. Here, we show that MSCs isomerize via two fundamental pathways that are regulated by the acidity and amount of an incoming ligand, with CdTeSe as the model system. When MSC-399 isomerizes to MSC-422 at room temperature, the peak red-shift from 399 to 422 nm is continuous (pathway 1) and/or stepwise (pathway 2) as monitored in situ and in real time by optical absorption spectroscopy. We propose that pathway 1 is direct, with intracluster configuration changes and a relatively large energy barrier. Pathway 2 is indirect, assisted by the MSC precursor compounds (PCs), from MSC-399 to PC-399 to PC-422 to MSC-422. Pathway 1 is activated when PC-422 to MSC-422 is suppressed. Our findings unambiguously suggest that when a change occurs directly on a nanospecies, its absorption peak continuously shifts. The present study provides an in-depth understanding of the transformative behavior of MSCs via ligand-induced isomerization upon external chemical stimuli.

## Abstract 2

Two isomerization pathways are identified for colloidal semiconductor MSCs, similar to organics. Pathway 1 is intracluster. Pathway 2 is indirect-assisted by the precursor compounds (PCs) of the MSCs.

## Introduction

Colloidal semiconductor magic-size clusters (MSCs) are expected to bridge the gap between molecules and quantum dots (QDs) toward extended bulk materials, and to enable a more thorough understanding of the evolution of physicochemical properties that occurs with the increasing numbers of atoms linked by chemical bonds.^1−7^ Similar to colloidal metal clusters,^8−12^ the magic-size nanostructures consist of an inorganic core and an organic ligand shell. The core has a precise atomic composition, while the shell consists of surface ligands that provide colloidal stability when the MSCs are dispersed in liquids. For metal clusters, ligand-induced pseudoisomerization has been demonstrated, such as for thiolated gold clusters Au_28_(S-R)_20_^11^ and Au_25_(S-R)_18_.^12^

Research on MSCs is still in an early stage,^4,5,7,13−29^ and transformation of MSCs via isomerization has received marginal attention with fundamental questions unaddressed such as how MSCs isomerize under external stimuli of chemicals.^30−38^ Being precise in composition at the atomic level, MSCs can be considered to be analogous to organic molecules as well. The reactions that chemically transform the QDs have similarities with traditional organic reactions, although isomerization is not addressed.^39^ Nonetheless, isomerization is ubiquitous in organic molecules.^40−44^ When a molecule of an open-chain unsaturated hydrocarbon isomerizes into another open-chain hydrocarbon or a cyclic one, the process can be reversible or not. Such isomerization may involve a shift of one atom (such as hydrogen) or a group of atoms (such as −CH_3_) from one carbon atom to another. The shift of one hydrogen atom may undergo an intra- or intermolecular path.^42−44^ More details on the pathways are developed in Figure S1.1.

The state-of-art of nanocrystal synthesis is similar to that of organic synthesis about 100 years ago. At that time, organic synthesis was an empirical art with useful but poorly understood procedures developed. At present, optical absorption spectroscopy is employed to monitor the evolution and transformation of MSCs, in a manner similar to that used in the initial study of the isomerization of organic molecules. The MSCs are labeled by their characteristic exciton peak wavelength expressed in nanometers (nm) in optical absorption. Just four groups of isomers have been hitherto proposed for II–VI metal chalcogenide (ME) MSCs [based on characterization including energy dispersive X-ray spectroscopy (EDS), X-ray diffraction (XRD), and transmission electron microscopy (TEM), together with kinetics studies of their transformations]. They are CdS MSC-311 and MSC-322;^30,31,33^ CdSe MSC-361, MSC-391, and MSC-415;^33,34^ CdTe MSC-371, MSC-417, and MSC-448;^32,35^ and CdTeSe MSC-399 and MSC-422.^36^

When one type of MSC (MSC-1) isomerizes to another type (MSC-2) at room temperature, a stepwise shift of optical absorption spectra is generally observed in situ and in real time. With a constant core composition, MSC-1 decreases in absorbance strength and MSC-2 increases. The discrete pattern of the spectrum change usually displays a characteristic isosbestic point located between the two absorption peaks of MSC-1 and MSC-2, without intermediate absorptions. MSCs have their precursor compounds (PC),^5,21−38^ and the isomerization pathway is modeled as going through their PCs.^5,32,35,36^ That is from MSC-1 to PC-1, then to PC-2, and finally to MSC-2. Being relatively optically transparent, the PCs do not absorb at the peak position of their counterpart MSCs or to longer wavelengths. The PC-assisted evolution and transformation of the four groups of isomers is presented in Figure S1.2.

Very recently, an in situ, real-time examination by optical absorption spectroscopy reveals a strikingly different pathway for the room-temperature evolution of CdTe MSC-488 from CdTe MSC-448.^37,38^ An unexpected continuous red-shift pattern is observed, in addition to the stepwise one described above. However, the study begins with prenucleation stage samples of CdTe QDs, also called induction period (IP) samples that contain no QDs but the PC for CdTe MSC-371, together with CdTe monomers and fragments (Mo/Fr). When the IP sample is dispersed in a mixture of toluene (Tol) and alcohol (ROH such as butanol), the transformation starts from CdTe PC-371 to MSC-448 via PC-448. The observed CdTe MSC-448 to MSC-488 transformation presents a continuous (pathway 1) or a discrete (pathway 2) red-shift pattern. Pathway 1 contains an intracluster process of uninterrupted configuration changes, with the optical trademark of intermediate clusters detected. Pathway 2 proceeds via the corresponding PCs that are optically transparent. The limitation of the isomerization study that does not start with CdTe MSC-448 is presented in Figure S1.3. It is not certain whether the intracluster and intercluster pathways are generally valid for the isomerization reaction of semiconductor MSCs, and whether they are induced by surface ligands.

**Scheme 1:**
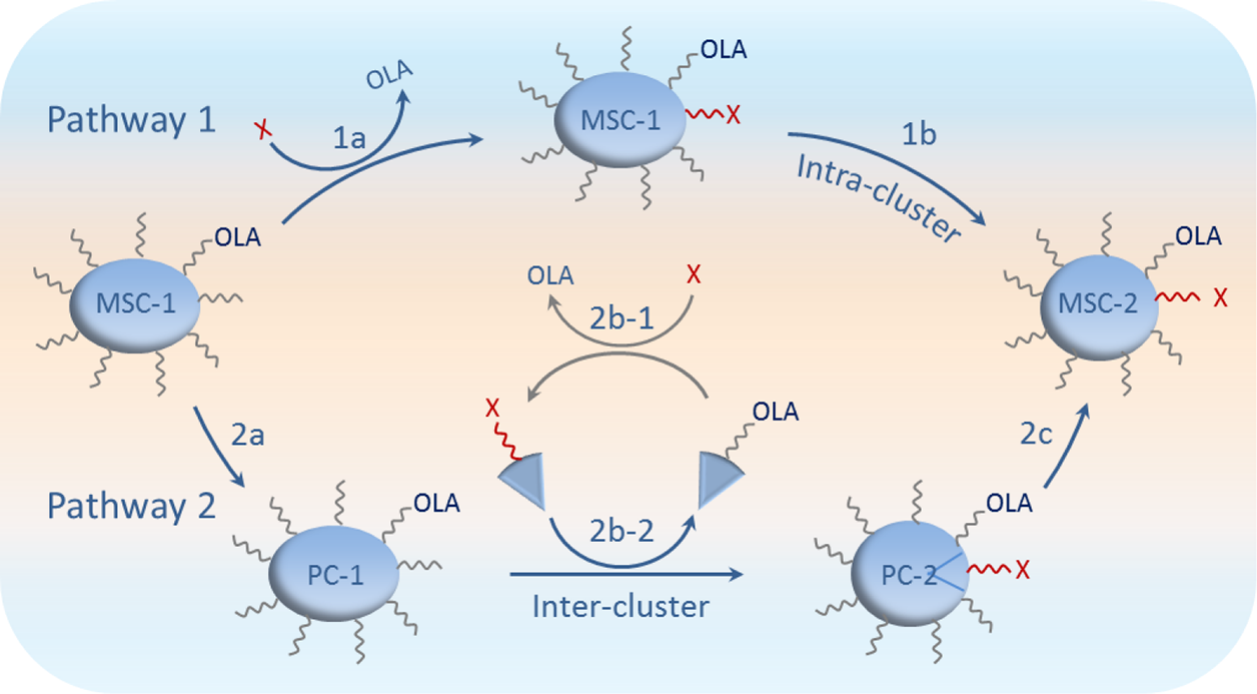
Representation of Two Isomerization Pathways Leading to Colloidal Semiconductor MSC-2 from MSC-1 Pathway 1 is initiated by ligand exchange reaction of 1a and the intermediate clusters are followed by optical absorption spectroscopy in situ and in real time. Pathway 1 is direct in conjunction with intracluster configuration changes (1b) and a relatively large energy barrier. Pathway 2 contains three key steps and displays a distinctive isosbestic point located between the absorption peak positions of MSC-1 and MSC-2. Pathway 2 is indirect-assisted by corresponding PCs. The ligand exchange reaction of eq 2b-1 gives impetus to the substitution reaction of eq 2b-2, and PC-1 transforms to PC-2 (step 2b). When CdTeSe MSC-399 is dispersed in a mixture of Tol and an incoming ligand (X) [such as phenol (PhOH) or C_2_H_5_COOH (PAc)] at room temperature, MSC-399 actively transforms to PC-399 (step 2a) and then to PC-422 (step 2b). Step 2c (PC-422 to MSC-422) is halted such as when the amount or acidity of the incoming ligand is relatively large; pathway 1 is activated passively with a continuous red-shift to 422 nm from 399 nm. When the amount or acidity is relatively small, step 2c is on the go, and pathway 2 is followed with a step-wise red-shift and an isosbestic point at 414 nm. Under many circumstances, isomerization proceeds via pathway 1 first. The consumption of the incoming ligand and the accumulation of PC-422 activate step 2c, and pathway 2 takes over; none of MSC-399 tolerates pathway 1 anymore. The earlier that step 2c starts, the less the amount of MSC-399 that follows pathway 1, and the more distinctive is the isosbestic point. Our findings indicate that when a change directly occurs on a nanospecies, its optical absorption shifts continuously.

Here, we present a unified picture for isomerization in II–VI colloidal semiconductor MSCs (at room temperature under external stimuli of chemicals) (Scheme 1). This general principle is demonstrated with CdTeSe MSCs as a model system. We note that this first continuous red-shift in the CdTeSe MSC-399 to MSC-422 isomerization is fundamentally different from that in the CdTe MSC-448 to MSC-488 isomerization,^37^ as elucidated in Figure S1.3. The present study begins with CdTeSe MSC-399, while the previous work starts with CdTe PC-371 instead of CdTe MSC-448. Therefore, the present study brings more fundamental insights on the two pathways of MSC isomerization, together with more implications (as discussed before the Conclusion in Figures S10-1–S10-4). As for organic molecules,^40−44^ the MSC isomerization occurs following intracluster (pathway 1) and intercluster (pathway 2) pathways. Furthermore, like metal clusters,^11,12^ the isomerization here is induced by surface ligands; isomerization instead of quasi-isomerization or pseudoisomerization is used in the present study. Binary CdTe and CdSe IP samples are synthesized separately in two reactions of cadmium acetate [Cd(OAc)_2_] and tri-*n*-octylphosphine chalcogenide (ETOP, E = Te or Se) in a primary amine, oleylamine (OLA).^21,33−35^ The binary IP samples are mixed at room temperature; during a one-day incubation, CdTeSe MSC-399 (MSC-1) evolves with OLA as the surface ligand.^27,28,36^ See the Experimental Section in the Supporting Information for details, and Figure S1.4 for an explanation of the MSC-399 formation. In mixtures of Tol and an incoming ligand (X), such as phenol (PhOH, *M*_w_ = 94, *K*_a_ = 10^–10.0^) or propionic acid (C_2_H_5_COOH, *M*_w_ = 74, *K*_a_ = 10^–4.9^), CdTeSe MSC-399 isomerizes to CdTeSe MSC-422 (MSC-2) at room temperature. PhOH and C_2_H_5_COOH are the X-type ligand.^45^ Based on a correlation of experimental observations, we propose that the isomerization proceeds via a direct, intracluster process (pathway 1) when the incoming ligand has a relatively large amount or acidity. The isomerization is induced by the ligand exchange reaction of eq 1a with the respective incoming and outgoing ligands of X and OLA. Subsequently, the continuous, intracluster configuration change is followed toward the product CdTeSe MSC-422, described by eq 1b.
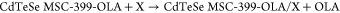
1a
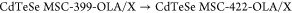
1bWhen the amount or acidity of the incoming ligand is relatively small, the isomerization proceeds via an indirect, intercluster process that involves the formation and transformation of the corresponding PCs (pathway 2). Pathway 2 encompasses three key steps, which are represented in sequence by eqs 2a–2c. As denoted by eq 2a, step 2a is the MSC-399 to PC-399 isomerization. In step 2b CdTeSe PC-399-OLA transforms to CdTeSe PC-422-OLA/X, initiated by the ligand exchange reaction of eq 2b-1 that is followed by the substitution reaction of eq 2b-2. The net reaction of eq 2b-1 and eq 2b-2 is PC-399-OLA + X to PC-422-OLA/X + OLA. Step 2c is the PC-422 to MSC-422 isomerization that is indicated by eq 2c.
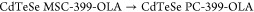
2a
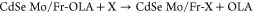
2b-1
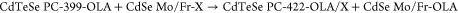
2b-2
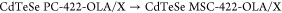
2cBoth pathway 1 and pathway 2 involve ligand exchange reactions, with the energy barrier of the latter being relatively small. When step 2c is halted, pathway 1 becomes active passively. When step 2c is activated due to the consumption of the incoming ligand and the accumulation of PC-422, pathway 2 takes over and no MSC-399 endures pathway 1 anymore. In such a case, a distortion of the isosbestic point of pathway 2 is observed; the earlier that step 2c begins, the more regular is the isosbestic point. Our findings provide an in-depth understanding of room-temperature isomerization of MSCs, that can proceed directly via configuration changes (pathway 1) and/or indirectly through their PCs (pathway 2). The presence of an incoming ligand promotes the evolution of the more thermodynamically favored product MSC-2. Indeed, the amount and acidity of the incoming ligand regulate how the starting cluster MSC-1 isomerizes.

## Results and Discussion

**Figure 1:**
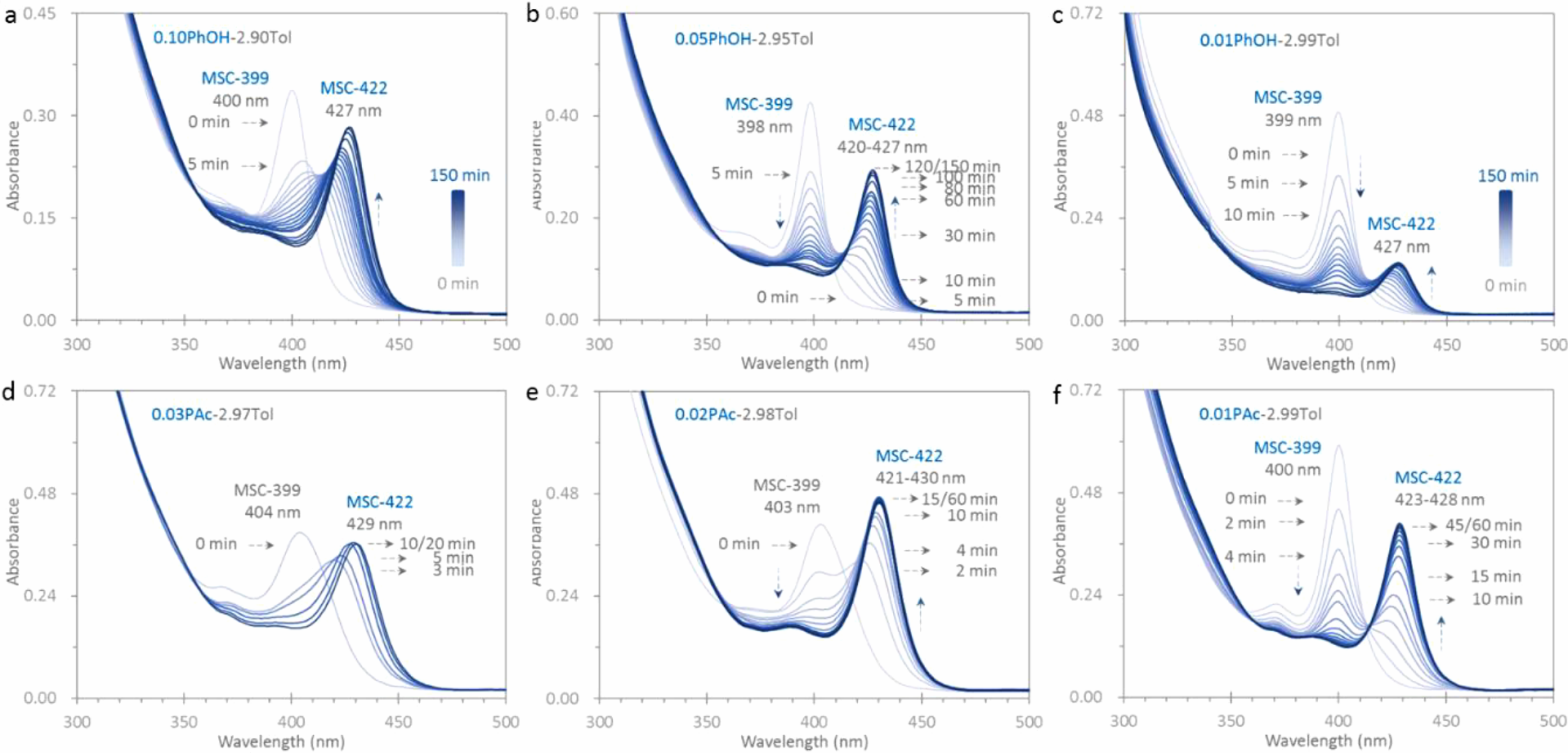
Optical absorption spectroscopy monitoring in situ and in real time the MSC-399 to MSC-422 isomerization at room temperature. The MSC-399 sample (120 μL) is dispersed in 3.00 mL mixtures of Tol and PhOH (top panel) or PAc (bottom panel). The amount of PhOH is 0.10 (a, 1.14 mmol), 0.05 (b), and 0.01 (c) mL, and that of PAc is 0.03 (d, 0.4 mmol), 0.02 (e), and 0.01 (f) mL. In parts a and d, pathway 1 is followed. In parts c and f, pathway 2 is followed. In parts b and e, pathway 2 follows pathway 1. PAc is a stronger acid than PhOH, and pathway 1 is more likely to be followed at the same amount. The amount and acidity of the incoming ligand play an important role in determining which pathway is followed.

In the following whenever we make reference to a specific step such as step 2a, it is pathway 2 that is relevant as depicted in Scheme 1. When the one-day incubated mixture of the CdTe and CdSe IP samples (that contains CdTeSe MSC-399) is dispersed, optical absorption spectroscopy records the reaction in situ and in real time. Table S1 summarizes the optical density (OD) and full width at half-maximum (fwhm) of the reactant MSC-399 and the product MSC-422. Dispersed in mixtures of Tol and phenol (PhOH) (Figure 1a–c) or in mixtures of Tol and propionic acid (PAc, C_2_H_5_COOH) (Figure 1d–f), MSC-399 transforms to MSC-422 with two distinct features. At a relatively large amount of PhOH/PAc (Figure 1a,d), a continuous red-shift pattern (pathway 1) dominates. At a relatively small amount on the other hand (Figure 1c,f), a stepwise red-shift pattern (pathway 2) is likely to be followed. Careful observation indicates that pathway 2 can follow pathway 1 in occurring (Figure 1b,e). The presence of PhOH/PAc enables the PC-399 to PC-422 transformation (step 2b), while it slows the subsequent PC-422 to MSC-422 transformation (step 2c) that is the rate-limiting one (Figure 1c).

**Figure 2:**
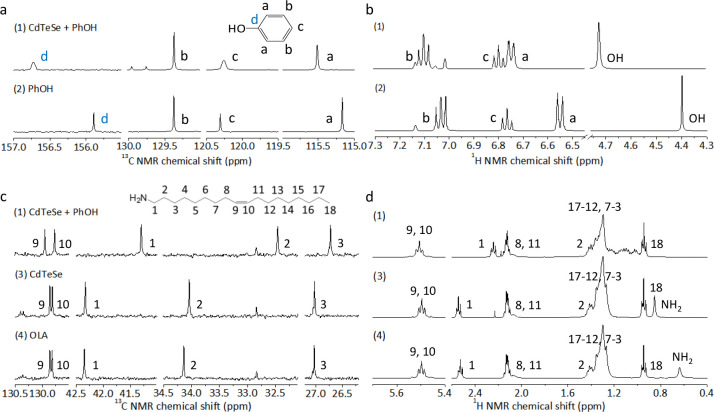
^13^C and ^1^H NMR study in Tol-*d*_8_ (580 μL) at room temperature. The samples are the MSC-399 sample (24 μL) mixed with PhOH (20 μL) (traces 1), PhOH (20 μL, traces 2), the MSC-399 sample (24 μL, traces 3), and OLA (20 μL, traces 4). The concentrations of MSC-399 and PhOH of sample 1 are similar to those shown in Figure 1a. The resonance signals in parts a and b are for PhOH, while those in parts c and d are for OLA. From trace 2 to trace 1 for part a, the d- and a-site carbon atoms downfield shift 0.83 and 0.34 ppm, respectively, and the d signal broadens; for part b, the −OH group, a-, b-, and c-site hydrogen atoms downfield shift 0.33, 0.20, 0.06, and 0.03 ppm, respectively. In parts c and d, traces 3 and 4 look similar (except for the −NH_2_ signals). From trace 3 to trace 1 for part c, the 1-, 2-, and 3-site carbon atoms upfield shift by 1.04, 1.58, and 0.29 ppm, respectively; for part d, the 1-site hydrogen atom upfield shifts by 0.31 ppm. That PhOH interacts with MSC-399 as shown by eq 1a explains the downfield shift in parts a and b and the upfield shift in parts c and d. The inductive effect of the phenyl ring detected with the electron-withdrawing effect is worthy of notice.

**Figure 3:**
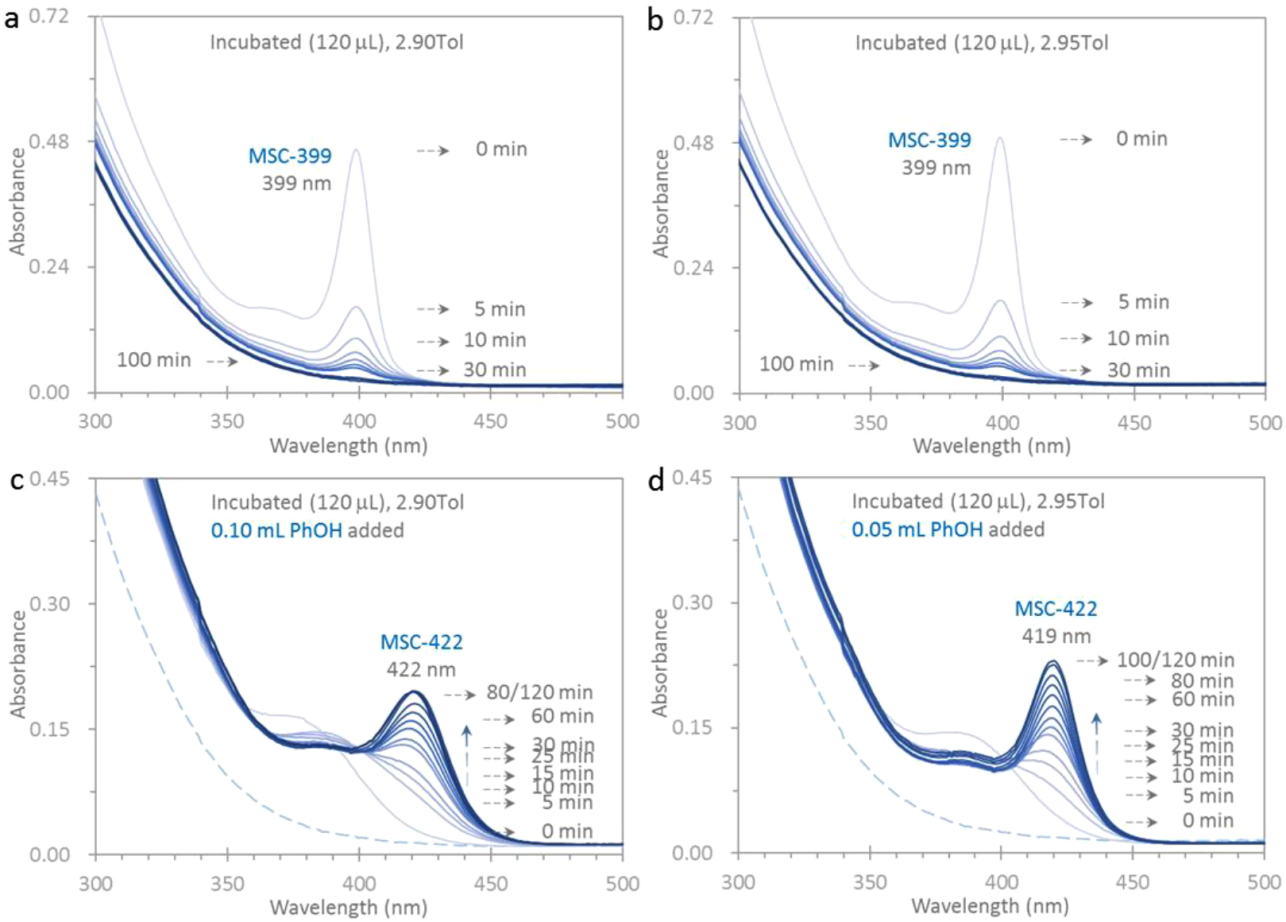
Optical absorption spectra collected in situ and in real time from two dispersions. The MSC-399 sample (120 μL) is dispersed in 2.90 (a) and 2.95 (b) mL of Tol. MSC-399 decreases in strength via step 2a to PC-399; this process completes in 80 min. After the spectrum collection at 100 min, PhOH is added with the amount of 0.10 mL (c) and 0.05 mL (d) to have the total volume of 3.00 mL. The concentrations of MSC-399 and PhOH are similar to those shown in Figure 1a,b, respectively. The dashed traces in parts c and d are the 100 min spectra in parts a and b, respectively. MSC-422 evolves with an OD of 0.18 and an fwhm of 31 nm at 80 min in dispersion c, and a larger OD of 0.21 and a smaller fwhm of 22 nm at 100 min in dispersion d with a smaller PhOH amount. Accordingly, PhOH is required for step 2b (the PC-399 to PC-422 transformation), but inhibits step 2c (the PC-422 to MSC-422 transformation).

**Figure 4:**
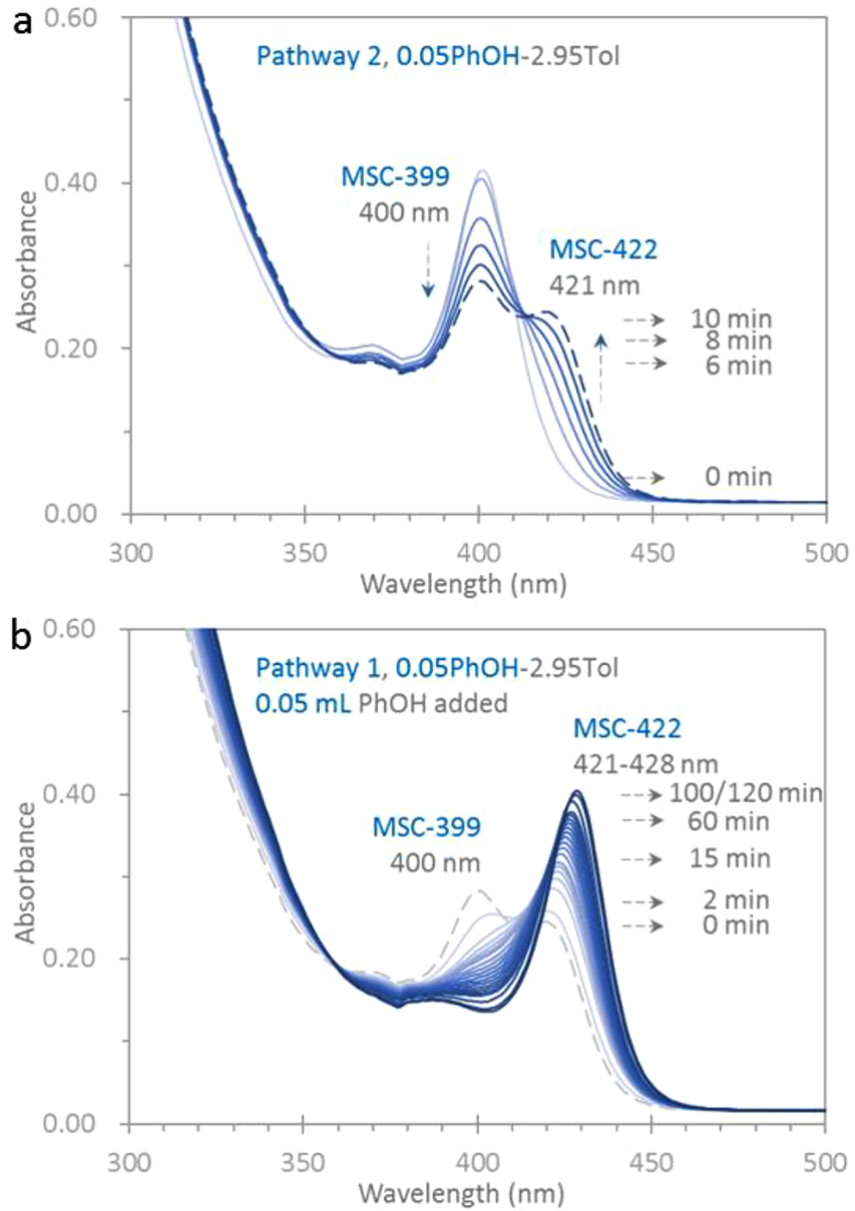
Optical absorption spectroscopy study showing how pathway 1 is activated by extra PhOH. The dispersion (a) is similar to that shown in Figure 1b. Six spectra are collected in situ from 0 to 10 min with an interval of 2 min. The strength of MSC-399 decreases while that of MSC-422 increases. The isomerization follows pathway 2 with an isosbestic point at 414 nm. After the collection of the spectrum at 10 min (dashed traces), PhOH (0.05 mL) is added (b). The total PhOH amount is 0.10 mL, similar to that shown in Figure 1a. Nineteen spectra are collected in situ with intervals of 2 min from 0 to 10 min, 5 min from 15 to 60 min, and 20 min from 80 to 120 min. After the PhOH addition, MSC-399 isomerizes to MSC-422 via pathway 1 since step 2c is halted.

**Figure 5:**
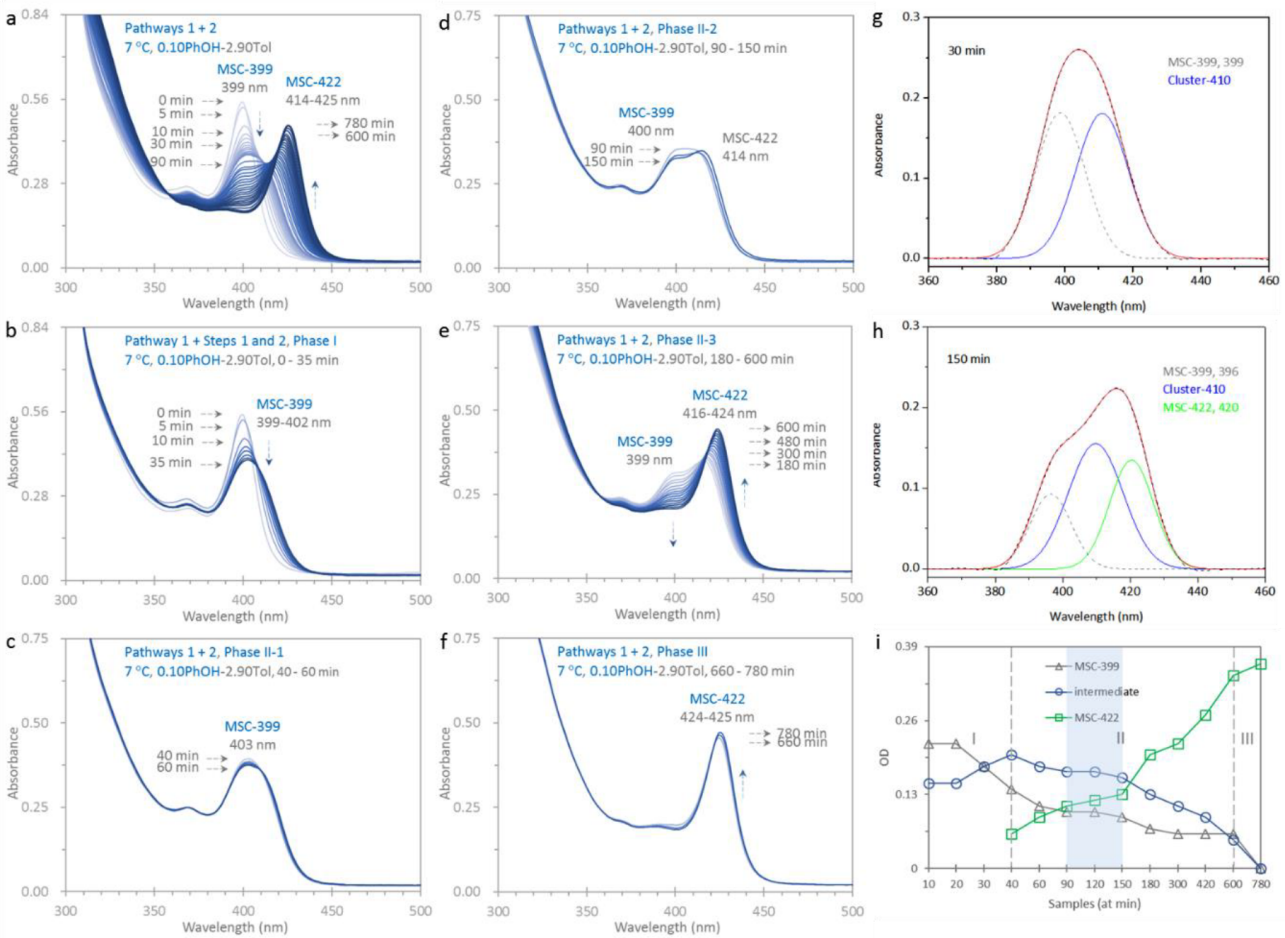
Isomerization at 7 °C explored by optical absorption spectroscopy. The dispersion is similar to that for Figure 1a but is at 7 °C. 34 spectra are collected in situ up to 780 min (a), and those from 0 to 35 min (b, eight traces phase I), 40 to 60 min (c, five traces phase II-1), 90 to 150 min (d, three traces phase II-2), 180 to 600 min (e, fifteen traces phase II-3), and 660 to 780 min (f, three traces phase III) are presented separately. From 0 to 35 min (b), the peak decreases in strength with line broadening and a slight red-shift in position. From 40 to 60 min (c), a red-side shoulder evolves sluggishly. From 90 to 150 min (d), the shoulder becomes a peak indicating the presence of MSC-422. From 180 to 600 min (e), MSC-422 increases in strength with a peak position at 425 nm. From 660 to 780 min (f), only MSC-422 is seen. The deconvolution of the 30 and 150 min spectra (dashed black with baseline subtraction) is shown in parts g and h, respectively. For the fitted Gaussian peaks, the dashed gray traces are for MSC-399 and the blue traces for the intermediate cluster in pathway 1, and the green trace is for MSC-422. The black dashed traces represent the absorption spectra. The red, superimposed traces are the results for the Gaussian peaks, which overlap well with the dashed black spectra. Part i summarizes the deconvolution result with gray triangular symbols for MSC-399, blue circular symbols for the intermediate cluster, and green square symbols for MSC-422. Based on the evolution of MSC-422, the isomerization proceeds in three phases. The vertical lines are guide for the eye. It is evident that pathway 2 is activated at 7 °C (after 40 min), while pathway 1 is followed only at room temperature (Figure 1a).

The results from ^13^C and ^1^H nuclear magnetic resonance (NMR) spectroscopy clearly show the electron-withdrawing inductive effect of the phenyl ring, suggesting that PhOH interacts with MSC-399 and ligand exchange occurs (Figure 2). An incoming ligand is required for step 2b (Figure 3), but it restrains step 2c (Figures 3 and 4). When step 2c is stopped, MSC-399 isomerizes via pathway 1 passively (Figure 4). Pathway 2 has a relatively low energy barrier; when step 2c is active, MSC-399 isomerizes only via pathway 2 (Figure 5). To further understand the chemistry of the ligand exchange, we examine other alcohols and phenols, which are methanol (MeOH) (*M*_w_ = 32, *K*_a_ = 10^–15.5^) (Figure S6), ethanol (EtOH) (*M*_w_ = 46, *K*_a_ = 10^–16.0^) (Figure S7), cyclohexanol (C_6_H_11_OH) (*M*_w_ = 100, *K*_a_ = 10^–16.0^), 1,2,3,4-tetrahydro-1-naphthol (PhC_4_H_7_OH) (*M*_w_ = 148, *K*_a_ = 10^–14.3^), phenylmethanol (PhCH_2_OH) (*M*_w_ = 94, *K*_a_ = 10^–14.4^) (Figure S8-1), and o/m-cresol (o/m-CH_3_PhOH) (*M*_w_ = 108, *K*_a_ = 10^–10.3^/10^–10.1^) (Figure S8-2). Also, acetic acid (CH_3_COOH) (*M*_w_ = 60, *K*_a_ = 10^–4.8^) and formic acid (HCOOH) (*M*_w_ = 46, *K*_a_ = 10^–3.7^) (Figure S9) are studied. All the evidence indicates that pathway 1 is more likely to be followed when an incoming ligand has a relatively large amount and acidity.

## Amount and Acidity of Incoming Ligands Regulating Isomerization Pathways

In Figure 1 we present the optical absorption spectra collected in situ and in real time from six dispersions at room temperature. Each dispersion contains the CdTeSe MSC-399 sample (120 μL) in a mixture (3.00 mL) of Tol and PhOH (top panel, a–c) and of Tol and PAc (bottom panel, d–f). The amounts of PhOH are 0.10 mL (a, 1.14 mmol), 0.05 mL (b), and 0.01 mL (c), while those of PAc are 0.03 mL (d, 0.40 mmol), 0.02 mL (e), and 0.01 mL (f). For each dispersion in the top panel, 17 spectra are collected with intervals of 5 min from 0 to 60 and 20 min from 60 to 120 min, and at 150 min. For the three dispersions in the bottom panel, spectra are collected up to 20 min (d) and 60 min (e and f). From dispersion d, six spectra are collected at 0, 3, 5, 10, 15, and 20 min. In the case of dispersions e and f, 16 spectra are collected with intervals of 2 min from 0 to 10 min, and 5 min from 10 to 60 min.

The PhOH amount decreases from dispersions a to c. At 0 min for dispersion a, an absorption peaking at 400 nm is observed, indicating the presence of CdTeSe MSC-399 that has an OD of 0.33 and an fwhm of 20 nm. At 5 min, the peak red-shifts to 404 nm together with a significant decrease in strength (OD = 0.22) and an increase in fwhm (30 nm). At 10 min, the peak further red-shifts to 407 nm with an OD of 0.21 and an fwhm of 30 nm. At 20 min, the peak is at 414 nm with an OD of 0.20 and an fwhm of 27 nm. Afterward, the peak uninterruptedly red-shifts with an incessant increase in strength and a continuous decrease in fwhm. At 120 min, the peak arrives at 426 nm, with an OD of 0.27 and an fwhm of 21 nm. At 150 min, the peak is located at 427 nm with almost no change in OD or in fwhm. Thus, CdTeSe MSC-422 seems to be well established via pathway 1. Figure S1-1 summarizes the change of the strength (OD) and fwhm during the course of the isomerization from MSC-399 to MSC-422. For dispersions with the larger PhOH amounts of 0.30 and 0.50 mL, a similar pattern of the isomerization is seen (Figure S1-2).

For dispersion b at 0 min, the presence of CdTeSe MSC-399 is observed that peaks at 398 nm with a larger OD of 0.41 and a much narrower fwhm of 15 nm. At 5 min, the peak position of MSC-399 is unchanged, but its strength (OD = 0.28) decreases by ∼32%; moreover, an absorption band, like a bump or shoulder, on the red side of MSC-399 is seen. At 10 min, the strength of MSC-399 (OD = 0.24) decreases by about 41%, while the bump evolves into a peak at around 420 nm indicating the presence of MSC-422. Afterward, MSC-399 keeps decreasing, while MSC-422 increases with its peak position that slightly red-shifts. At 120 min, MSC-399 disappears and MSC-422 peaks at 427 nm. Compared with that in dispersion a, MSC-422 has a similar OD of 0.27 and a relatively narrow fwhm of 16 nm. From 120 to 150 min, little change is observed. An isosbestic point appears to be located at 414 nm. In this case the isomerization from MSC-399 to MSC-422 mainly follows a different path, namely, pathway 2. The evolution of MSC-422 follows first-order reaction kinetics behavior (Figure S1-3), which is also seen for the dispersion that has the PhOH amount of 0.03 mL (Figure S1-3). The rate constants obtained are 0.02 min^–1^. The kinetics study suggests that step 2c and thus pathway 2 starts mainly in 20 min.

In dispersion c (that has the smallest PhOH amount of 0.01 mL), CdTeSe MSC-399 peaks at 399 nm at 0 min with the largest OD of 0.47 and the smallest fwhm of 14 nm. At 5 and 10 min, the OD of MSC-399 decreases by about 32% and 49% to 0.32 and 0.24, respectively. As time goes by, MSC-399 keeps decreasing; at 150 min, MSC-399 disappears almost completely. MSC-422 evolves at 80 min, peaking at 427 nm with a small OD of 0.12 and an fwhm of 19 nm. From 80 to 150 min, MSC-422 changes little. We note that MSC-399 decreases faster than it does in dispersion b, and MSC-422 evolves more sluggishly compared to the same process in dispersion b. It is apparent that the MSC-399 to MSC-422 isomerization proceeds following pathway 2.

For dispersion d, an absorption is observed at 0 min, peaking at 404 nm with an OD of 0.39 and a large fwhm of 36 nm. MSC-399 isomerizes faster to MSC-422 via pathway 1 than it does in dispersion a. At 20 min, MSC-422 has a larger OD of 0.33 and a broader fwhm of 37 nm. For dispersion e, an absorption peaking at 403 nm is seen at 0 min, with an OD of 0.39 and an fwhm of 35 nm. At 2 min, this peak strength decreases (OD = 0.30), and another absorption peak appears at 421 nm suggesting the presence of MSC-422. Afterward, the 403 peak keeps decreasing while MSC-422 increases and red-shifts. At 15 min, the 403 peak disappears almost completely, while MSC-422 peaks at 430 nm with an OD of 0.43 and an fwhm of 23 nm. From 15 to 60 min, MSC-422 changes little. The isomerization proceeds first via pathways 1 and then follows pathway 2.

For dispersion f with the smallest amount (0.01 mL) of PAc, the isosbestic point at 414 nm is extremely distinctive, suggesting that the isomerization path is dominated by pathway 2 with little if any contribution from pathway 1 at the beginning. At 0 min, CdTeSe MSC-399 peaking at 400 nm is observed with an OD of 0.57 and an fwhm of 16 nm. At 2 min, MSC-399 decreases by about 26% in strength (OD = 0.43), and a bump develops on its red side. At 4 min, the OD of MSC-399 decreases by about 42% to 0.33, while a peak at 420 nm develops from the bump. At 6 min, MSC-399 has an OD of 0.27, and MSC-422 increases in strength peaking at 423 nm. At 45 min, MSC-399 disappears and MSC-422 peaks at 428 nm with an OD of 0.37 and an fwhm of 19 nm. From 45 to 60 min, little change is observed. The evolution of MSC-422 follows first-order reaction kinetics with a larger rate constant of 0.08 min^–1^ (Figure S1-4).

The experimental results shown in Figure 1 indicate that the dispersion environment has a significant influence on the optical absorption spectrum that is collected immediately (at 0 min) after the MSC-399 sample is dispersed. When the dispersion contains a relatively large amount of PhOH (Figure 1a) or PAc (Figure 1d), MSC-399 exhibits a relatively large fwhm of 20 or 36 nm, respectively. We argue that MSC-399 undergoes an intracluster configuration change (pathway 1) toward MSC-422 (with 21 or 37 nm of fwhm). The intermediate clusters that form on the way to the product MSC-422 are detected successively by optical absorption spectroscopy. With a relatively small amount of PhOH or PAc, MSC-399 displays a relatively large OD of 0.41 (Figure 1b) or 0.47 (Figure 1c) or 0.57 (Figure 1f), and a relatively small fwhm of 15 nm (Figure 1b) or 14 nm (Figure 1c) or 16 nm (Figure 1f), respectively. The MSC-399 to MSC-422 isomerization proceeds via pathway 2 that is PC-assisted. The strength of MSC-399 gradually decreases, while that of MSC-422 monotonically increases to the OD of 0.27 (Figure 1b) or 0.37 (Figure 1f) and the fwhm of 16 nm (Figure 1b) or 19 nm (Figure 1f). The stepwise red-shift pattern displays an isosbestic point at 414 nm. The less that pathway 1 progresses (Figure 1f), the more distinctive is the isosbestic point.

For pathway 2 with the three key steps, we argue that it is necessary for the quantity of an incoming ligand to be sufficient but not too large. Equation 2b-2 of step 2b requires CdSe Mo/Fr-X that is produced by the process of eq 2b-1. When the amount of the incoming ligand is insufficient for eq 2b-1 to be followed, only a limited amount of MSC-422 evolves. The MSC-422 amount in Figure 1c is the smallest. On the other hand, when the incoming ligand amount is sufficient for eq 2b-1 but too large for step 2c (eq 2c) to occur, the evolution of MSC-422 can only proceed via pathway 1 (Figure 1a,d). That the incoming ligand interacts with the MSC-399 sample (Figure 2) and suppresses step 2c (Figures 3 and 4) is illustrated further in the following material.

## NMR Study for the Interaction between PhOH and MSC-399

We use NMR spectroscopy for further understanding of the interaction between the incoming ligand PhOH and CdTeSe MSC-399. In Figure 2 we show ^13^C NMR spectra (parts a and c) and ^1^H NMR spectra (parts b and d) collected at room temperature. The mixture that is studied contains the MSC-399 sample (24 μL) and PhOH (20 μL) in Tol-*d*_8_ (580 μL) (traces 1). With regard to the concentrations of MSC-399 and PhOH, this mixture is similar to that whose results are shown in Figure 1a. The NMR spectra from this mixture (traces 1) are compared with those of PhOH (20 μL, traces 2), the MSC-399 sample (24 μL, traces 3), and OLA (20 μL, traces 4), all of which are placed in 580 μL of Tol-*d*_8_.

The ranges of chemical shift presented in parts a and b highlight the resonance signals of the incoming ligand PhOH (top panel), while those in parts c and d pertain to the outgoing ligand OLA (bottom panel). The meta-, ortho-, and para-position carbon atoms on the benzene ring are respectively labeled as a–c, with d for the carbon atom that is bonded to the hydroxyl group (−OH). The carbon atoms on OLA are numbered from 1 to 18 starting from the atom bonded to the −NH_2_ group. The resonance signals are assigned to the carbon and corresponding bonded hydrogen atoms of PhOH and OLA. We note that the PhOH concentration affects the ^1^H resonance signals of the a-site and the −OH group atoms; the larger the concentration is, the larger the downfield shift is. The b- and c-site atoms are affected little (Figure S2-1). The underlying cause for the concentration effect is hydrogen bonding between PhOH molecules. The resonance signal for the −OH group is further elaborated with the assistance of a small amount of D_2_O (Figure S2-2). For another view of the assignment of the a- to c-site atoms, we also apply 2D ^1^H–^13^C heteronuclear single quantum correlation (HSQC) NMR (Figure S2-3).

The resonance signals detected are consistent with the ligand exchange reaction of eq 1a. A detailed description of the spectra is presented in Note S1a, with more detailed information in Figure S2-4. In part a from trace 2 to trace 1, the ^13^C resonance signals of the d and a carbon atoms shift 0.83 ppm (155.89 to 156.72) and 0.34 ppm (115.17 to 115.51) in the downfield direction, respectively. The shift is attributed to the interaction between the −OH group and the Cd atom, that results in the decrease of the electron density for the d carbon atom. With the inductive effect, the electron density of the a carbon atom decreases as well but to a smaller degree. The spectrum line width of the d carbon atom in trace 1 is larger than that in trace 2, suggesting a smaller relaxation time *T*_2_,^21,27,33^ that can be attributed to a slower motion of a larger species (MSC) and the chemical exchange among various species such as the bonded and free PhOH molecules.

In part b from trace 2 to trace 1, the ^1^H resonance signals shift downfield with the degree decreasing from the −OH group (4.40 to 4.73 = 0.33 ppm), a-site (6.54 to 6.74 = 0.20 ppm), b-site (7.04 to 7.10 = 0.06 ppm), to c-site (6.77 to 6.80 = 0.03 ppm) hydrogen atoms. The smaller the distance is to the −OH group, the greater the shift is in the downfield direction. The interaction between the −OH group and the Cd atom (eq 1a) results in the downfield shift of the ^13^C (part a) and ^1^H (part b) signals from traces 2 to traces 1.

In parts c and d, traces 3 and 4 are similar. From trace 3 to trace 1, the ^13^C resonance signals of the 1, 2, and 3 carbon atoms upfield shift 1.04 ppm (42.34 to 41.30), 1.58 ppm (34.04 to 32.46), and 0.29 ppm (27.02 to 26.73). Meanwhile, the ^1^H resonance signals of the 1-site hydrogen shift in the upfield direction by 0.31 ppm (2.55 to 2.24). The upfield shift of the ^13^C (part c) and ^1^H (part d) signals from trace 3 to trace 1 is consistent with the reaction of eq 1a.

## The Role of Incoming Ligands in Enabling Step 2b and Suppressing Step 2c

The starting cluster CdTeSe MSC-399 is produced prior to dispersing. When CdTeSe MSC-399 is dispersed, two reactions are probably competing with each other (Scheme 1 and Figure S3-1). One is the ligand change reaction of eq 1a. The other is the isomerization reaction of eq 2a. To study further how the incoming ligand regulates the isomerization, CdTeSe MSC-399 is dispersed in Tol, in which the reaction of eq 1a does not take place but the one associated with eq 2a does.

CdTeSe MSC-399 disappears in Tol via step 2a. After the disappearance, the evolution of MSC-422 is seen upon the addition of PhOH (Figure 3) or PAc (Figure S3-2). In Figure 3, we present the absorption spectra collected in situ and in real time from two dispersions made with the MSC-399 sample (120 μL) in 2.90 (a) and 2.95 mL (b) of Tol (the top panel). From each dispersion, 12 spectra are collected at intervals of 5 min from 0 to 30 min, 10 min from 30 to 60 min, and 20 min from 60 to 100 min (Figure S3-3). Only nine of the spectra are presented for each of the two dispersions. MSC-399 keeps decreasing and almost disappears at 80 min, indicating that the reaction of eq 2a is practically complete. At 100 min, two spectra are collected from the two dispersions, followed by the addition of 0.10 and 0.05 mL of PhOH to dispersions a and b, respectively.

From each of the resulting dispersions, 13 spectra are collected at intervals of 5 min from 0 to 15 min, at 25 min, 10 min from 30 to 80 min, at 100, and at 120 min. The spectra are respectively shown in parts c and d (the bottom panel), together with the dashed traces for the 100 min points prior to the PhOH addition. Upon the addition of 0.10 mL of PhOH (c), a broad peak at ∼380 nm appears at 0 min; at 80 min, MSC-422 peaks at 422 nm and reaches its maximum strength with an OD of 0.18 and an fwhm of 31 nm. From 80 to 120 min, little further change occurs. Upon the addition of 0.05 mL of PhOH (d), a broad peak at ∼390 nm is also presented at 0 min; at 100 min, MSC-422 reaches a maximum strength with an OD of 0.21 and an fwhm of 23 nm while peaking 419 nm. For times up to 120 min, there is little change to this peak. A more detailed description for the spectrum change is presented in Note S2.

In dispersions a and b, MSC-399 in Tol transforms to PC-399 (step 2a). Without PhOH, PC-422 does not form (via step 2b), and thus, no MSC-422 evolves (via step 2c). The underlying cause is that the ligand exchange reaction described by eq 2b-1 does not occur. When PhOH is present, the reaction of eq 2b-1 takes place in dispersions c and d, resulting in the formation of PC-422 via the reaction of eq 2b-2. Dispersion c has more PhOH (0.10 mL) than dispersion d has (0.05 mL). At 120 min, MSC-422 in dispersion c develops with a smaller OD and a larger fwhm than in dispersion d. That the amount of the incoming ligand in dispersion plays an instrumental role for how MSC-422 evolves is worthy of notice.

Similar results are obtained with two other dispersions containing the MSC-399 sample (60 μL) in 2.90 and 2.97 mL of Tol; the amount of PhOH added after the disappearance of MSC-399 is 0.10 and 0.03 mL, respectively (Figure S3-4). For the addition of 0.03 and 0.02 mL of PAc after the disappearance of MSC-399 (120 μL) in 2.97 and 2.98 mL of Tol, respectively, MSC-422 with a larger OD and a narrower fwhm is detected upon the latter addition (Figure S3-2). Accordingly, the incoming ligand enables step 2b to occur while suppressing step 2c.

We note that the added PhOH amounts, 0.10 mL (Figure 3c) and 0.05 mL (Figure 3d), are similar to those presented in Figure 1a,b, respectively. Meanwhile, the added PAc amounts, 0.03 mL (Figure S3-2c) and 0.02 mL (Figure S3-2d), are similar to those presented in Figure 1d,e, respectively. The approach associated with Figure 1 to MSC-422 seems to be more efficient. The approach associated with Figure 3, although less efficient, provides convincing information regarding the pathway investigation (Figure S3-5).

## More Incoming Ligands Hampering Step 2c while Pathway 1 Is Being Activated

To further investigate whether the incoming ligand inhibits step 2c, we add supplemental PhOH or PAc to PhOH-containing or PAc-containing dispersions of MSC-399, respectively. Before the addition, the isomerization to MSC-422 is proceeding via pathway 2. After the addition, pathway 1 is activated. In Figure 4 we present the optical absorption spectra that are collected in situ and in real time from one dispersion before (a) and after (b) the PhOH addition. Dispersion a contains the MSC-399 sample (120 μL) in a mixture of 0.05 mL of PhOH and 2.95 mL of Tol, similar to that shown in Figure 1b. Six spectra are collected at an interval of 2 min within 10 min. At 0 min, MSC-399 has an OD of 0.40 and an fwhm of 24 nm peaking at 400 nm. At 2 min, MSC-399 has an OD of 0.39. MSC-399 decreases in strength by about 14% at 4 min to 35% at 10 min, with ODs of 0.34 and 0.26, respectively. A red side bump evolves at 2 min and develops into a peak at 420 nm at 6 min. At 10 min, MSC-422 peaks at 421 nm with an OD of 0.23 and an estimated fwhm of 25 nm. With an isosbestic point at ∼414 nm, MSC-399 isomerizes to MSC-422 via pathway 2.

After collecting the spectrum (dashed trace) at 10 min, 0.05 mL of PhOH is added. The resulting dispersion (b) has the total PhOH amount of 0.10 mL, similar to that in Figure 1a. Nineteen spectra are collected from dispersion b at intervals of 2 min from 0 to 10 min, 5 min from 15 to 60 min, and 20 min from 80 to 120 min. At 0 min, a peak at 404 nm with an OD of 0.23 is seen, which is transformed from the original 400 nm peak with an OD of 0.26 (dashed trace). Meanwhile, MSC-422 peaks at 421 nm with an OD of 0.24. At 2 min, MSC-399 almost vanishes, and MSC-422 red-shifts to 422 nm with an OD of 0.27. Afterward, the strength at 400 nm keeps decreasing, and MSC-422 increases and red-shifts. At 100 min, peaking at 428 nm, MSC-422 reaches its maximum strength with an OD of 0.38 and an fwhm of 21 nm. Up to 120 min, little further change is observed.

The supplementary addition of the incoming ligand halts step 2c, and thus, pathway 1 is activated. Two other examples of PhOH addition are shown in Figure S4-1. This effect also occurs with PAc (Figure S4-2). When the MSC-399 to MSC-422 isomerization is following pathway 2 in two mixtures (3.00 mL each) of Tol and PAc (of 0.01 and 0.005 mL), 0.02 mL of PAc is added. Before the PAc addition, the PAc amount of 0.01 mL is similar to that used in Figure 1f; after the addition, the PAc amount of 0.03 mL is similar to that used in Figure 1d.

## Lower Temperatures Promoting Step 2c to Complete Pathway 2

To compare the energy barrier of the two pathways, the isomerization is studied at temperatures other than room temperature. A dispersion is made similar to that shown in Figure 1a but at 7 °C; 34 optical absorption spectra are collected in situ and in real time at intervals of 5 min from 0 to 60 min, 30 min from 60 to 600 min, and 60 min from 600 to 780 min. The full set of the spectra is shown in Figure 5a. At 0 min, MSC-399 peaking at 399 nm is observed with an OD of 0.53 and an fwhm of 19 nm, while MSC-399 in Figure 1a peaks at 400 nm with an OD of 0.33 and an fwhm of 20 nm. From 660 to 780 min (f), MSC-399 disappears completely, while MSC-422 peaks at 425 nm with an OD of 0.45 and an fwhm of 20 nm. At 150 min in Figure 1a, MSC-422 peaks at 427 nm with an OD of 0.27 and an fwhm of 21 nm. At 7 °C, it is significant that MSC-399 (at 0 min) and MSC-422 (at 660 min) display larger strengths than at room temperature, for the reason that step 2a is less favored and step 2c more favored.

The temporal evolution of the spectra is apparently different from that shown in Figure 1a. Based on the spectral characteristics, the spectra from 0 to 35 min (phase I), 40 to 60 min (phase II-1), 90 to 150 min (phase II-2), 180 to 600 min (phase II-3), and 660 to 780 min (phase III) are respectively highlighted in parts b–f of Figure 5. Each spectrum shown in parts b (phase I) and f (phase III) seems to consist of a single peak, and that in parts c–e appears to have two peaks. In part b, the peak continuously decreases in strength, broadens in fwhm, and red-shifts in position; at 35 min, the peak red-shifts to 402 nm with a smaller OD of 0.38 and a larger fwhm of 35 nm. In part c, the 40 min absorption peaks at 403 nm with an OD of 0.37 and an fwhm of 36 nm. Up to 60 min, this peak slightly decreases in strength with little change in the position. However, a red-side shoulder appears to develop. In part d, the peak becomes flat at 90 min; afterward, the shoulder evolves into a peak suggesting the presence of MSC-422. The MSC-422 peak becomes prominent in part e and is only seen in part f.

During the course of the isomerization at 7 °C, the existence of the different phases is worthy of notice. To obtain further understanding of the isomerization, a deconvolution is performed for more resolution of the spectra. Two, three, and one Gaussian peaks are respectively obtained with baseline subtraction and least-squares fitting, for the spectra collected from 5 to 35 min (phase I), from 40 to 600 min (phase II), and after 600 min (phase III) (Figure S5-1). Figure 5g,h respectively contains the spectra collected at 30 and 150 min (dashed black traces), together with the Gaussian peaks and their sum (red traces). The Gaussian peaks of dashed gray, blue, and green traces are for MSC-399, intermediate clusters of pathway 1, and MSC-422. The OD change of MSC-399 (gray triangular symbols), the intermediate cluster (blue circular symbols peaking at 410 nm, the position of which is similar to that obtained in Figure S1-5), and MSC-422 (green square symbols) is summarized in Figure 5i. The lines are to guide the eye.

At 7 °C, MSC-399 decreases continuously in strength (up to 660 min). In phase I without the presence of MSC-422 (prior to 40 min), MSC-399 actively and slowly transforms to PC-399 via step 2a and then to PC-422 via step 2b, and the amount of PC-422 accumulates. Meanwhile, some MSC-399 passively follows pathway 1 resulting in the intermediate cluster. The strength of the intermediate cluster increases and becomes more than that of MSC-399 after 35 min. When MSC-422 appears at 40 min via step 2c, the isomerization proceeds to phase II where pathway 2 is activated fully. While the intermediate cluster keeps transforming toward MSC-422, MSC-399 no longer follows pathway 1 but instead undergoes pathway 2. While the strength of the intermediate cluster is constant, that of MSC-422 continuously increases. Based on the relative amount of MSC-422, three substages are attributed to phase II. Before 90 min (phase II-1), the strength of MSC-422 is the smallest. From 90 to 150 min (phase II-2), the strength of MSC-422 becomes larger than that of MSC-399 and is still weaker than that of the intermediate cluster. From 180 to 600 min (phase II-3), the strength of MSC-422 is the largest. In phase III (after 600 min), only MSC-422 exists and is quite stable in strength. We note that the lack of a distinct isosbestic point is due to the presence of the intermediate cluster (from pathway 1) during the initial course of the isomerization.

That the isomerization follows pathway 2 at 7 °C (Figure 5) while it follows pathway 1 at room temperature (Figure 1a) indicates that the energy barrier of pathway 2 is smaller than that of pathway 1. With the smaller rate constant of 0.003 min^–1^ obtained (Figure S5-2), the kinetics study result agrees with our conclusion that pathway 2 is activated later at 7 °C. Isomerization reactions of organic molecules that involve a shift of one hydrogen atom from one atom to another also have two reaction pathways (Figure S1.1). The intramolecular path may encompass a three- or four-membered ring, while the intermolecular path may undergo a five- or six-membered ring assisted by a water molecule. The posited intermediates have rarely been trapped. The energy barrier of the former path is larger than that of the latter path.^41−43^ Analogously, the direct, intracluster pathway 1 has a larger energy barrier than step 2b has (with the assistance of CdSe Mo/Fr). Furthermore, step 2c can be rate-determining for pathway 2.

To examine further the two pathways, we allow the isomerization (of the Figure 1a dispersion) to proceed for 15 min at room temperature via pathway 1, and then let the isomerization continue at 7 °C (Figure S5-3). Clearly, pathway 2 is activated at 7 °C. When the isomerization occurs at 10 °C from the beginning, pathway 2 is followed as well after pathway 1 (Figure S5-4). For PAc-containing dispersions, temperature affects the isomerization pathway similarly; pathway 2 is followed at 5 °C (Figure S5-5) while pathway 1 is followed at room temperature (Figure 1d).

We note that higher temperatures favor step 1 more. When MSC-399 is dispersed, there is a portion that actively transforms to PC-399 via step 2a. The higher the temperature is, the greater the amount that is transformed (at 0 min). Thus, MSC-399 (at 0 min) exhibits larger strength at 7 °C (Figure 5a) than at room temperature (Figure 1a). Accordingly, step 2c (PC-422 to MSC-422) is favored more at lower temperatures. MSC-422 exhibits larger strength at 7 °C (Figure 5a at 660 min) than at room temperature (Figure 1a at 150 min).

The carbon–oxygen bond of phenol is much stronger than that of an alcohol. Phenols are stronger acids than alcohols, but are weaker acids than carboxylic acids. In addition to PhOH, alcohols and phenols with different acidity and steric hindrance are investigated; they are MeOH (Figure S6), EtOH (Figure S7), C_6_H_11_OH, PhC_4_H_7_OH, PhCH_2_OH (Figure S8-1), and o/m-CH_3_PhOH (Figure S8-2). Figure S8-3 presents ^1^H NMR of these compounds. In addition to PAc, we also have results for CH_3_COOH and HCOOH (Figure S9). The acidity of the incoming ligand indeed plays an important role in determining which pathway is followed. The larger acidity that the incoming ligand has, the more likely that the isomerization follows pathway 1.

In the study of isomerization reactions, it is helpful to reduce the number of experimental variables to a minimum. The concentrations of MSC-399 and PhOH in Figure 1a are similar to those shown in the Figure 2 NMR study (sample 1) and in the study in Figures 3–5. When process variables (including the nature of the incoming ligand) and the pathway that govern the isomerization of MSCs are understood, we will be in a stronger position to fully manipulate MSC isomerization and to better control their physicochemical properties.^6,46^ Also, the knowledge about the surface ligand chemistry will enable our synthetic capability,^29,47−49^ and the MSC isomerization pathway provides valuable information for the isomerization of ME clusters,^50^ together with organic molecules (Figure S1.1) and metal clusters.^11,12,40−44^

Our Scheme 1 suggests that whenever an MSC-1 to MSC-2 transformation follows pathway 1, a continuous shift of optical absorption is necessarily seen. When the transformation occurs via the sequence of PC-1 followed by PC-2 in pathway 2, a stepwise shift is observed instead. The PC-assisted transformation with a stepwise shift has been observed for MSC isomerization (Figure S1.2),^30,32,34−37^ a mass increase in the MSC core,^25,54^ cation exchange from ZnE to CdE MSCs,^33^ and a transition from binary to ternary MSCs.^27−29^ For the isomerization from CdTe MSC-448 to MSC-488 (Figure S1.3)^37,38^ and from CdTeSe MSC-399 to MSC-422 in the present study, the striking finding regarding the uninterrupted red-shift indicates that when a continuous shift is seen, the mother nanospecies makes the change directly. For example, a continuous red-shift behavior is expected when zero-dimension (0D) QDs grow in size due to monomer addition directly onto the mother QDs.^1^ On the other hand when a stepwise red-shift is observed, monomer addition has often been assumed to take place on CdSe 0D clusters to become larger or 2D nanoplatelets (NPLs) to become thicker.^15,19,51−54^ This assumption infers that the intermediate does not have a measurable optical absorption, but it is difficult to understand why now. The difficulty disappears, however, when this assumption is abandoned and the concept of the PC-assisted transformation is applied. See Figures S10-1–S10-4 for additional elaboration. Moreover, an isosbestic point that is regular or distorted is simulated for PC-assisted transformations, as shown by Figure S11.

## Conclusion

We have shown for the first time how colloidal semiconductor MSCs isomerize at low temperatures under external chemical stimuli (Scheme 1). Like organic molecules,^41−44^ the isomerization described here proceeds along intracluster (pathway 1) and intercluster (pathway 2) pathways. Also, as for metal clusters,^11,12^ it is the surface ligand that induces the isomerization. Pathway 1 undergoes a direct, intracluster configuration change with a relatively large energy barrier; the intermediate clusters are observed in situ and in real time by optical absorption spectroscopy. Pathway 2 contains an indirect, PC-assisted reconstruction, comprising three key steps. With CdTeSe MSCs as a model system, the starting cluster (MSC-1) CdTeSe MSC-399 in Tol actively transforms to PC-399 (step 2a); when MSC-399 vanishes, the addition of an incoming ligand (such as PhOH) results in the development of the product (MSC-2) CdTeSe MSC-422 (steps 2b and 2c). The larger the PhOH amount added, the smaller the absorption strength and the broader the absorption line of MSC-422 produced (Figure 3). The incoming ligand assists step 2b (PC-399 to PC-422) but suppresses step 2c (PC-422 to MSC-422). This interpretation of the effect of the incoming ligand is supported by the result obtained when CdTeSe MSC-399 is in a mixture containing Tol and an incoming ligand (Figure 1). In this case, the larger the PhOH/PAc amount, the smaller the absorption strength and the broader the absorption line of MSC-399. At a relatively large or small PhOH/PAc amount, MSC-399 transforms to MSC-422 via pathway 1 or pathway 2, respectively. In the former situation, step 2c is halted. When the isomerization is following pathway 2, supplementary PhOH suppresses step 2c, and pathway 1 is thus passively activated (Figure 4). At a lower temperature, step 2c is more favored; thus, pathway 2 is followed instead of pathway 1 at a later stage (Figure 5). NMR suggests that PhOH interacts with the CdTeSe MSC-399 sample, with the electron-withdrawing inductive effect of the phenyl ring significantly revealed (Figure 2). The present study provides an in-depth understanding of the isomerization pathway of MSCs. All the experimental evidence is in favor of the idea that the acidity and quantity of incoming ligands is a critical factor in controlling which isomerization pathway is followed.

## Data Availability

The data that support the findings of this study are available from the corresponding author upon reasonable request.

## Supporting Information Available

Additional optical absorption spectra, additional NMR study, and further discussions about isomerization pathways for organics, consensus not reached for the MSC composition, the difference between superimposed and offset spectrum presentations, and the significance of the continuous spectrum shift of MSCs (PDF)
