# Supplementary material for: Direct and Indirect Pathways of CdTeSe Magic-Size Cluster Isomerization Induced by Surface Ligands at Room Temperature
Source: ACS Cent Sci. 2023 Mar 8;9(3):519–30. doi: 10.1021/acscentsci.2c01394 (PMC10037450; doi:10.1021/acscentsci.2c01394)
Supplement: Supplementary file 1 — oc2c01394_si_001.pdf [file oc2c01394_si_001.pdf]

Supporting Information

Direct and Indirect Pathways of CdTeSe Magic-Size Cluster Isomerization Induced by Surface Ligands at Room Temperature

Yusha Yang,[1] Qiu Shen,[1] Chunchun Zhang,[2] Nelson Rowell,[3]

Meng Zhang,[4] Xiaoqin Chen,[1] Chaoran Luan,[*,5] Kui Yu[*,1,4]

[1]Engineering Research Center in Biomaterials, Sichuan University,

Chengdu, Sichuan, 610065, P. R. China

[2]Analytical & Testing Center, Sichuan University, Chengdu 610065, China

[3]Metrology Research Centre, National Research Council Canada,

Ottawa, Ontario K1A 0R6, Canada

[4]Institute of Atomic and Molecular Physics, Sichuan University,

Chengdu, 610065 Sichuan, P. R. China

[5]Laboratory of Ethnopharmacology, West China School of Medicine,

West China Hospital, Sichuan University, Chengdu, Sichuan, 610065, P. R. China

Correspondence and requests for materials should be addressed to

C. L. (email: luanc@scu.edu.cn) or to K. Y. (email: kuiyu@scu.edu.cn)

Table of Contents

**Experimental Section**		S3
**Figure S1.1**	Intra- and inter-molecular isomerization proposed	S6
**Figure S1.2**	Four groups of MSCs with PC-assisted isomerization in	S7
**Figure S1.3**	Limitation of the CdTe study with comparison to the CdTeSe study	S10
**Figure S1.4**	Evolution of CdTeSe MSC-399 in a CdTe and CdSe mixture	S12
**Table S1**	Summary of OD and FWHM for CdTeSe MSC-399 and MSC-422	S14
**Figure S1-1**	Temporal evolution of OD and FWHM for Figure 1a	S15
**Figure S1-2**	Pathway 1 in two dispersions with 0.30 and 0.50 mL PhOH	S16
**Figure S1-3**	Kinetics study for two dispersions with 0.05 and 0.03 mL PhOH	S17
**Figure S1-4**	Kinetics study of the Figure 1f reaction	S18
**Figure S1-5**	Deconvolution of the 10 and 20 min spectra in Figure1a	S19
**Note S1**	Detailed description for the Figure 2 spectra	S20
**Figure S2-1**	^{1}H NMR of PhOH with various concentrations in Tol-d_8	S21
**Figure S2-2**	^{1}H NMR of PhOH before and after D_2O addition	S22
**Figure S2-3**	^{1}H-^{13}C HSQC of a PhOH and MSC-399 mixture and of PhOH	S23
**Figure S2-4**	^{13}C and ^{1}H NMR	S24
**Note S2**	Description for the Figure 3 spectra	S25
**Figure S3-1**	Schematic drawing to understand the MSC-422 evolution	S26
**Figure S3-2**	PAc-induced CdTeSe MSC-422 in one Tol dispersion	S27
**Figure S3-3**	Full set of the spectra for Figure 3a and Figure 3b	S28
**Figure S3-4**	PhOH-induced CdTeSe MSC-422 from two Tol dispersions	S29
**Figure S3-5**	Comparison of Figure 1 and Figure 3 approaches to MSC-422	S30
**Figure S4-1**	Pathway 1 activated by extra PhOH addition	S31
**Figure S4-2**	Pathway 1 activated by extra PAc addition	S33
**Figure S5-1**	Deconvolution of representative Figure 5a spectra	S34
**Figure S5-2**	Kinetic study of MSC-422 evolution in the Figure 5 reaction	S36
**Figure S5-3**	The Figure 1a dispersion at 25 °C and then 7 °C	S37
**Figure S5-4**	The Figure 1a dispersion at 10 °C	S38
**Figure S5-5**	Two Figure 1d dispersions at 7 °C and 5 °C	S39
**Figure S6-1**	Three MeOH-containing dispersions	S40
**Figure S6-2**	Pathway 1 in four MeOH-containing dispersions	S42
**Figure S6-3**	Pathway 2 after Pathway 1 in four MeOH-containing dispersions	S43
**Figure S6-4**	Pathway 2 in four MeOH-containing dispersions	S44
**Figure S6-5**	MeOH-induced CdTeSe MSC-422 in two Tol dispersions	S46

**Figure S7-1**	Three EtOH-containing dispersions	S47
**Figure S7-2**	EtOH-induced CdTeSe MSC-422 in two Tol dispersions	S49
**Figure S8-1**	Six dispersions with six alcohols	S50
**Figure S8-2**	Four dispersions of o-CH_3PhOH and m-CH_3PhOH	S52
**Figure S8-3**	^1H NMR of six alcohols	S53
**Figure S9**	Four dispersions with 0.02 and 0.01 mL of HAc and MAc	S54
**Figure S10-1**	Examples of ongoing effects for the MSC composition study	S55
**Figure S10-2**	Examples of reported shifts with corresponding transformations	S57
**Figure S10-3**	PC-assisted transformation argued for a step-wise shift	S60
**Figure S10-4**	Offset Figures 1d-f and 5a spectra and others	S62
**Figure S11**	Simulation of optical absorption spectra in PC-assisted transformations	S64
**REFERENCES**		S68

Experimental Section

Materials. Cadmium acetate ($Cd(OAc)_2{\cdot}2H_2O$, 99.99%), oleylamine (OLA, 70%), and tri-*n*-octylphosphine (TOP, 90.0%) were obtained from Aldrich. Tellurium powder (Te, 99.99%) and selenium powder (Se, 99.99%) were purchased from Alfa Aesar. Phenol (PhOH, 99%, M_w = 94, $K_a = 10^{-10.0}$, pK_a = 10.0), cyclohexanol ($C_6H_{11}OH$, 99%, M_w = 100, $K_a = 10^{-16.0}$, pK_a = 16.0), 1,2,3,4 –tetrahydro-1-naphthol (PhC_4H_7OH, 97%, M_w = 148, $K_a = 10^{-14.3}$, pK_a = 14.3), benzyl alcohol or phenylmethanol ($PhCH_2OH$, 99%, M_w = 94, $K_a = 10^{-14.4}$, pK_a = 14.4), and o/m-cresol (o/m-CH_3PhOH, 99%, M_w = 108, $K_a = 10^{-10.3}/10^{-10.1}$, pK_a = 10.1-10.3) were from Adamas. Toluene (Tol, 99.5%), methanol (MeOH, 99.5%, M_w = 32, $K_a = 10^{-15.5}$, pK_a = 15.5), ethanol (EtOH, 99.55%, M_w = 46, $K_a = 10^{-16.0}$, pK_a = 16.0), formic acid (HCOOH, 88%, M_w = 46, $K_a = 10^{-3.7}$, pK_a = 3.7), acetic acid (CH_3COOH, 99.5%, M_w = 60, $K_a = 10^{-4.8}$, pK_a = 4.8), and propionic acid (PAc, 99.5%, M_w = 74, $K_a = 10^{-4.9}$, pK_a = 4.9) were obtained from Chengdu Kelong. Toluene-d_8 (Tol-d_8, 99.5%,) was bought from Cambridge Isotope Laboratories. Eight alcohols and three acids are studied as the chemical added with the value of pKa in the range of 3.7 to 16.0. All chemicals were used as received without further purifications.

$Cd(OAc)_2$/OLA stock solution preparation. The $Cd(OAc)_2$/OLA stock solution was prepared from a reaction of $Cd(OAc)_2 \cdot 2H_2O$ (1.60 g, 6.00 mmol) and OLA (46.70 g). Both chemicals were mixed in a 250 mL three-necked flask at room temperature. The resulting mixture was evacuated for about 10 min, followed by purging with N_2. Such an evacuation-purging circle was repeated for additional two times. Subsequently, the mixture was heated to 80 °C under a N_2 atmosphere, followed by evacuation for an hour until no bubbles were observed. The resulting mixture was then filled with N_2 and was further heated to 120 °C and was evacuated for one hour until no bubbles were observed. Under a N_2 atmosphere, the reaction batch was cooled to room temperature. The resulting clear solution with a color of light yellow was obtained as the Cd precursor stock solution that could be used for 10 times.

TeTOP and SeTOP stock solution preparation. TeTOP and SeTOP stock solutions were prepared with a feed molar ratio of 4TOP to 1E (E= Te or Se). Te powder (0.19 g, 1.50 mmol) or Se powder (0.12 g, 1.50 mmol) was mixed with TOP (2.47 g, 6.00 mmol) in a 50 mL three-necked flask at room temperature. The resulting mixture was evacuated until no bubbles were observed, followed by purging with the N_2. Such an evacuation-purging circle was repeated for additional two times. For the TeTOP reaction, the solution was further heated to 300 °C under a N_2 atmosphere and was kept for 30 min to achieve a bright orange solution. After cooling to room temperature, a transparent and light-yellow solution was obtained as a TeTOP stock solution. For the SeTOP reaction, the solution was stirred at room temperature for 30 min under a N_2 atmosphere. The resulting transparent and colorless solution was obtained as a SeTOP stock solution. Both stock solutions were stored in a glove box, each of which could be used for 10 times.

CdTe and CdSe IP sample preparation. The CdE (E = Te or Se) IP sample was prepared from a reaction of $Cd(OAc)_2$/OLA and ETOP in OLA with a feed molar ratio of 4Cd to 1E and a feed E concentration of 30 mmol/kg. The $Cd(OAc)_2$/OLA stock solution (0.60 mmol, 4.83 g) was placed in a 50 mL three-necked flask at room temperature and was heated to 120 °C under a N_2 atmosphere. Subsequently, TeTOP or SeTOP (0.15 mmol, 300 μL) was added to the $Cd(OAc)_2$/OLA solution. The reaction was heated to 130 °C or 140 °C for 30 min for CdTe or CdSe IP sample, respectively.

In situ real time optical absorption measurements. The measurements were performed on Hitachi UH4150 and Hitachi U-2910 spectrometers. The spectra were usually collected in the range from 290 to 600 nm with an interval of 1 nm. Quartz cuvettes (from Hellma Analytics) with the light path of 10 mm and with a volume of 3.5 mL (standard QS cells) were used. The background measurements were the corresponding solvents (3.00 mL), which include Tol and its mixture with an incoming ligand. For the measurement, a certain amount of our incubated sample so called the CdTeSe MSC-399 sample (such as 120 μL) was dispersed in a mixture of Tol (3.00 mL) and the incoming ligand (such as PhOH or CH_3CH_2COOH).

^{13}C and ^{1}H nuclear magnetic resonance (NMR) measurements. The ^{13}C and ^{1}H NMR spectra were collected with a Bruker AV II-400 MHz spectrometer at room temperature, with Tol-d_8 as a chemical shift reference. Spectra were analyzed with the MestReNova software.

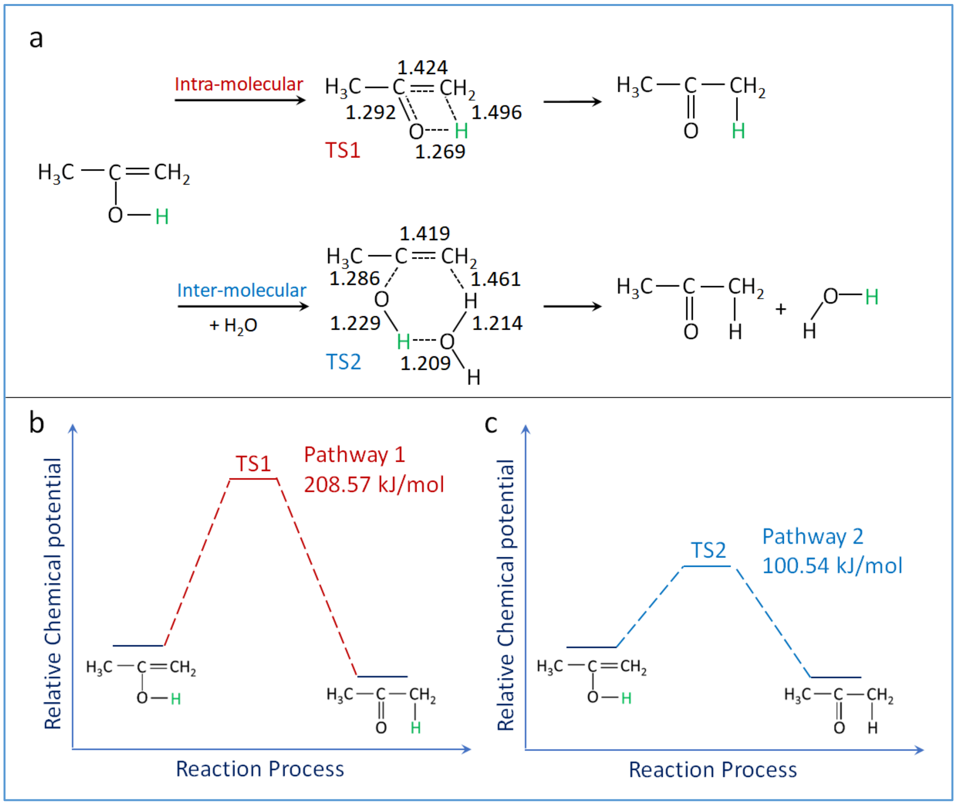

Figure S1.1. Two distinct pathways proposed for Keto-Enol Tautomerism.[1] The isomerization involves hydrogen migration. The intramolecular pathway has the transition state (TS1) of a 4-memebered ring, with an energy barrier of 208.57 kJ/mol. The intermolecular pathway has the transition state (TS2) of a 6-memebered ring, with a lower energy barrier of 100.54 kJ/mol. A water molecule assists the formation of TS2. The bond length in Å is indicated for the two TS species. As an important reaction with an invaluable role in industry, isomerization of organic molecules has been extensively explored for more than a century. Organic molecules can have a number of geometric isomers with the same chemical composition but different chemical structures.[2,3] The various isomeric forms have distinct physical and chemical properties, originally characterized by optical absorption spectroscopy.[4] It is our excitement that the two pathways find analogy in the isomerization of MSCs, as shown in the present study (Scheme 1). It is our belief that the knowledge gained about the intra-cluster and inter-cluster pathways can provide additional information for researchers that are active in physical organic chemistry, the field of which is much more well-developed than nano-chemistry.

(1) Zhu, Y.; Wei, D.; Zhang, W.; Tang, M. New theoretical insights into several common hydrogen migration reactions. *Univ. Chem.* **2014**, *29*, 52–57.
(2) Wilson, E. Isomerization of hydrocarbons. *Chem. Rev.* **1937**, *21*, 129−167.
(3) Bergmann, E. Isomerization of unsaturated hydrocarbons. *Chem. Rev.* **1941**, *29*, 529−551.
(4) Wyman, G. M. The cis-trans isomerization of conjugated compounds. *Chem. Rev*. **1955**, *55*, 625–657.

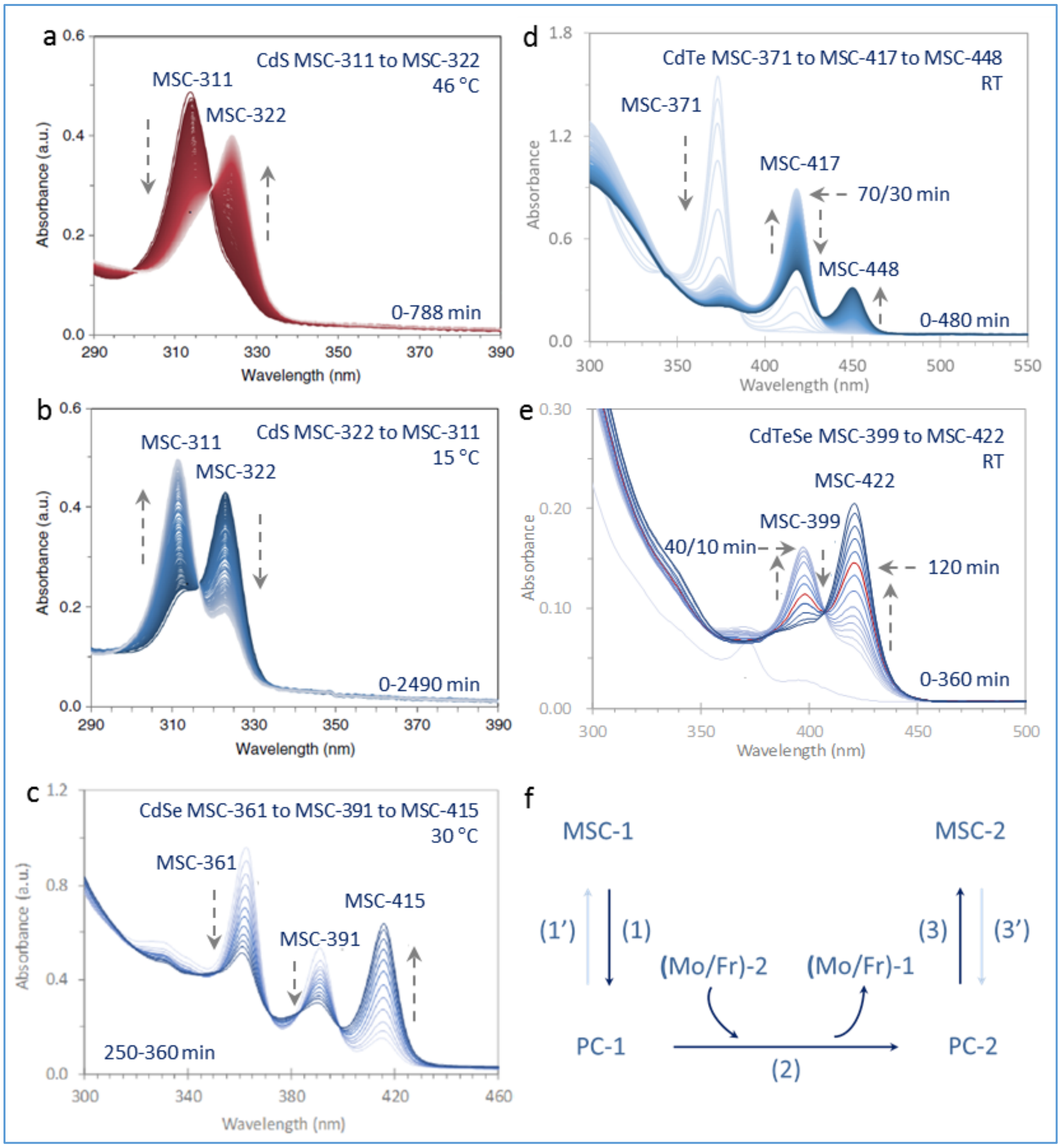

Figure S1.2. Summary of the four groups of MSC isomers identified with PC-assisted transformations.[5-9] The optical absorption spectra are collected in situ and in real time, and the discontinuous redshift patterns are observed with distinguishing isosbestic points.

(a) and (b) For CdS MSCs, the isomerization occurs at 46 °C and 15 °C in cyclohexane. The CdS IP sample is prepared with $Cd(OA)_2$ and S powder at 180 °C for 20 min.[5,9] The MSC characterization includes transmission electron microscopy (TEM), X-ray diffraction (XRD), energy dispersive X-ray spectroscopy (EDS), matrix-assisted laser desorption/ionization time-of-flight (MALDI-TOF) mass spectrometry (MS), X-ray total scattering with atomic pair distribution function (PDF) analysis, and small angle X-ray scattering (SAXS). First-order reaction kinetics behavior is demonstrated, and an Arrhenius analysis suggests that the breakage of Cd–S bonds is involved. The finding on the bond breakage is also in agreement with the PC-assisted isomerization shown in Part f.

(c) For CdSe MSCs, the isomerization happens in a mixture of Tol (1.8 mL) and octylamine (OTA,

1.2 mL) at 30 °C. The CdSe IP sample is prepared with $Cd(OAc)_2$/OLA and SeTOP at 150 °C for 20 min.[6,9]

(d) For CdTe MSCs, the isomerization takes place in a mixture of 2.6 mL Tol and 0.4 mL OTA at room temperature (RT). The CdTe IP sample is prepared with $Cd(OAc)_2$/OLA and TeTOP at 135 °C for 10 min.[7]

(e) For CdTeSe MSCs, the isomerization is seen in a mixture of 2.80 mL Tol and 0.20 mL OTA at RT. A binary CdTe IP sample (prepared with $Cd(OAc)_2$/OLA and TeTOP at 130 °C for 30 min) is mixed with a binary CdSe IP sample (prepared with $Cd(OAc)_2$/OLA and SeTOP at 140 °C for 30 min) with equal volumes. The **as-mixed** sample is dispersed in the Tol-OTA mixture. Based on the conventional characterization performed, we have concluded that CdTeSe MSC-399 and CdTeSe MSC-422 form a pair of isomers with similar core compositions (by EDS) and slightly different structures (by XRD). It is remarkable that the present finding on the continuous transformation from MSC-399 to MSC-422 at room temperature suggests that their structures should be close enough to each other. These isomers also appear to be zero-dimension and dot-like (in TEM).[8] For the present study, we use **incubated** samples in which CdTeSe MSC-399 (as MSC-1) is developed (instead of as-mixed samples without MSC-399). This approach is fundamentally different from the one used for the previous report.[8]

(f) The PC-assisted isomerization is characterized by the MSC-1 decrease and the MSC-2 increase, with three key steps from MSC-1 to PC-1 (Step 1), then to PC-2 (Step 2), and finally to MSC-2 (Step 3). The isosbestic point located between the two cluster peak positions can be outstandingly distinct or distorted.[5-10] See Figure S11 for additional discussion of the isosbestic point. We know that to explore the fundamental law and to discover the truth in the field of natural science, rational and plausible reasoning provides a powerful and practical means. Recently, we proposed the disruptive concept that a single MSC has its counterpart PC, and the isomerization is reversible.[11-13] The isomeric pair has different core structures, with the former detectable by optical absorption spectroscopy and the latter not. The same number has been used to label a MSC and its counterpart PC. For the disruptive concept of the "non-absorbing" PC to be fully established, further study will be required. However, we have not encountered any experimental evidence that contradicts this PC $\leftrightarrow$ MSC model. The present situation in some respects is analogous to that of astronomical dark matter, which cannot be directly imaged with current technology, because it is transparent to electromagnetic radiation. In some ways we know more about what dark matter is not than what it is. For the existence of dark matter, one piece of indirect but compelling evidence is the study of galaxy rotation by Madam Vera Rubin in the 1970s.[14] Also, we know that it takes time to validate the correctness of a disruptive theory or model, together with its applicability. Furthermore, even a greater amount of time may elapse for what is an essentially correct concept to be accepted. Another example is that continental drift was posited in 1912 by Alfred Wegner (1830 – 1930), but was only generally accepted in the 1950s and 1960s.

(5) Zhang, B.; Zhu, T.; Ou, M.; Rowell, N.; Fan, H.; Han, J.; Tan, L.; Dove, M. T.; Ren, Y.; Zuo, X.; Han, S.; Zeng, J.; Yu, K. Thermally-induced reversible structural isomerization in colloidal semiconductor CdS magic-size clusters. *Nat. Commun*. **2018**, *9*, 2499.

(6) Zhu, D.; Hui, J.; Rowell, N.; Liu, Y.; Chen, Q. Y.; Steegemans, T.; Fan, H.; Zhang, M.; Yu, K. Interpreting the ultraviolet absorption in the spectrum of 415 nm-bandgap CdSe magic-size clusters. *J. Phys. Chem. Lett.* **2018**, *9*, 2818−2824.
(7) Luan, C.; Gokcinar Ö. Ö.; Rowell, N; Kreouzis, T.; Han, S.; Zhang, M.; Fan, H.; Yu, K. Evolution of two types of CdTe magic-size clusters from a single induction period sample. *J. Phys. Chem. Lett.* **2018**, *9*, 5288–5295.
(8) Yang, Y.; Li, Y.; Luan, C.; Rowell, N.; Wang, S.; Zhang, C.; Huang, W.; Chen, X.; Yu, K. Transformation pathways in colloidal CdTeSe magic-size clusters. *Angew. Chem. Int. Ed*. **2022**, *61*, e202114551.
(9) He, L.; Luan, C.; Rowell, N.; Zhang, M.; Chen, X.; Yu, K. Transformations among colloidal semiconductor magic-size clusters. *Acc. Chem. Res.* **2021**, *54*, 776−786.
(10) Williamson, C. B.; Nevers, D. R.; Nelson, A.; Hadar, I.; Banin, U.; Hanrath, T.; Robinson, R. D. Chemically reversible isomerization of inorganic clusters. *Science* **2019**, *363*, 731−735.
(11) Liu, M.; Wang, K.; Wang, L.; Han, S.; Fan, H.; Rowell, N.; Ripmeester, J. A.; Renoud, R.; Bian, F.; Zeng, J.; Yu, K. Probing intermediates of the induction period prior to nucleation and growth of semiconductor quantum dots. *Nat. Commun*. **2017**, *8*, 15467.
(12) Zhu, T.; Zhang, B.; Zhang, J.; Lu, J.; Fan, H.; Rowell, N.; Ripmeester, J. A.; Han, S.; Yu, K. Two-step nucleation of CdS magic-size nanocluster MSC-311. *Chem. Mater*. **2017**, *29*, 5727−5735.
(13) Wang, L.; Hui, J.; Tang, J.; Rowell, N.; Zhang, B.; Zhu, T.; Zhang, M.; Hao, X.; Fan, H.; Zeng, J.; Han, S.; Yu, K. Precursor self-assembly identified as a general pathway for colloidal semiconductor magic-size clusters. *Adv. Sci*. **2018**, *5*, 1800632.
(14) Rubin, V. C. Galaxy dynamics and the mass density of the universe, *Proc. Natl. Acad. Sci.* **1993**, *90*, 4814–4821.

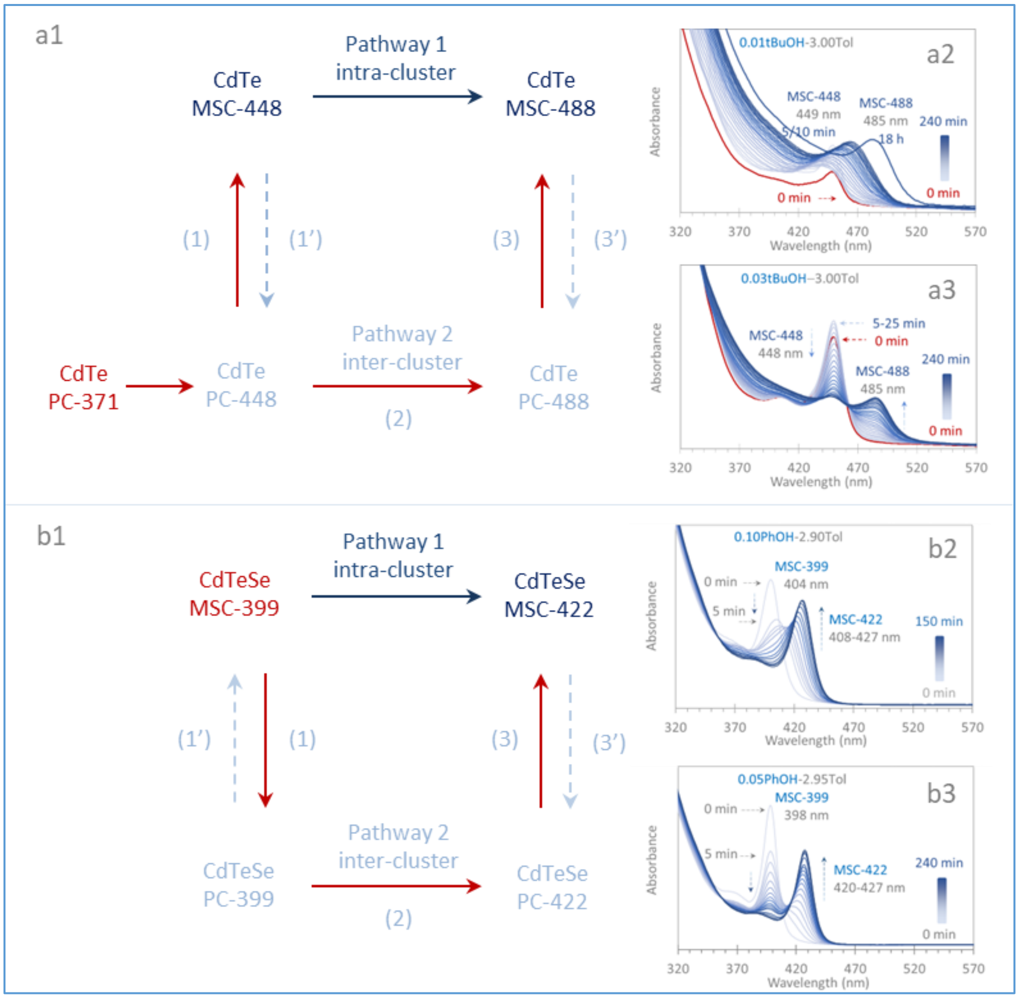

Figure S1.3. Limitation of the CdTe MSC isomerization study with comparison to the present CdTeSe study. Part a is for the CdTe MSC-448 to CdTe MSC-488 isomerization.[15] Part b shows that the present CdTeSe study is better designed for the intra-cluster and inter-cluster pathways of MSC isomerization, and is essentially different from that shown in Part a.

(a1) When one CdTe IP sample is dispersed in a mixture of Tol (3.00 mL) and alcohol, the as-synthesized CdTe PC-371 transforms to CdTe PC-448 first. Then, Step 1 to MSC-448 competes with and Step 2 to PC-488.

(a2) When t-butanol is 0.01 mL, a continuous redshift pattern (Pathway 1) is seen only after 10 min. Pathway 1 involves an intra-cluster configuration change.

(a3) When t-butanol is 0.03 mL, a discrete redshift pattern (Pathway 2) is observed only after 25 min. Pathway 2 is assisted by corresponding PCs, with three key steps (1', 2, and 3).

Accordingly, the present CdTeSe study (Part b) is fundamentally different from the CdTe study (Part a) in that

- the starting CdTeSe MSC-399 (Part b1 emphasized in red) does not have alcohol as its ligands. For CdTe (Part a1), the starting point is CdTe PC-371 (highlighted in red), and CdTe MSC-448 forms in dispersion only, with the alcohol amount affecting its formation. CdTe

MSC-448 has alcohol as its ligands.

- Pathway 1 (with a continuous redshift) is followed, when the alcohol amount is relatively large (Part b2 starting at 0 min) for CdTeSe while relatively small for CdTe (Part a2 starting at 10 min).
- Pathway 2 (with a step-wise redshift) is followed, when the alcohol amount is relatively small (Part b3 with three key steps (1, 2, and 3) starting at 0 min) for CdTeSe while relatively large for CdTe (Part a3 with three key steps (1', 2, and 3) starting at 25 min).
- The knowledge gained in present study helps us to understand more the CdTe MSC isomerization. For example, when the alcohol amount is relatively large (Part a3), the reaction of CdTe Mo/Fr-OLA + X $\Rightarrow$ CdTe Mo/Fr-X + OLA occurs; CdTe PC-448 actively transforms to CdTe PC-488 via Step 2 of Pathway 2. With enough CdTe PC-488 accumulation, Step 3 becomes activated (from CdTe PC-488 to CdTe MSC-488). Thus, Pathway 2 is activated.
- We note that the effects of X-type and L-type ligands on the MSC band edge remain unknown. For example, CdTe MSC-371 can be synthesized with $Cd(OAc)_2$/OLA in OLA and with $Cd(OA)_2$ in 1-octadecene (ODE),[11,16,17] and ZnSe MSC-299 can be obtained with $Zn(OAc)_2$/OLA in OLA and with $Zn(OA)_2$ in ODE.[13] The present subject on the pathway of MSC isomerization is intricate. It is not possible to extract a complete mechanistic insight from one study. Be that as it may, the present study provides an in-depth dynamic understanding of how MSCs evolve.

(11) Liu, M.; Wang, K.; Wang, L.; Han, S.; Fan, H.; Rowell, N.; Ripmeester, J. A.; Renoud, R.; Bian, F.; Zeng, J.; Yu, K. Probing intermediates of the induction period prior to nucleation and growth of semiconductor quantum dots. *Nat. Commun*. **2017**, *8*, 15467.

(13) Wang, L.; Hui, J.; Tang, J.; Rowell, N.; Zhang, B.; Zhu, T.; Zhang, M.; Hao, X.; Fan, H.; Zeng, J.; Han, S.; Yu, K. Precursor self-assembly identified as a general pathway for colloidal semiconductor magic-size clusters. *Adv. Sci*. **2018**, *5*, 1800632.

(15) Luan, C.; Shen, Q.; Rowell, N.; Zhang, M.; Chen, X.; Huang, W.; Yu, K. A real-time in-situ demonstration of direct and indirect transformation pathways in CdTe magic-size clusters at room temperature. *Angew. Chem. Int. Ed.* **2022**, *61*, e202205784.

(16) Luan, C.; Tang, J.; Rowell, N.; Zhang, M.; Huang, W.; Fan, H.; Yu, K. Four types of CdTe magic-size clusters from one prenucleation stage sample at room temperature. *J. Phys. Chem. Lett*. **2019**, *10*, 4345−4353.

(17) Shen, Q.; Luan, C.; Rowell, N.; Zhang, M.; Wang, K.; Willis, M.; Chen, X.; Yu, K. Reversible transformations at room temperature among three types of CdTe magic-size clusters. *Inorg. Chem*. **2021**, *60*, 4243−4251.

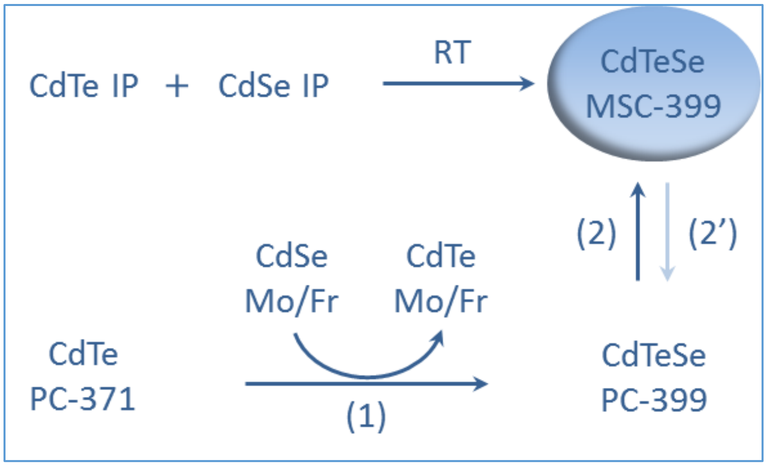

Figure S1.4. Elucidation of the evolution of CdTeSe MSC-399 at room temperature. When a CdTe IP sample is mixed with a CdSe IP sample, CdTeSe MSC-399 evolves **during incubation** at room temperature (top panel).[18,19] During incubation, CdTeSe MSC-399 with OLA as the surface ligand forms via Steps 1 and 2. Step 1 is the transformation from CdTe PC-371 to CdTeSe PC-399 that involves a substitution reaction as indicated (bottom panel). Step 2 is the isomerization from CdTeSe PC-399 to CdTeSe MSC-399. When an **as-mixed** mixture was dispersed instead of an **incubated** one,[8] the starting point was CdTe PC-371 and the continuous redshift from CdTeSe MSC-399 to CdTeSe MSC-422 nm was not observed. For the present dynamic study for the pathway of the CdTeSe MSC-399 to CdTeSe MSC-422 isomerization, only **incubated** mixtures are used. Thus, the starting point is CdTeSe MSC-399. We report two approaches. One is to disperse CdTeSe MSC-399 in a mixture of Tol and an incoming ligand (such as shown by Figures 1, 4, and 5). The other is to disperse in Tol; an incoming ligand is added only after CdTeSe MSC-399 disappear (Figure 3).

For our **dynamic** study of the isomerization under external chemical stimuli at room temperature or at 7 °C, we use in situ and real-time absorption spectroscopy to monitor continuously the process. We show for the first time how MSCs isomerize via intra-cluster (Pathway 1) and inter-cluster (Pathway 2) pathways, how the chemical added in dispersion brings about isomerization. Our findings provide an in-depth understanding of the isomerization pathway, which can be tuned by the acidity and quantity of the chemical added. In a side note, **eight** alcohols and **three** acids are studied as the chemical added with the value of pKa in the range of 3.7 to 16.0. In the main text figures, we show the data from only one alcohol (PhOH) and one acid (PAc). We have collected a large quantity of absorption spectra, but have presented a representative fraction thereof and only the more critical data.

(8) Yang, Y.; Li, Y.; Luan, C.; Rowell, N.; Wang, S.; Zhang, C.; Huang, W.; Chen, X.; Yu, K. Transformation pathways in colloidal CdTeSe magic-size clusters. *Angew. Chem. Int. Ed*. **2022**, *61*, e202114551.

(18) Gao, D.; Hao, X.; Rowell, N.; Kreouzis, T.; Lockwood, D. J.; Han, S.; Fan, H.; Zhang, H.; Zhang, C.; Jiang, Y.; Zeng, J.; Zhang, M.; Yu, K. Formation of colloidal alloy semiconductor CdTeSe magic-size clusters at room temperature. *Nat. Commun*. **2019**, *10*, 1674.

(19) Zhang, H.; Luan, C.; Gao, D.; Zhang, M.; Rowell, N.; Willis, M.; Chen, M.; Zeng, J.; Fan, H.; Huang, W.; Chen, X.; Yu, K. A room-temperature formation pathway for CdTeSe alloy magic-size clusters. *Angew. Chem. Int. Ed*. **2020**, *59*, 16943−16952.

Table S1. Summary of the optical density (OD) and the full width at half maximum (FWHM, nm) of the reactant MSC-399 and the product MSC-422.

Figures	MSC-399		MSC-422	
	OD	FWHM	OD	FWHM
**1a** (0.10PhOH)	0.33	20	0.27	21
**1b** (0.05PhOH)	0.41	15	0.27	16
**1c** (0.01PhOH)	0.47	14	0.12	19
**1d** (0.03PAc)	0.39	36	0.33	37
**1e** (0.02PAc)	0.39	35	0.43	23
**1f** (0.01PAc)	0.57	16	0.37	19
**S1-2a** (0.30PhOH)	0.28	21	0.18	30
**S1-2b** (0.50PhOH)	0.28	23	0.15	35
**S1-3b** (0.03PhOH)	0.35	15	0.25	16
**3a** (2.90Tol)	0.45	14		
**3b** (2.95Tol)	0.47	14		
**3c** (0.10PhOH added)			0.18	31
**3d** (0.05PhOH added)			0.21	22
**S3-2a** (2.97Tol)	0.28	16		
**S3-2b** (2.98Tol)	0.36	16		
**S3-2c** (0.03PAc added)			0.22	36
**S3-2d** (0.02PAc added)			0.33	30
**4a** (0.05PhOH)	0.40	24	0.23	25
**4b** (0.05PhOH added)			0.38	21
**S4-1a** (0.03PhOH)	0.51	16	0.21	24
**S4-1b** (0.04PhOH)	0.87	15	0.26	22
**S4-1c** (0.07PhOH added)			0.37	21
**S4-1d** (0.10PhOH added)			0.57	21
**5a** (0.10PhOH, 7 °C)	0.53	19	0.45	20
**S5-3** (0.10PhOH, 25 to 10 °C)	0.34	22	0.36	20

The optical density (OD) is obtained by subtracting the absorbance at 460 nm from that for MSC-399 and MSCS-422.

For the top panel of Figure 1, the MSC-422 OD is obtained based on the absorbance at 427 nm. The OD of MSC-399 is based on the absorbance at 400 nm (Figure 1a), 398 nm (Figure 1b), and 399 nm (Figure 1c). For the bottle panel, the OD of MSC-422 is based on the absorbance at 429 nm (Figure 1d), 430 nm (Figure 1e), and 428 nm (Figure 1f). The MSC-399 OD is obtained based on the absorbance at 404 nm (Figure 1d), 403 nm (Figure 1e), and 400 nm (Figure 1f).

In Figure 3, the MSC-399 OD is obtained from the absorbance at 399 nm. The MSC-422 OD is obtained from the absorbance at 422 nm (Figure 3c) and 419 nm (Figure 3d).

For Figure 4, the MSC-399 OD is obtained from the absorbance at 400 nm. The MSC-422 OD is obtained from the absorbance at 421 nm (Figure 4a) and 428 nm (Figure 4b).

For Figure 5, the MSC-399 OD is obtained from the absorbance at 399 nm. The MSC-422 OD is s obtained from the absorbance at 425 nm.

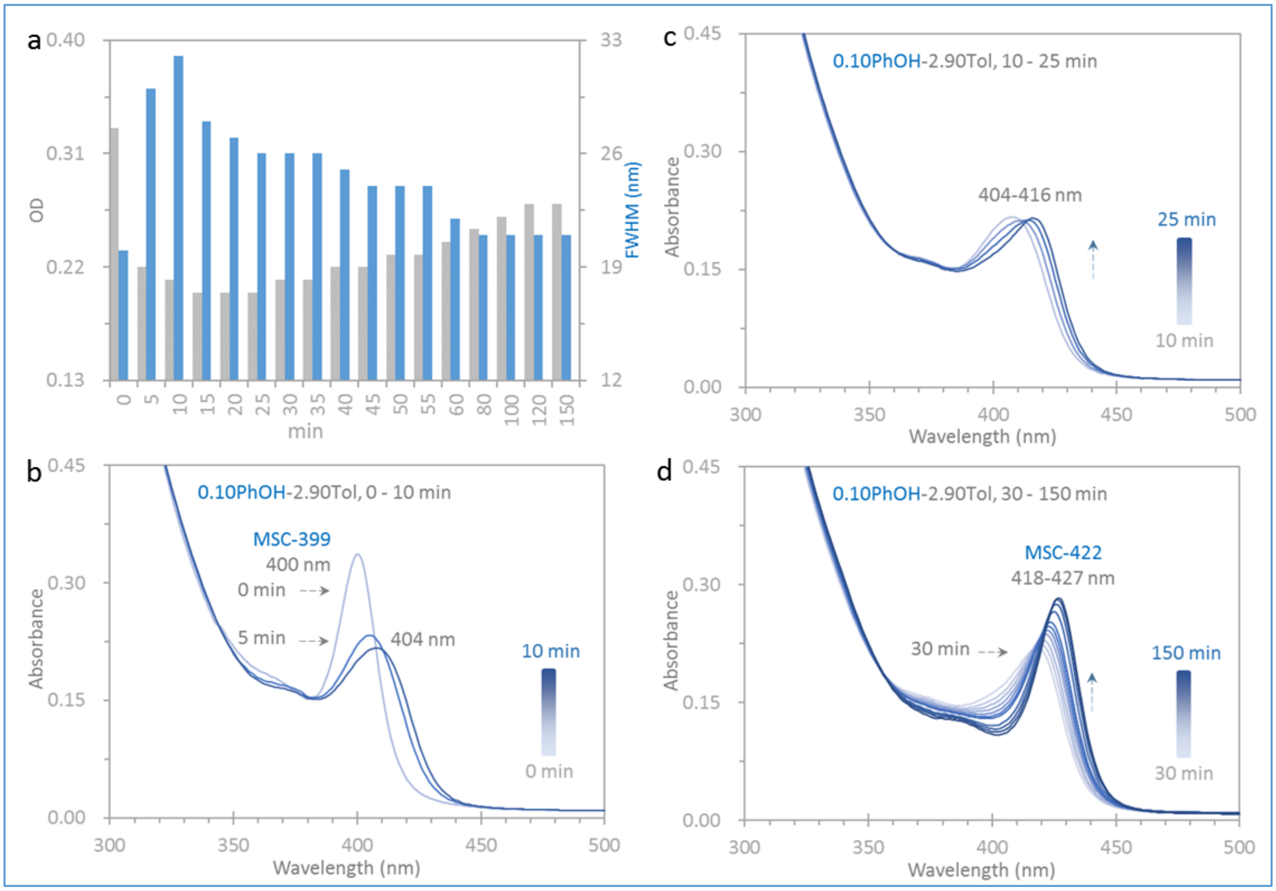

Figure S1-1. Temporal evolution of OD and FWHM for the absorption shown in Figure 1a. The OD (gray) decreases first from 0.33 to 0.20 at 20 min, then increases to 0.27 at 120 min, and becomes stable. The FWHM (blue) increases from 20 nm to 30 nm at 10 min, then decreases and becomes stable of 21 nm at 80 min. For the OD and FWHM change, the Figure 1a spectra are highlighted for those from 0 to 10 min (b, three traces), from 10 to 25 min (c, four traces), and from 30 to 150 min (d, 11 traces).

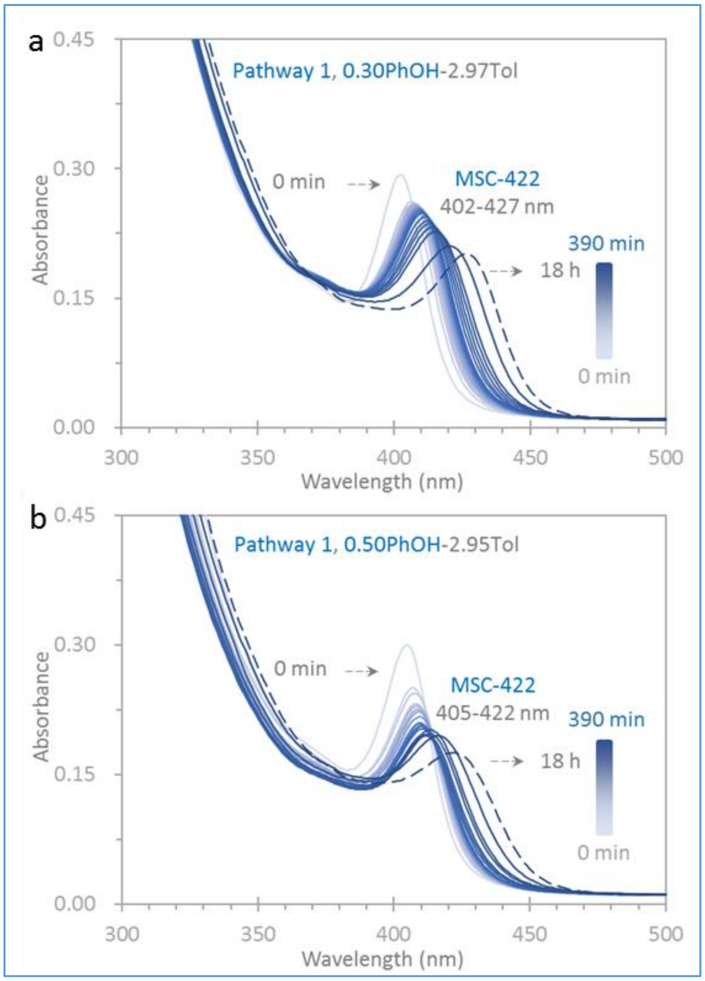

Figure S1-2. Pathway 1 monitored by optical absorption spectroscopy in situ and in real time for the MSC-399 to MSC-422 isomerization. The MSC-399 sample (120 μL) is dispersed in 3.00 mL mixture of Tol and PhOH, with the PhOH amount of 0.30 (a) and 0.50 mL (b). The spectra are collected at room temperature with the interval of 5 min from 0 to 60 min, 20 min from 80 to 120 min, at 150, 180, 390 min, and at 18 h.

For Dispersion a, the peak is at 402 nm at 0 min with an OD of 0.28. At 5 min, the peak redshifts to 405 nm and the strength decreases to 0.24. At 18 h, the peak is at 427 nm with an OD of 0.18.

For Dispersion b, MSC-399 peaks at 405 nm at 0 min with an OD of 0.28. At 5 min, the peak is at 409 nm with an OD of 0.23. At 18 h, the peak is at 422 nm with an OD of 0.16.

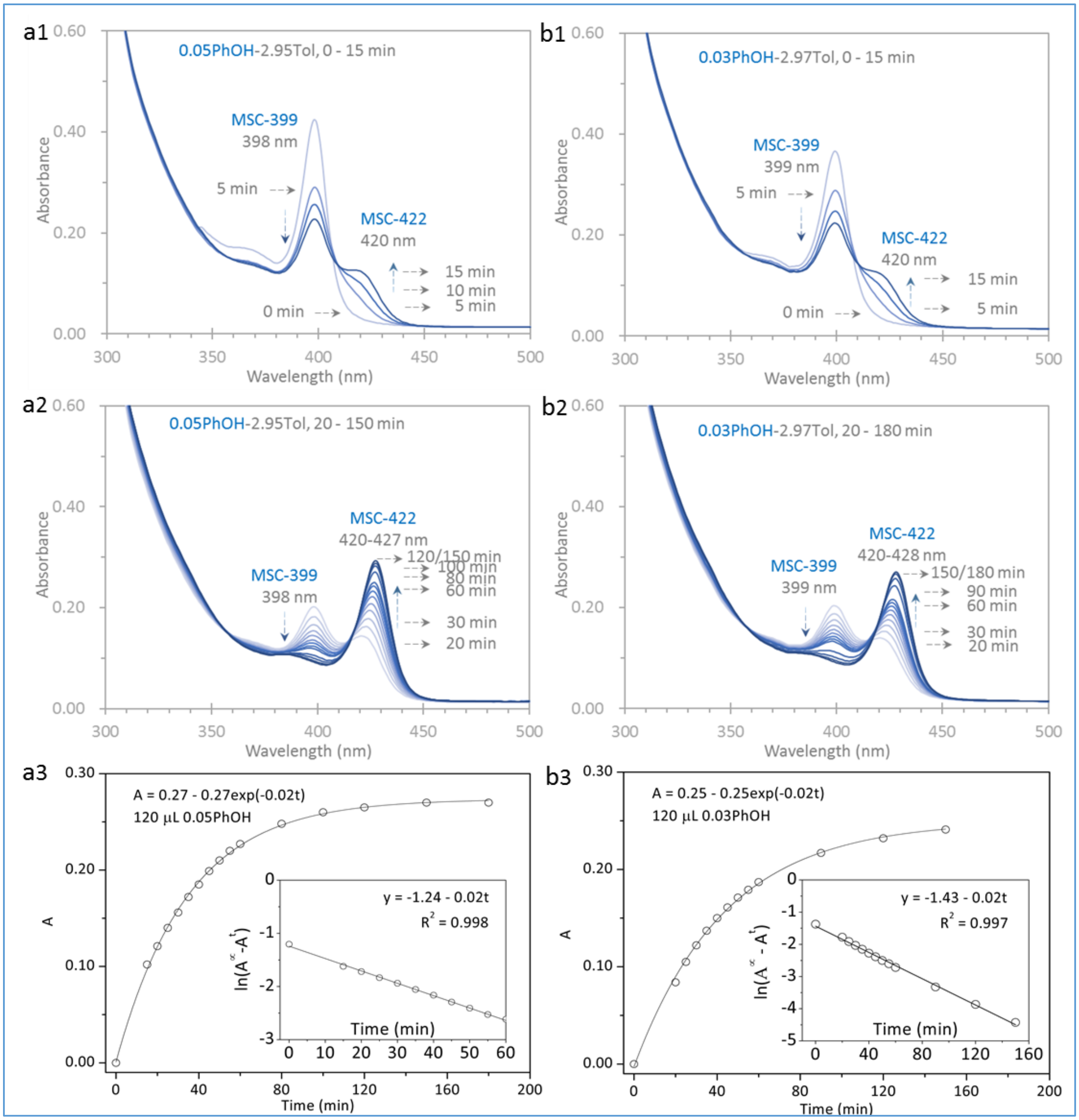

Figure S1-3. Isomerization in two dispersions with the first-order reaction kinetic fitting. The MSC-399 sample (120 µL) is dispersed in 3.00 mL mixture of Tol and PhOH, which contains 0.05 mL (a, left panel) and 0.03 mL (b, right panel) of PhOH. Dispersion a is that shown in Figure 1b. For Dispersion a the spectra are collected at room temperature with the interval of 5 min from 0 to 60 min, 20 min from 60 to 120 min, and at 150 min. For Dispersion b the spectra are collected at room temperature with the interval of 5 min from 0 to 60 min, and 30 min from 60

to 180 min. The net absorbance at 427 (a3) and 428 (b3) nm (denoted as A, open circles) is obtained by subtracting the absorbance at 0 min. The evolution of MSC-422, mainly after 20 min via Pathway 2, follows first-order reaction kinetics behavior with a rate constant of 0.02 min^{-1}. From the present study, it is understandable that the OD of MSC-399 at 0 min is larger in Dispersion a than that in Dispersion b, and the final OD of MSC-422 is larger in Dispersion a than that in Dispersion b.

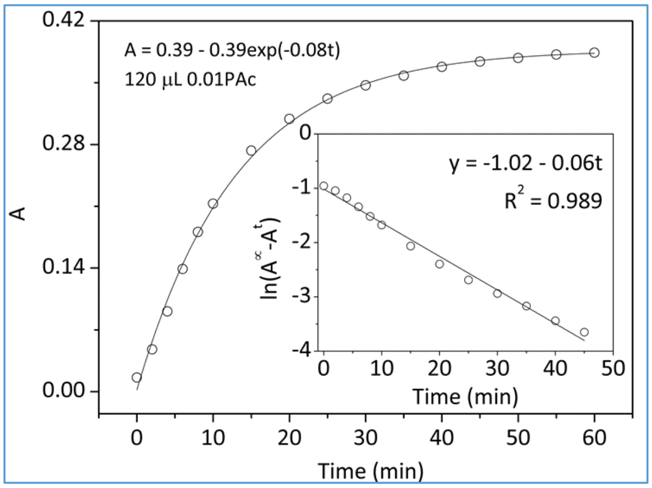

Figure S1-4. Kinetic study of the evolution of MSC-422 from the Figure 1f reaction. In this case Pathway 2 is followed with little contribution from Pathway 1. The net absorbance at 428 nm (denoted as A, open circles) is obtained by subtracting the absorbance at 0 min. The first-order reaction kinetics has the rate constant of 0.08 min^{-1}. Step 2c seems to proceed faster than that shown in Figure S1-3 with the incoming ligand of PhOH.

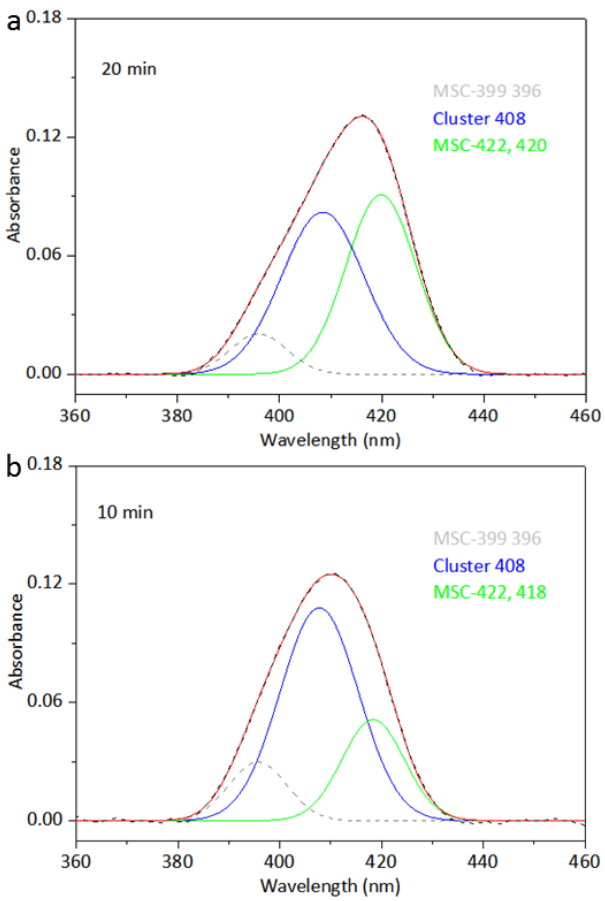

Figure S1-5. Deconvolution of the 10 and 20 min spectra in Figure 1a. The Gaussian peak fitting is done in a similar way as that for the spectra in Figure 5. The baseline subtraction is performed prior to the deconvolution that is carried out with a least-squares fitting (black dashed line). The dashed gray traces are for MSC-399, the blue traces for the Pathway 1 intermediate cluster (peaking at 408 nm), and the green traces for MSC-422. The red traces show the superimposed result of the three Gaussian peaks, which overlap with the dashed black traces.

Note S1. A detailed description for the NMR signal in Figure 2.

In Part a, the ^{13}C resonance signals of the d, b, c, and a carbon atoms of Trace 1 are respectively located at 156.72, 129.42, 119.88, and 115.51 ppm, while those of Trace 2 are respectively at 155.89, 129.38, 120.31, and 115.17 ppm. From Trace 2 to Trace 1, the signals of the d and a carbon atoms shift 0.83 (155.89 to 156.72) and 0.34 (115.17 to 115.51) ppm in the downfield direction, respectively.

In Part b, the ^{1}H resonance signals of the corresponding hydrogen atoms of the b, c, and a sites and of the −OH group in Trace 1 are respectively located at 7.10, 6.80, 6.74, and 4.73 ppm, while those in Trace 2 are respectively at 7.04, 6.77, 6.54, and 4.40 ppm.

In Part c, the ^{13}C resonance signals of the 1, 2, and 3 carbon atoms of OLA in Trace 1 are respectively located at 41.30, 32.46, and 26.73 ppm, while the 9 and 10 carbon atoms are at 129.95 and 129.77 ppm, respectively. Traces 3 and 4 are similar; the 1, 2, and 3 carbon atoms of Trace 3 are at 42.34, 34.04, and 27.02 ppm, respectively, while those of Trace 4 are at 42.35, 34.14, and 27.03 ppm, respectively. The 9 and 10 carbon atoms of Trace 3 are at 129.86 and 129.81 ppm, respectively, while those of Trace 4 at 129.86 and 129.82 ppm, respectively.

In Part d, the ^{1}H resonance signals of the corresponding hydrogen atoms of the 9/10, 1, 8/11, 2, 12-17/3-7, and 18 sites of Trace 1 are respectively located at 5.51, 2.24, 2.12, 1.42, 1.30, and 0.94 ppm. Those of Trace 3 are at 5.50, 2.55, 2.13, 1.41, 1.30, and 0.95 ppm, respectively, and of Trace 4 at 5.50, 2.53, 2.13, 1.41, 1.30, and 0.95 ppm, respectively. Again, Traces 3 and 4 look similar. The signals of the $-NH_2$ group in Traces 3 and 4 are at 0.85 and 0.64 ppm, respectively; the interaction between the $-NH_2$ group and the Cd atom results in a downfield shift from Trace 4 to Trace 3.

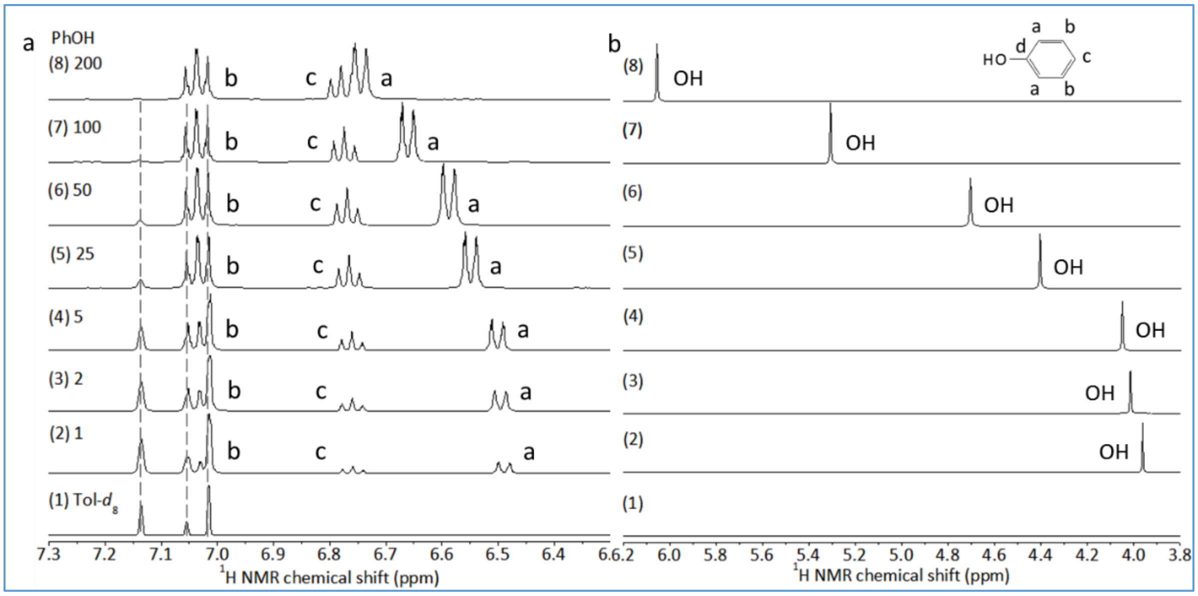

Figure S2-1. ^{1}H NMR of PhOH with various concentrations in Tol-d_8 at room temperature. Trace 1 is from Tol-d_8 (1.00 mL), with three signals located at 7.14, 7.06, and 7.02 ppm; the vertical dashed lines are guide for the eye. The samples are obtained by dispersing PhOH in Tol-d_8 with the total volume of 1.00 mL. Traces 2 to 8 are obtained with the PhOH volume of 1, 2, 5, 25, 50, 100, and 200 μL, respectively. (a) The signals of a-site hydrogen atoms are respectively located at 6.48, 6.49, 6.49, 6.54, 6.58, 6.65, and 6.73 ppm. (b) The signals of the −OH group are respectively located at 3.96, 4.02, 4.05, 4.40, 4.70, 5.31, and 6.06 ppm. In a side note, the sample shown in Figure 1a has the PhOH volume of 0.10 mL in 2.90 mL of Tol. For the hydrogen atoms of Site a and of the −OH group, it is evident that the PhOH concentration affects their signals; the larger the PhOH amount is, the more the two signals downfield shift. For the hydrogen atoms of Sites b and c, the concentration effect is not obvious. The concentration effect supports the assignment of the signal obtained.

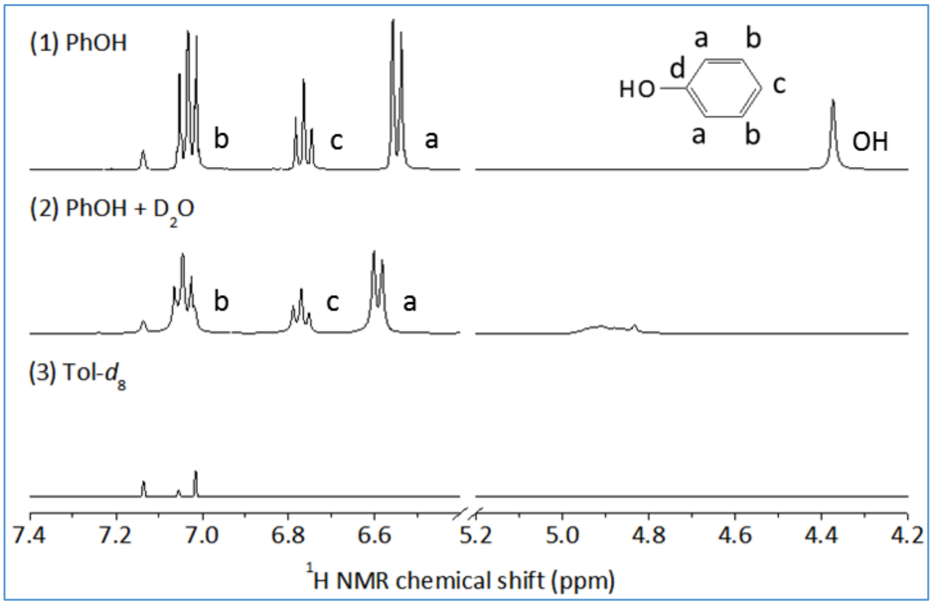

Figure S2-2. ^{1}H NMR study to support the assignment of the -OH group of PhOH. Trace 1 is from PhOH (20 μL) in Tol-d_8 (580 μL); the PhOH concentration is similar to that of Figure 1a. The signal of the −OH group is located at 4.33 ppm. After a drop of D_2O addition with shaking (Trace 2), this signal disappears. Trace 3 is Trace 1 in Figure S2-1. The disappearance of the 4.33 ppm signal in Trace 2 is in agreement with its assignment to the −OH group. The pK_a values of water, -OH of PhOH, and -C_6H_5 of PhOH are 14, 10,[20] and 43, respectively. Thus, the CH bond cannot dissociate to exchange with D_2O, while the OH bond can with the reaction of Ph-OH + D_2O ⇔ Ph-OD + DOH.

(20) Shubina, E. S.; Belkova, N. V.; Krylov, A. N.; Vorontsov, E. V.; Epstein, L. M.; Gusev, D. G.; Niedermann, M.; Berke, H. S. Evidence for intermolecular M-H···H-OR hydrogen bonding: interaction of $WH(CO)_2(NO)L_2$ hydrides with acidic alcohols. *J. Am. Chem. Soc.* **1996**, *118*, 1105-1112.

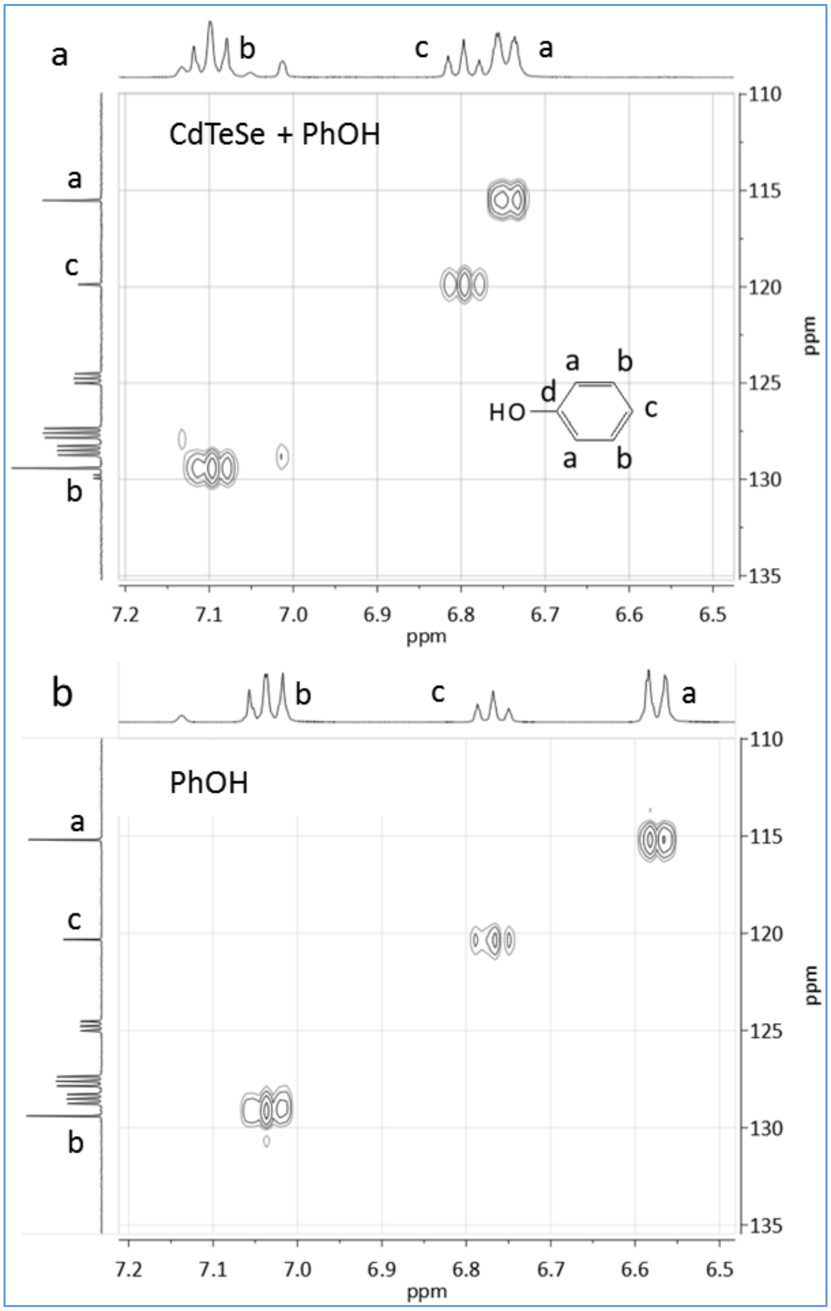

Figure S2-3. ^{1}H-^{13}C heteronuclear single quantum correlation (HSQC) NMR. (a) The spectrum is from the mixture of PhOH (20 μL), the MSC-399 sample (24 μL), and Tol-d_8 (580 μL) (which is similar to that shown in Figure 1a). (b) The spectrum is from a mixture of PhOH (20 μL) and Tol-d_8 (580 μL). The HSQC NMR supports our assignments for the a- to c-site atoms.

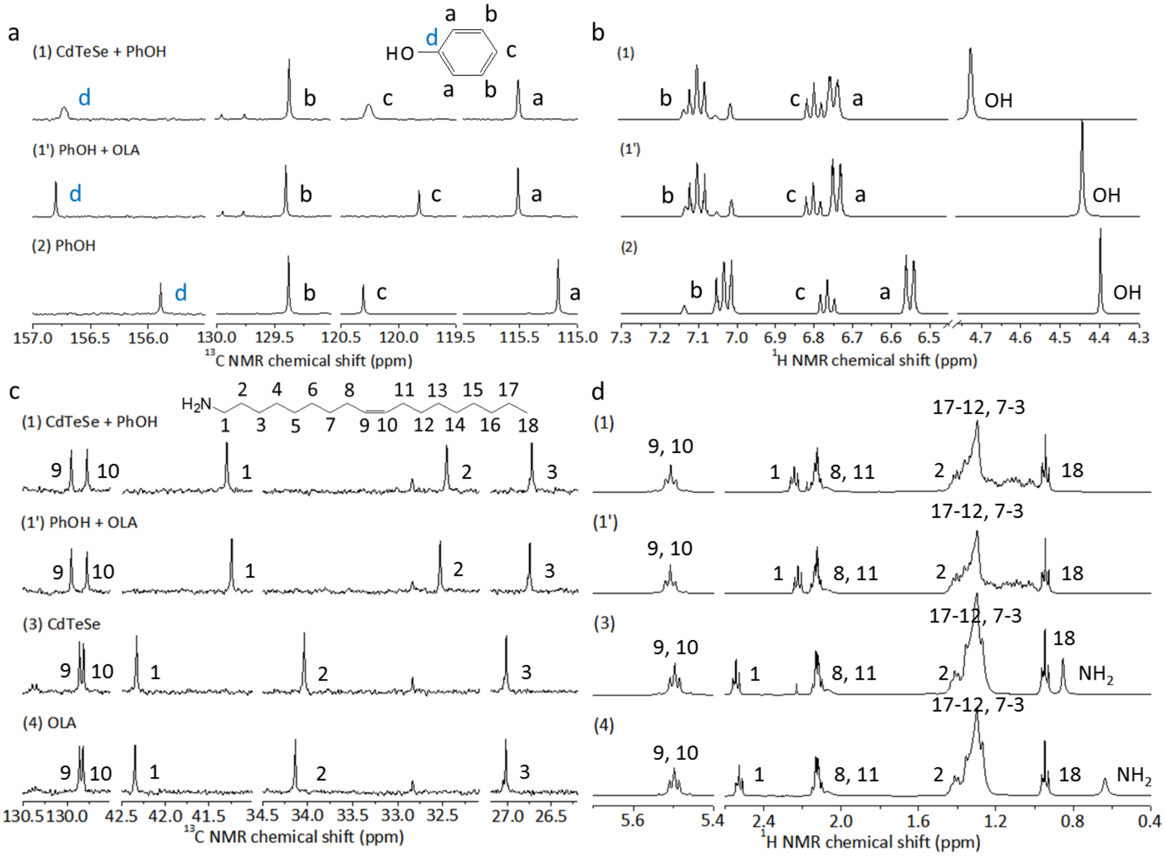

Figure S2-4. ^{13}C and ^{1}H NMR study in Tol-d_8 at room temperature. The presentation format is similar to that of Fig. 2, and Traces 1 to 4 are shown in Fig. 2. Trace 1' is a mixture of OLA (20 μL) and PhOH (20 μL) in Tol-d_8 (580 μL). It is evident that at room temperature in Tol, OTA interacts with PhOH. The shift of the resonance signals is in agreement with the ligand exchange reaction proposed in Eq. (1a).

In Parts a and b, the d signal broadens in Trace 1 and not in Trace 1'. The a to c signals are similar.

In Parts c and d, Traces 1 and 1' look similar, which is in agreement with the interaction between PhOH and the MSC-399 sample.

Note S2. Description of the spectrum change of the Figure 3 dispersions.

For Dispersions a and b, at 0 min, MSC-399 has an OD of 0.45 (a) and 0.47 (b), and a FWHM of 14 nm (a and b). At 5 min, MSC-399 decreases in strength by about 64% (a) and 66% (b), with an OD of 0.16 (a and b). At 10 min, the decreases in strength are about 80% (a) and 81% (b), with an OD of 0.09 (a and b). At 15 min, the strength decrease reaches about 87% (a and b) with an OD of 0.06 (a and b).

Upon the addition of 0.10 mL of PhOH (c), a broad peak at ~380 nm appears at 0 min. At 5 min, this peak redshifts to 390 nm with a slight decrease in strength. At 10 min, the 390 nm peak strength decreases somewhat more, and another peak at 418 nm is seen indicating the probable evolution of MSC-422. Afterwards, MSC-422 increases; at 80 min, MSC-422 peaks at 422 nm and reaches its maximum strength with an OD of 0.18 and a FWHM of 31 nm. From 80 to 120 min, little further change occurs.

Upon the addition of 0.05 mL of PhOH (d), a broad peak at ~390 nm is also present at 0 min. At 5 min, this peak seems to be declining, while a red-side peak at 418 nm evolves, suggesting the development of MSC-422. Afterwards, MSC-422 increases. At 100 min, MSC-422 reaches a maximum strength with an OD of 0.21 and a FWHM of 23 nm while peaking 419 nm. For times up to 120 min, there is little change to this peak.

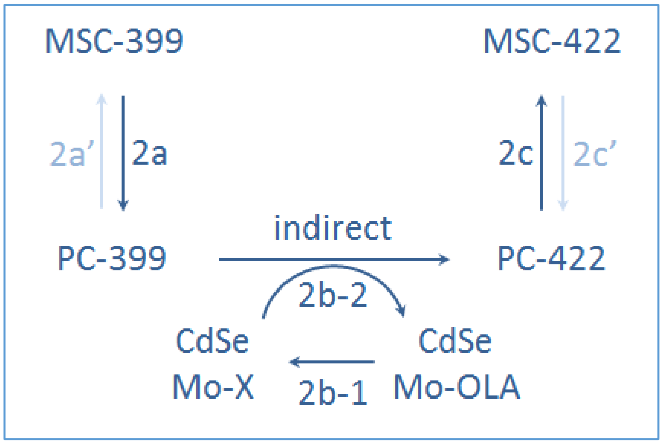

Figure S3-1. Illustration of the experimental result shown in Figure 3. When CdTeSe MSC-399 is dispersed in Tol, MSC-399 isomerizes to its counterpart, non-absorbing CdTeSe PC-399 (Step 2a). When an incoming ligand X (such as PhOH or PAc) is added, CdTeSe PC-399 transforms mainly to CdTeSe PC-422; this reaction requires Steps 2b-1 and 2b-2. Step 2b-1 requires the presence of an incoming ligand X, while a relatively large amount of the ligand halts the evolution of CdTeSe MSC-422 from CdTeSe PC-422 (Step 2c). When the OLA number is n for MSC/PC-399, it seems reasonable that MSC/PC-422 has an OLA number of n-1 and a X number of 1. The equations for Pathways 1 and 2 are accordingly presented below.

CdTeSe MSC-399-OLA_n + X $\rightarrow$ CdTeSe MSC-399-OLA_{n-1}/X + OLA (1a)

CdTeSe MSC-399-OLA_{n-1}/X $\rightarrow$ CdTeSe MSC-422-OLA_{n-1}/X (1b)

CdTeSe MSC-399-OLA_n $\rightarrow$ CdTeSe PC-399-OLA_n (2a)

CdSe Mo/Fr-OLA + X $\rightarrow$ CdSe Mo/Fr-X + OLA (2b-1)

CdTeSe PC-399-OLA_n + CdSe Mo/Fr-X $\rightarrow$

CdTeSe PC-422-OLA_{n-1}/X + CdSe Mo/Fr-OLA (2b-2)

CdTeSe PC-422-OLA_{n-1}/X $\rightarrow$ CdTeSe MSC-422-OLA_{n-1}/X (2c)

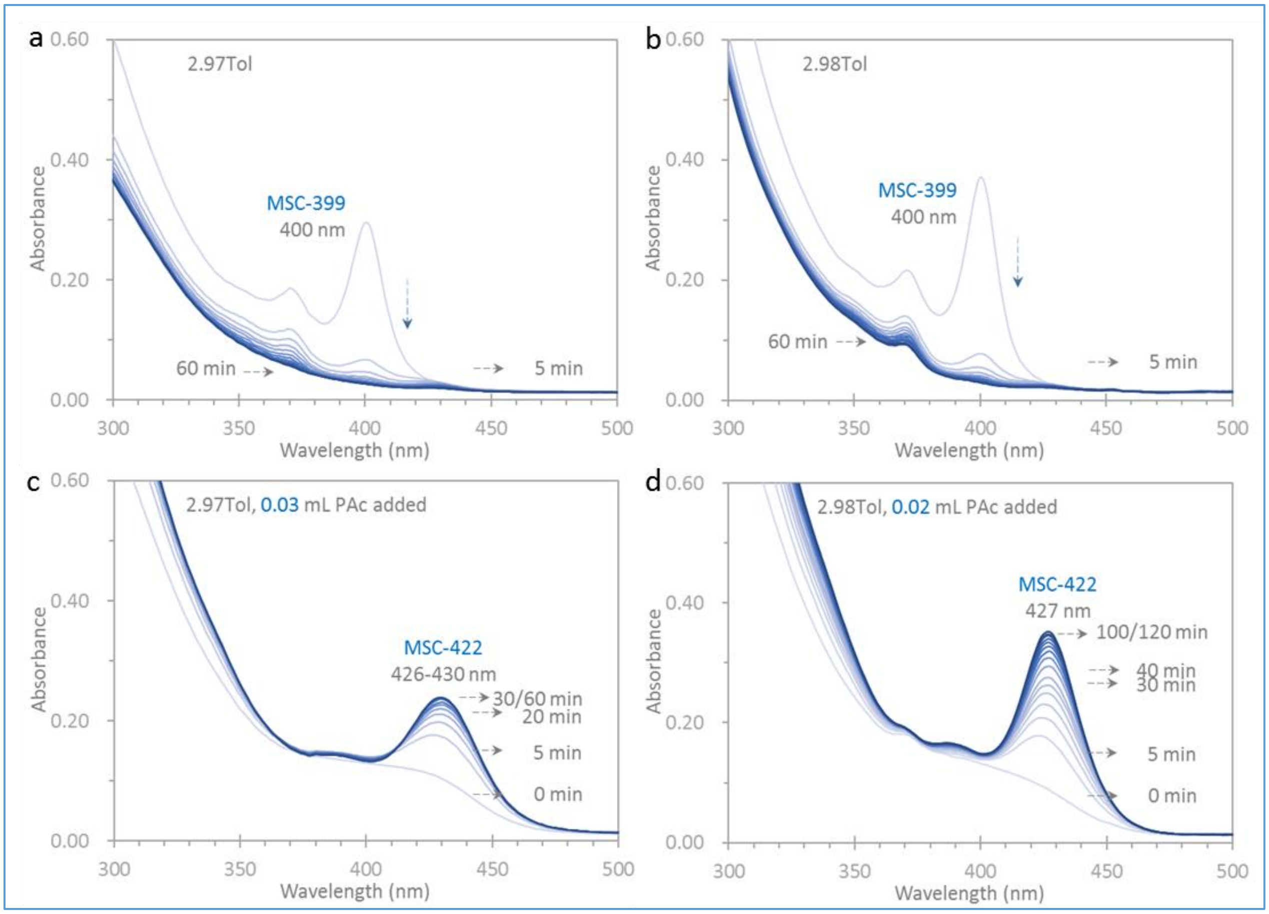

Figure S3-2. Evolution of CdTeSe MSC-422 induced by PAc addition monitored by in situ and real time optical absorption spectra spectroscopy. The MSC-399 sample (120 µL, about five days stored in liquid nitrogen prior to use) is dispersed in 2.97 mL (a) and 2.98 mL (b) of Tol. From each dispersion, 13 spectra are collected from 0 to 60 min with the interval of 5 min. MSC-399 transforms to PC-399 via Step 2a, and almost disappears at 30 min. After the collection at the 60 min point, 0.03 mL (c) and 0.02 mL (d) of PAc is added, respectively. The concentrations of MSC-399 and PAc are similar to those shown in Figures 1d and 1e, respectively. For Dispersion c, the spectra are collected at room temperature with the interval of 5 min from 0 to 60 min. For Dispersion d, the spectra are collected at room temperature with the interval of 5 min from 0 to 30 min, and 10 min from 30 to 120 min. At 0 min, a peak is at ~425 nm (c) and 420 nm (d), indicating the evolution of MSC-422. At 5 min, a peak is 426 nm (c) and 424 nm (d). At 30 min, MSC-422 in Dispersion c reaches its maximum strength with an OD of 0.22 and a FWHM of 36 nm. At 100 min, MSC-422 in Dispersion d reaches its maximum strength with an OD of 0.33 and a FWHM of 30 nm. MSC-422 displays a smaller OD and a larger FWHM in Dispersion c. PAc is required for Step 2b but inhibits Step 2c. We note that the incoming ligand does not facilitate the PC to MSC isomerization in Tol. It may be helpful to consider that the forward is (salt) crystallization and the backward is (salt) dissolution. The presence of a ligand increases the environment polarity, the consequence of which is similar to what results from a temperature increase.

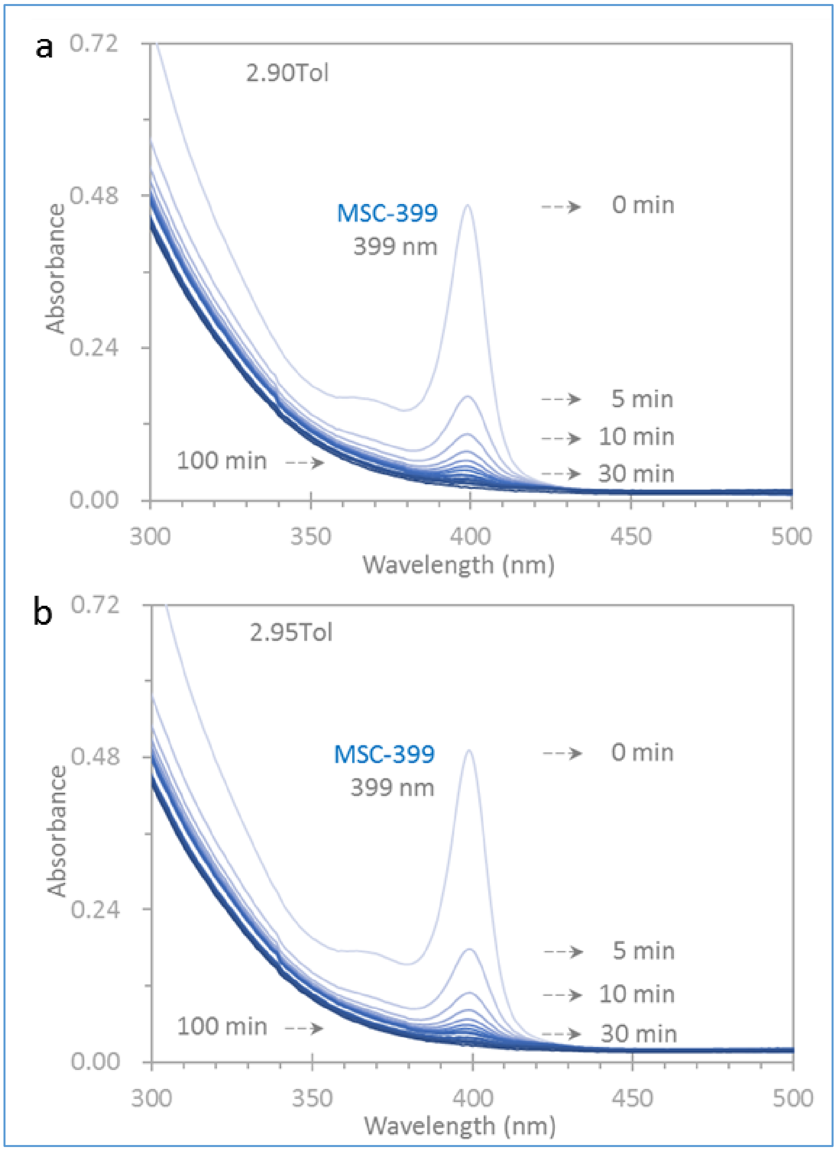

Figure S3-3. The full spectra collected for Parts a and b in the top panel of Figure 3. From each dispersion, 12 spectra are collected with the interval of 5 min from 0 to 30 min, 10 min from 30 to 60 min, and 20 min from 60 to 100 min. MSC-399 disappears completely around 80 min.

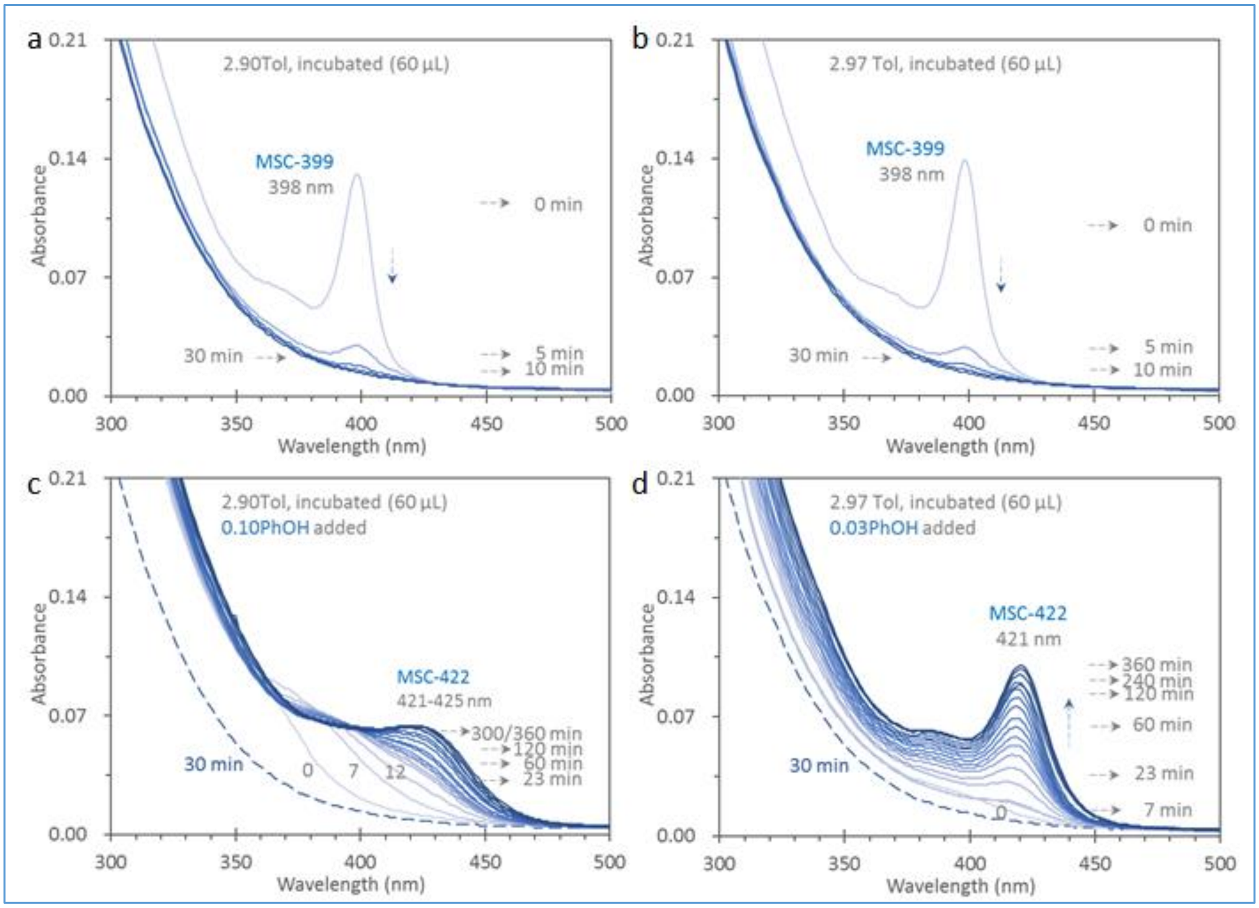

Figure S3-4. Evolution of CdTeSe MSC-422 induced by PhOH addition monitored by in situ and real time optical absorption spectra spectroscopy. The MSC-399 sample (60 μL) is dispersed in 2.90 (a) and 2.97 (b) mL of Tol. The spectra are collected at 0, 5, 10, 20, and 30 min. MSC-399 disappears completely at 20 min. After the spectra collection at the 30 min point, 0.10 (c) and 0.03 (d) mL of PhOH is added, respectively. The spectra are collected at 0, 7, 12, 17, 23, 28, 34 min, with the interval of 10 min from 40 to 60 min, 20 min from 60 to 120 min, 30 min from 120 to 180 min, and with 60 min from 180 to 360 min. The dashed traces in Parts c and d are the 30 min spectra in Parts a and b, respectively. For Dispersion c at 300 min, MSC-422 peaks at 425 nm and reaches its maximum strength with an OD of 0.05 and a FWHM of 40 nm. For Dispersion d at 360 min, MSC-422 peaks at 421 nm and reaches its maximum strength with an OD of 0.92 and a FWHM of 26 nm. MSC-422 in Dispersion c has a smaller strength and a larger FWHM than in Dispersion d. PhOH is required for Step 2b but suppresses Step 2c.

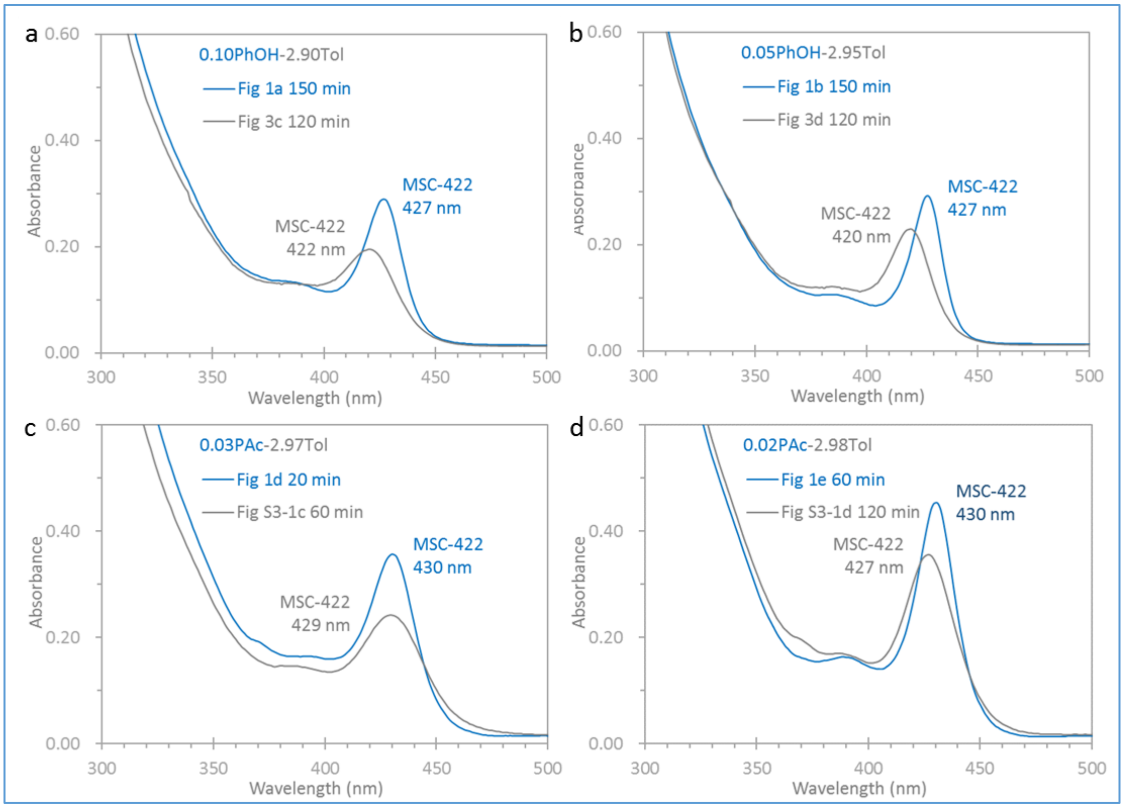

Figure S3-5. Comparison of Figure 1 and Figure 3 approaches to MSC-422. The Figure 1 approach (blue traces) to MSC-422 is placing the MSC-399 sample in a mixture of PhOH/PAc and Tol. The Figure 3 approach (gray traces) to MSC-422 is placing the MSC-399 sample in Tol, followed by the PhOH/PAc addition after the complete disappearance of MSC-399. MSC-422 via the Figure 1 approach has a larger OD and a narrower FWHM than that of the Figure 3 approach. The Figure 3 approach is more suitable for the investigation of the transformation pathway.

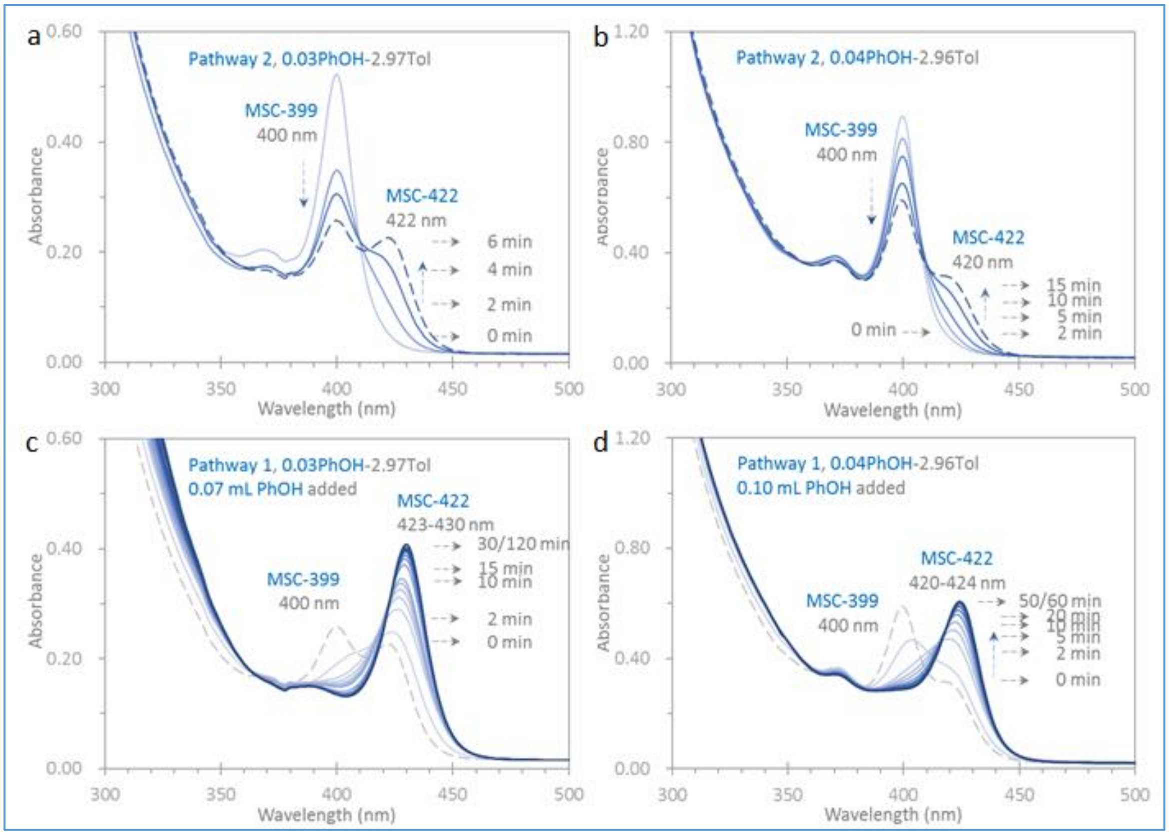

Figure S4-1. Activation of Pathway 1 via extra PhOH addition monitored by in situ and in real time optical absorption spectroscopy. The MSC-399 sample (120 µL) is dispersed in the mixture (3.00 mL) of Tol and PhOH (0.03 mL (a) or 0.04 mL (b)). Pathway 2 is followed. After the collection (dashed traces) at the 6 min (a) or 15 min (b) point, 0.07 (c) and 0.10 (d) mL PhOH is added, respectively. Pathway 1 is activated upon the addition of extra PhOH.

For Dispersion a, it is similar to that shown in Figure S1-3b, with regard to the amounts of MSC-399 (120 µL) and PhOH (0.03 mL). Four spectra are collected with the interval of 2 min from 0 to 6 min. At 0 min, MSC-399 has an OD of 0.51 and a FWHM of 16 nm. At 2 min, MSC-399 decreases in strength by about 34% with an OD of 0.32. A red side bump evolves. At 4 min, the strength of MSC-399 decreases by about 43% with an OD of 0.29; the bump develops into a peak at 420 nm with an OD of 0.17. At 6 min, MSC-399 decreases in strength by about 52% with an OD of 0.24, while MSC-422 peaks at 422 nm with an OD of 0.21 and a FWHM of 24 nm.

For Dispersion b, five spectra are collected at 0, 2, 5, 10, and 15 min. At 0 min, MSC-399 has an OD of 0.87 and a FWHM of 15 nm. The strength of MSC-399 keeps decreasing. At 10 min, the strength of MSC-399 decreases ~27% with an OD of 0.63; MSC-422 evolves. At 15 min, MSC-399 has an OD of 0.56, and MSC-422 has an OD of 0.26 and a FWHM of 22 nm.

For Dispersion c, it has a total PhOH amount of 0.10 mL, similar to that shown in Figure 1a. 19 spectra are collected with the interval of 2 min from 0 to 10 min, 5 min from 10 to 60 min, and 20 min from 60 to 120 min. At 0 min, a bump is around 405 nm, and MSC-422 peaks at 424 nm with an OD of 0.23. At 2 min, the bump disappears, and MSC-422 redshifts to 426 nm with an OD of 0.27. Afterwards, MSC-422 keeps increasing, and reaches its maximum strength at 30 min peaking at 430 nm with an OD of 0.37 and a FWHM of 21 nm. There is little change up to 120 min.

For Dispersion d, nine spectra are collected, at 0, 2, and 5 min, and with the 10 min interval from 10 to 60 min. At 0 min, an absorption peaks at 403 nm with OD of 0.44, together with MSC-422 at 422 nm with an OD of 0.30. At 2 min, the 403 nm peak disappears; MSC-422 increases with an OD of 0.42. The strength of MSC-422 keeps increasing, reaching the maximum at 50 min with an OD of 0.57 and a FWHM of 21 nm. From 50 to 60 min, little change is observed.

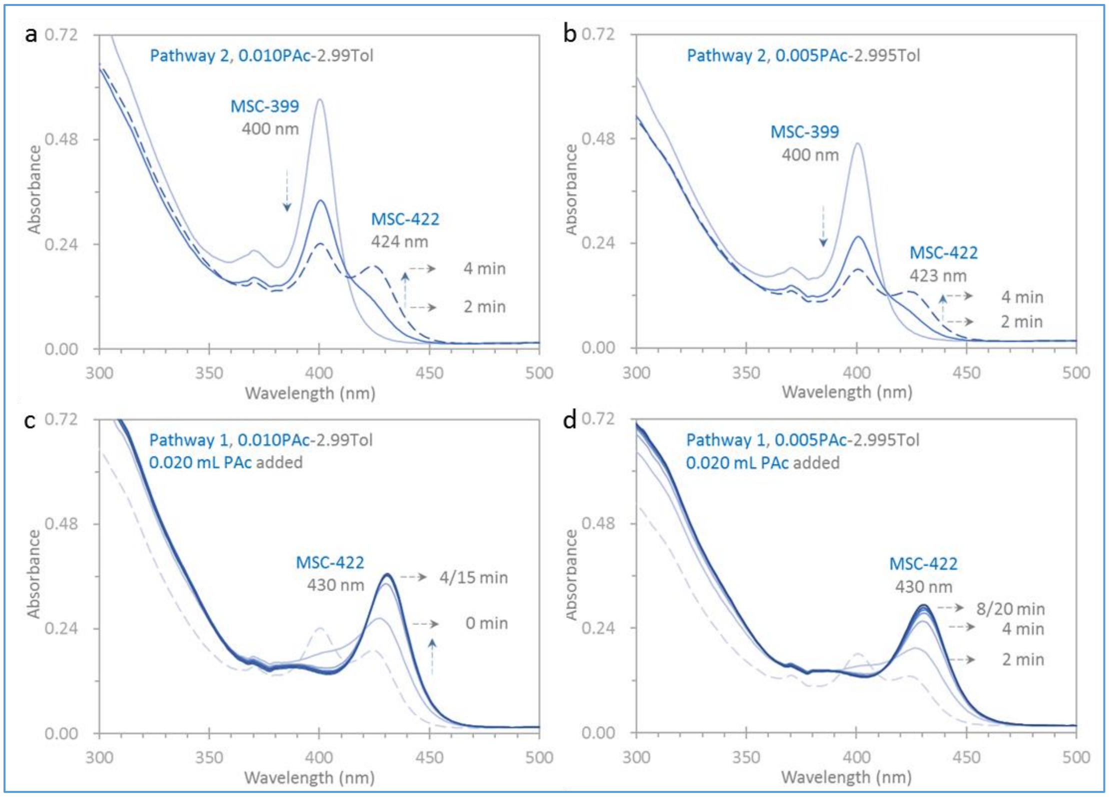

Figure S4-2. Activation of Pathway 1 via extra PAc addition monitored by in situ and in real time optical absorption spectroscopy. The MSC-399 sample (120 μL) is dispersed in the mixture (3.00 mL) of Tol and PAc (0.010 mL (a) or 0.005 mL (b)). The spectra are collected from 0 to 4 min with the interval of 2 min. At 0 min, MSC-399 peaks at 400 nm with an OD of 0.56 (a) and 0.45 (b), a FWHM of 16 nm (a and b). At 2 min, MSC-399 decreases ~41% with an OD of 0.33 (a) and ~47% with an OD of 0.24 (b); in the meantime, MSC-422 evolves. At 4 min, the strength of MSC-399 decreases 59% and 64% with an OD of 0.23 (a) and 0.16 (b), respectively. MSC-422 peaks at 424 (a) and 423 (b) nm with an OD of 0.18 (a) and 0.11 (b). After the collection at 4 min, 0.020 mL PAc is added, the resulting Dispersion c is similar to that shown in Figure 1d with the PAc amount of 0.03 mL. For Dispersion c, the spectra are collected with the interval of 2 min from 0 to 10 min, and at 15 min. For Dispersion d, the spectra are collected with the interval of 2 min from 0 to 8 min, and 5 min from 10 to 20 min. Upon the addition of extra PAc at 0 min, MSC-399 almost disappears; MSC-422 peaks at 427 (c) and 426 (d) nm with an OD of 0.24 (c) and 0.17 (d). Pathway 1 is activated up the addition of extra PAc.

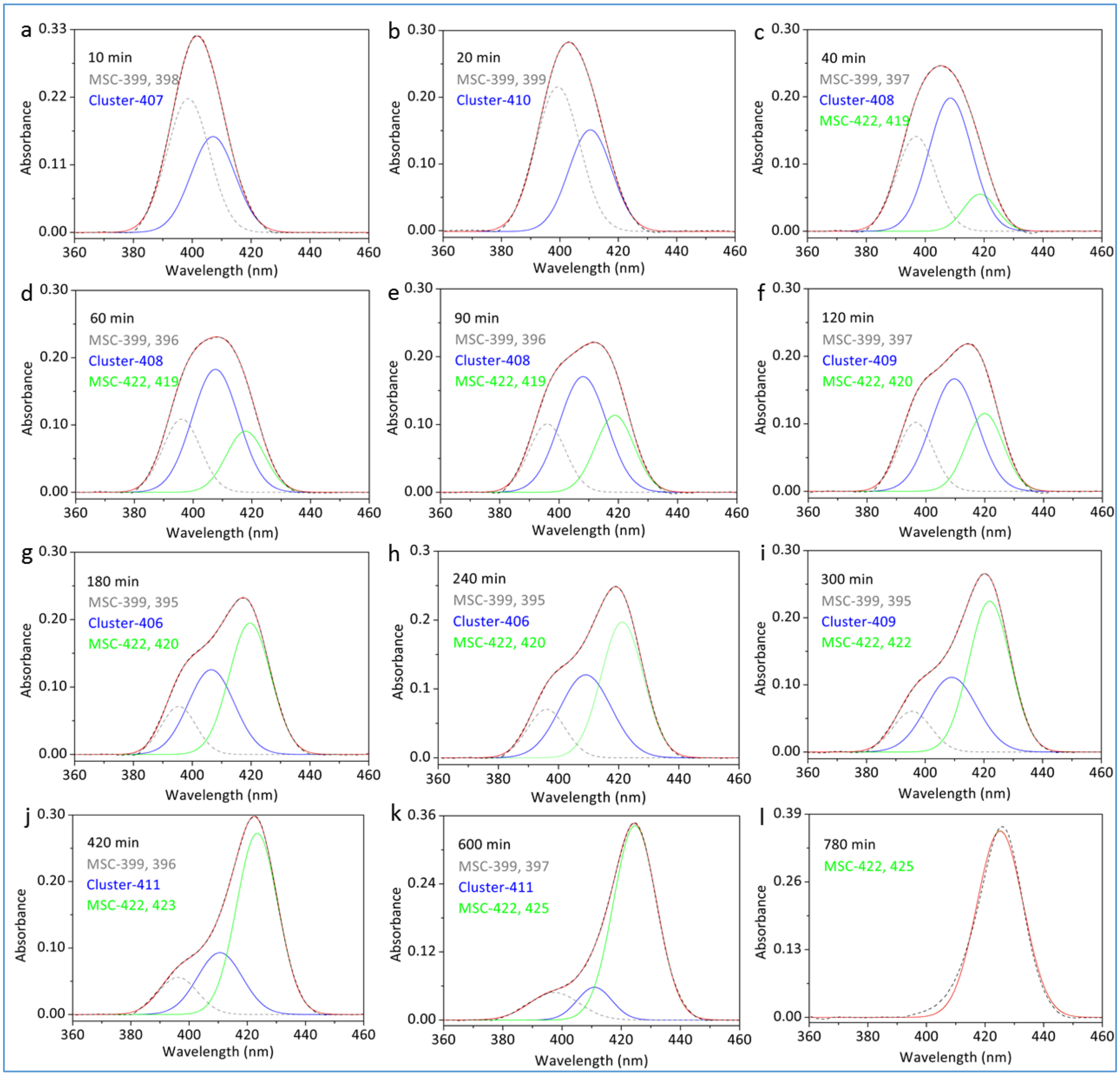

Figure S5-1. Deconvolution of some representative spectra shown in Figure 5a. The baseline subtraction is performed prior to the deconvolution that is carried out with a least-squares fitting (black dashed line). The dashed gray traces are for MSC-399, the blue traces for the Pathway 1 intermediate cluster, and the green traces for MSC-422. The red traces show the superimposed result of the one, two, or three Gaussian peaks, which overlap with the dashed black traces. From 0 to 35 min according to the deconvolution, MSC-399 and the intermediate clusters exist without MSC-422; MSC-399 decreases while the intermediate cluster increases. At

40 min, MSC-422 evolves and Pathway 2 is followed; MSC-422 increases. MSC-399 and the intermediate cluster disappear completely after 600 min, and only MSC-422 exists.

For the spectrum collected at 10 min, the peak is at 401 nm with an OD of 0.45 and a FWHM of 26 nm. At 20 min, the peak is still at 401 nm with an OD of 0.41 and a FWHM of 32 nm. At 30 min, the peak is at 402 nm with an OD of 0.39 and a FWHM of 35 nm. At 40 min, the absorption peaks at 403 nm with an OD of 0.36 and a FWHM of 36 nm, and does not change much up to 60 min. At 90 min, the absorption peaks at 403 nm, together with a red side peak at 412 nm; the two peaks have an OD of 0.33. At 120 min, the MSC-399 peak has an OD of 0.32, and the MSC-422 peak becomes more apparent with an OD of 0.33 and a FWHM of 25 nm. MSC-399 peaking at 400 nm keeps decreasing in strength, while MSC-422 keeps increasing. From 660 to 780 min, only MSC-422 exists with an OD increasing from 0.43 to 0.45, and a FWHM of 20 nm.

Careful observation is required for the two sets of spectra shown in Figures 1a and 5a. The two dispersions start with MSC-399 and end with MSC-422. Our study with the fitting analysis shows that the former at room temperature follows Pathway 1, while the latter at 7 °C follows Pathway 1 at first and then Pathway 2.

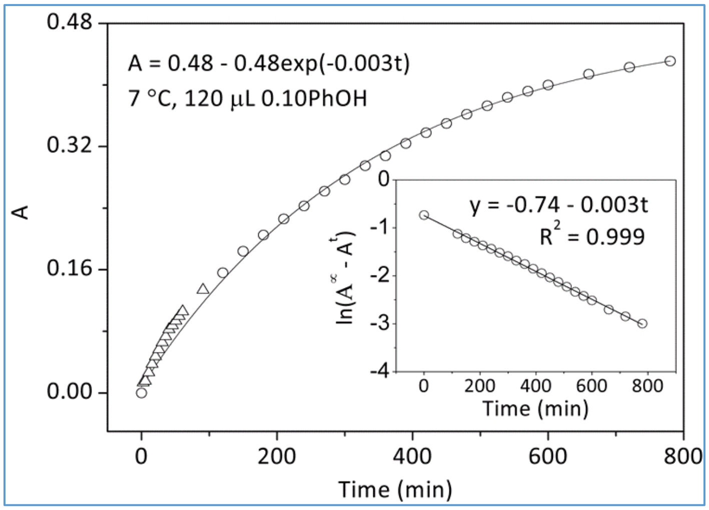

Figure S5-2. Kinetic study for the evolution of MSC-422 shown in Figure 5a. The net absorbance at 425 nm (denoted as A, open circles for those after 120 min) is obtained by subtracting the absorbance at 0 min. After 120 min, the evolution of MSC-422 displays first-order reaction kinetics behavior with a small rate constant of 0.003 min^{-1}. Pathway 2 is followed at 7 °C in the later stage; the open circle at (0,0) is assumed for the fitting. For the similar dispersion at room temperature shown in Figure 1a, Pathway 1 is followed. We argue that the temperature is not beneficial to the PC to MSC isomerization in Tol, as the forward is similar to (salt) crystallization and the backward to (salt) dissolution. At 7 °C, the PC-422 to MSC-422 (Step 2c) is relatively more favored, compared to that occurs at room temperature.

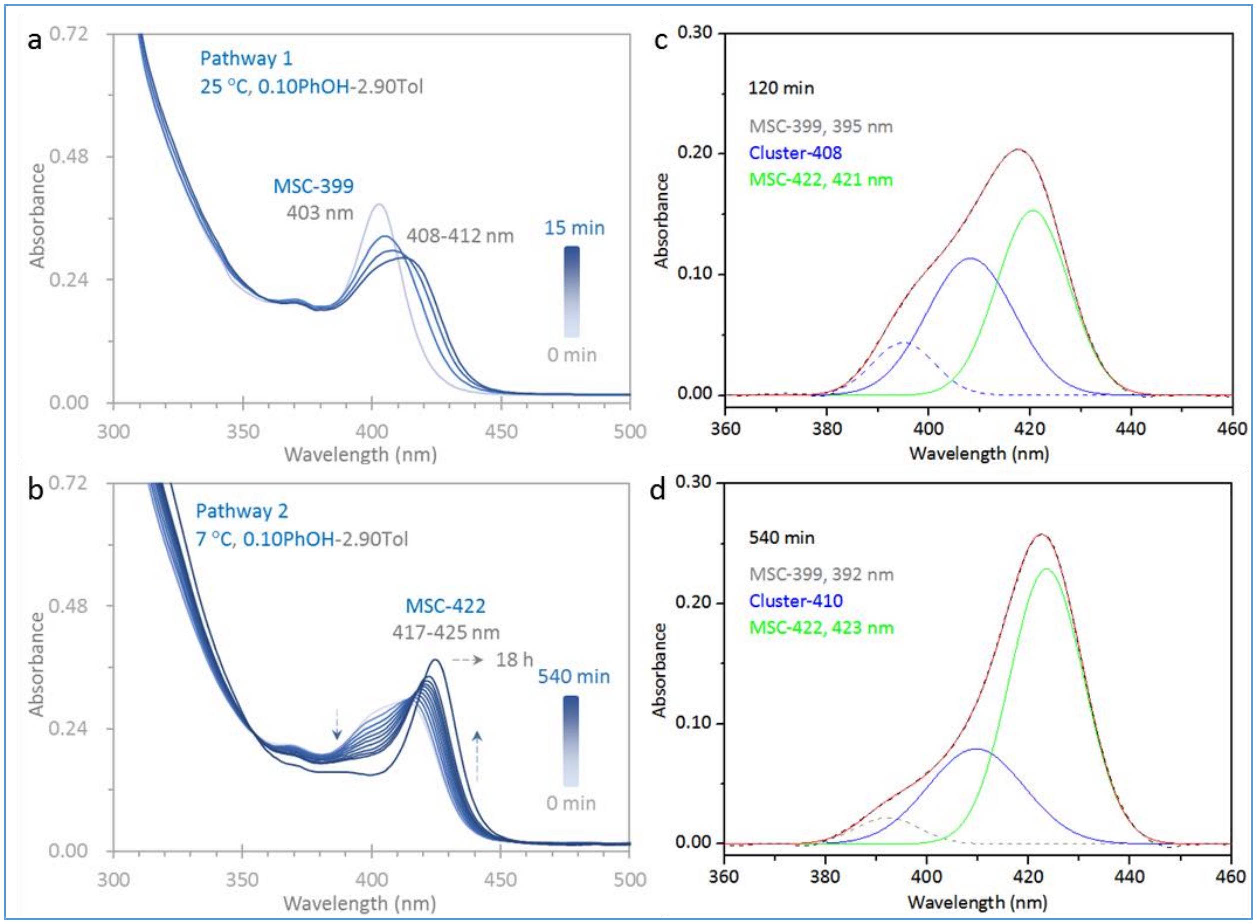

Figure S5-3. A dispersion at 25 °C first and then at 7 °C. The dispersion is similar to that shown in Figure 1a. The MSC-399 sample (120 μL) is dispersed in the mixture of 2.90 mL Tol and 0.10 mL PhOH at 25 (a); Pathway 1 is followed at room temperature. After 15 min, the dispersion is placed at 7 °C (b). Deconvolution is performed in a similar fashion for the 120 (c) and 540 (d) min spectra in Part b. At 7 °C, the remaining MSC-399 isomerizes to MSC-422 via Pathway 2.

(a) Four spectra are collected at 25 °C with the interval of 5 min from 0 to 15 min. At 0 min, the peak is at 403 nm with an OD of 0.34 and a FWHM of 22 nm. At 15 min, the peak is at 412 nm with an OD of 0.27 and a FWHM of 30 nm.

(b) After the dispersion is placed at 7 °C, 11 spectra are collected, with the interval of 60 min from 0 to 540 min, and at 18 h. At 540 min, MSC-422 peaks at 422 nm with an OD of 0.27 and a FWHM of 21 nm. At 18 h, MSC-422 peaks at 425 nm with an OD of 0.36 and a FWHM of 20 nm.

(c) and (d) The deconvolution suggests the presence of MSC-399, the intermediate cluster, and MSC-422.

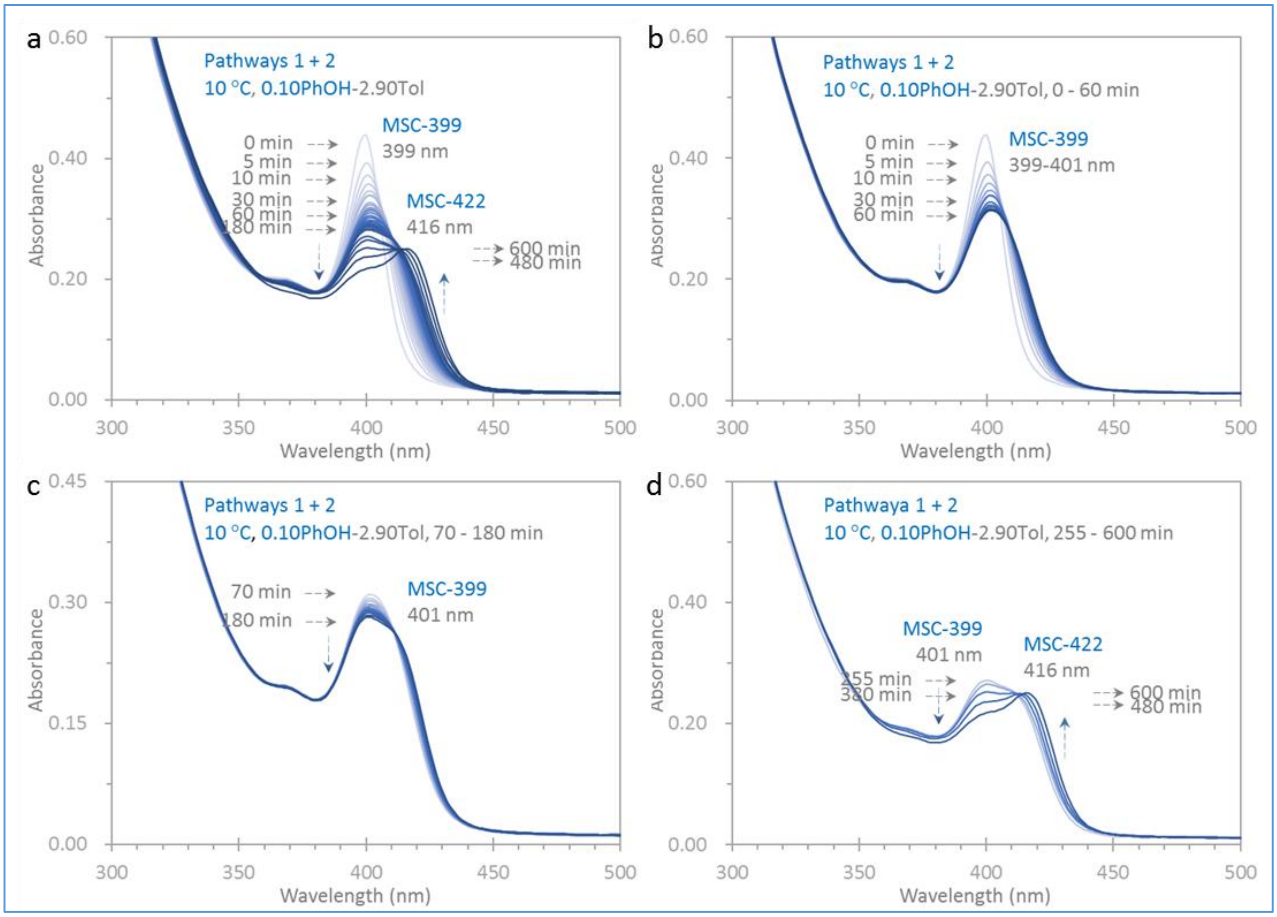

Figure S5-4. Optical absorption spectroscopy study of one dispersion at 10 °C. The MSC-399 sample (120 μL) is dispersed in the mixture of Tol (2.90 mL) and PhOH (0.10 mL). (a) A full set of spectra is obtained with the interval of 5 min from 0 to 60 min, 10 min from 60 to 180 min, at 255, 310, 380, 490 min, and 600 min. The spectra are highlighted from 0 to 60 min (b, 13 traces), 70 to 180 min (c, 12 traces), and from 255 to 600 min (d, five traces). Pathway 2 is also activated at 10 °C.

(b) From 0 to 60 min, MSC-399 keeps decreasing with broadening in line width and slight redshift in peak position. At 0 min, MSC-399 peaks at 399 nm with an OD of 0.42 and a FWHM of 18 nm. At 5 min, the peak strength is 0.38 with a FWHM of 24 nm. At 60 min, the peak is at 401 nm with an OD of 0.30 and a FWHM of 30 nm.

(c) From 70 to 180 min, the spectrum change is not much. At 70 min, the peak is at 401 nm with a broader FWHM of 34 nm and a shoulder in the red side. The shoulder indicates the presence of MSC-422.

(d) From 255 to 600 min, the shoulder peak increases and redshifts. At 255 min, the peak is at 416 nm.

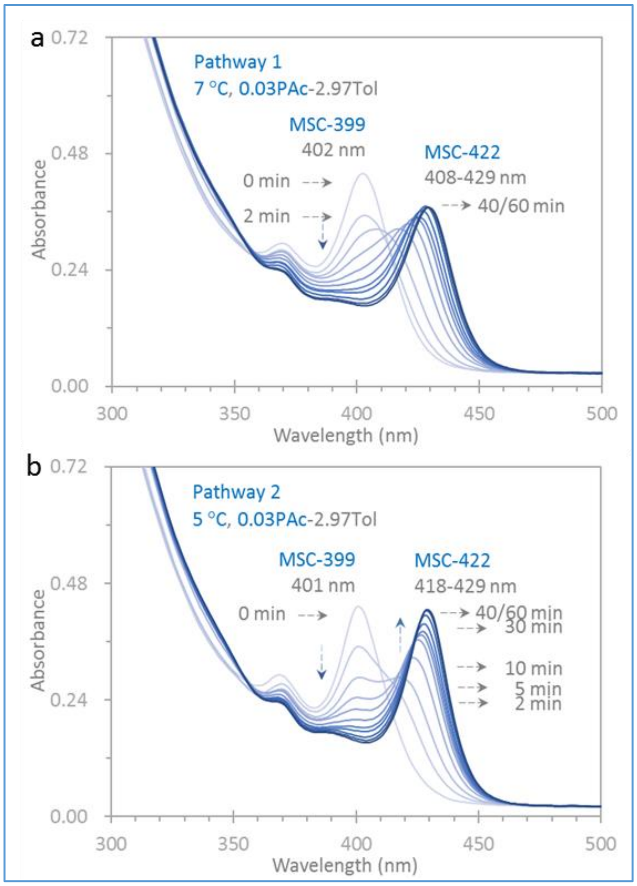

Figure S5-5. Optical absorption spectroscopy study of two PAc-containing dispersions at 7 and 5 °C. The MSC-399 sample (120 μL, after six-day storage in liquid nitrogen) is dispersed in the mixture of Tol (2.97 mL) and PAc (0.03 mL) at 7 °C (a) and 5 °C (b). 14 spectra are collected for each dispersion (that is similar to that shown in Figure 1d), which are at 0, 2 min, then with the interval of 5 min from 5 to 60 min. The Figure 1d peak at 0 min has an OD of 0.39 and a FWHM of 36 nm.

For Dispersion a at 0 min, a peak is at 402 with an OD of 0.41 and a FWHM of 28 nm. At 2 min, the peak is at 403 nm with an OD of 0.32 and a FWHM of 36 nm. At 5 min, this peak is at 408 nm with an OD of 0.29 and FWHM of 37 nm. At 40 min, the peak has an OD of 0.33 and a FWHM of 25 nm.

For Dispersion b at 0 min, a peak is at 401 nm with an OD of 0.41 and a FWHM of 26 nm. At 2 min, the peak is at 401 nm with an OD of 0.33, with a bump seen at the red side indicating the presence of MSC-422. At 5 min, the peak has an OD of 0.26, and MSC-422 has an OD of 0.26

and a FWHM of 26 nm. At 40 min, MSC-399 disappears completely, and MSC-422 reaches its maximum strength with an OD of 0.38 and a FWHM of 22 nm. MSC-422 evolved in Figure 1d has a smaller OD of 0.33 and a larger FWHM of 37 nm. From 40 to 60 min, little change is observed. Pathway 2 is followed.

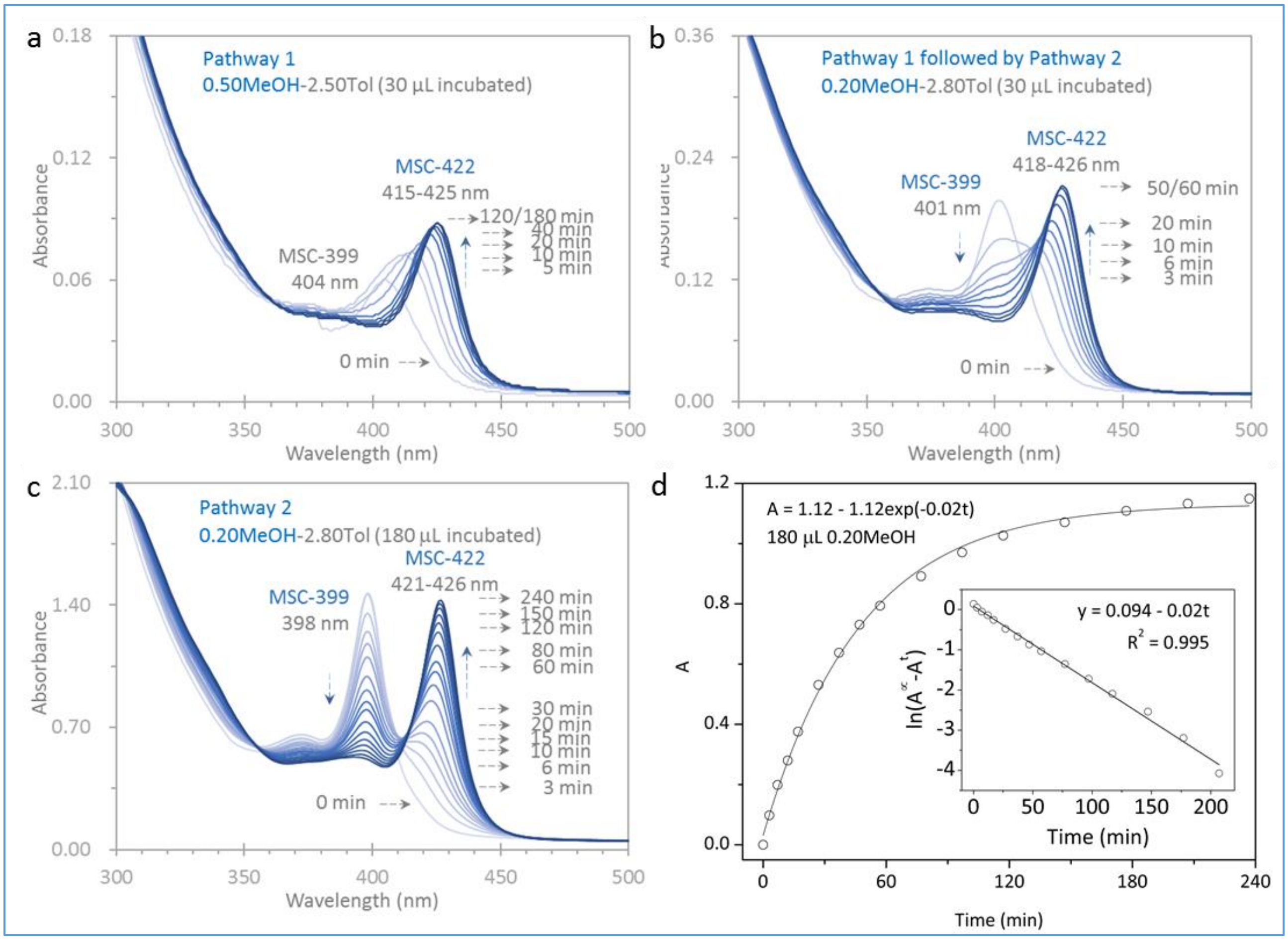

Figure S6-1. Isomerization in three MeOH-containing dispersions. The MSC-399 sample (30 μL (a and b), 180 μL (c)) is dispersed in the mixture (3.00 mL) of Tol and MeOH (0.50 mL (a), 0.20 mL (b and c)). MeOH has M_w = 32, $K_a = 10^{-15.5}$. Part d is the kinetic study of the evolution of MSC-422 in the reaction of Dispersion c. From Dispersions a to c, the ratio of the MSC-399 amount to the MeOH amount increases, and the isomerization proceeds via Pathway 1 (a) to Pathway 2 (c).

For Dispersion a, nine spectra are collected with the interval of 5 min from 0 to 10 min, 20 min from 20 to 80 min, at 120, and at 180 min. At 0 min, a peak is at 404 nm with an OD of 0.06 and a FWHM of 32 nm. At 5 min, this peak redshift to 411 nm, with an OD of 0.07 and FWHM of 27 nm. At 120 min, the peak arrives at 425 nm, with an OD of 0.08 and a FWHM of 20 nm. Then, the peak change little until 180 min. Pathway 1 is followed.

For Dispersion b, ten spectra are collected at 0, 3, 6, and 10 min, with an interval of 5 min from 10 to 20 min, and 10 min from 30 to 60 min. At 0 min, a peak is at 401 nm with an OD of 0.19 and a FWHM of 26 nm. At 3 min, this peak redshifts to 403 nm with an OD of 0.15, and a bump on the red side. At 6 min, the strength of MSC-399 decreases to 0.12, while the bump evolves into a peak at 416 nm with an OD of 0.14, indicating the evolution of MSC-422. Afterwards, the strength of MSC-399 keeps decreasing, and MSC-422 keeps increasing and red shifting. At 50 min, MSC-399 disappears completely, and MSC-422 has an OD of 0.20 and a FWHM of 19 nm. From 50 to 60 min, little change is observed. The isomerization proceeds via Pathways 1 and then Pathway 2.

For Dispersion c with the smallest amount of MeOH and largest amount of MSC-399, 17 spectra are collected up at 0, 3, 6, and 10 min, with an interval of 5 min from 10 to 20 min, 10 min from 20 to 60 min, 20 min from 60 to 120 min, and 30 from 120 to 240 min. At 0 min, MSC-399 peaks at 398 nm with an OD of 1.40 and a FWHM of 16 nm. At 3 min, MSC-399 changes little in strength (OD = 1.39), and a red-side bump develops. At 6 min, the OD of MSC-399 decreases 8% (OD = 1.28), while the bump develops into a peak at ~420 nm. At 10 min, MSC-399 has an OD of 1.18 and MSC-422 increases in strength peaking at 420 nm and has an OD of 0.53. At 240 min, MSC-399 disappears and MSC-422 peaks at 426 nm with an OD of 1.35 and a FWHM of 17 nm. The isomerization from MSC-399 to MSC-422 proceeds via Pathway 2 mainly with a distinctive isosbestic point at 412 nm.

Part d shows that the evolution of MSC-422 in Dispersion c follows first-order reaction kinetic behavior with a rate constant of 0.02 min^{-1}, and the R^2 value of 0.995. The net absorbance at 426 nm (denoted as A, open circles) is obtained by subtracting the absorbance at 3 min. Pathway 1 contributes little.

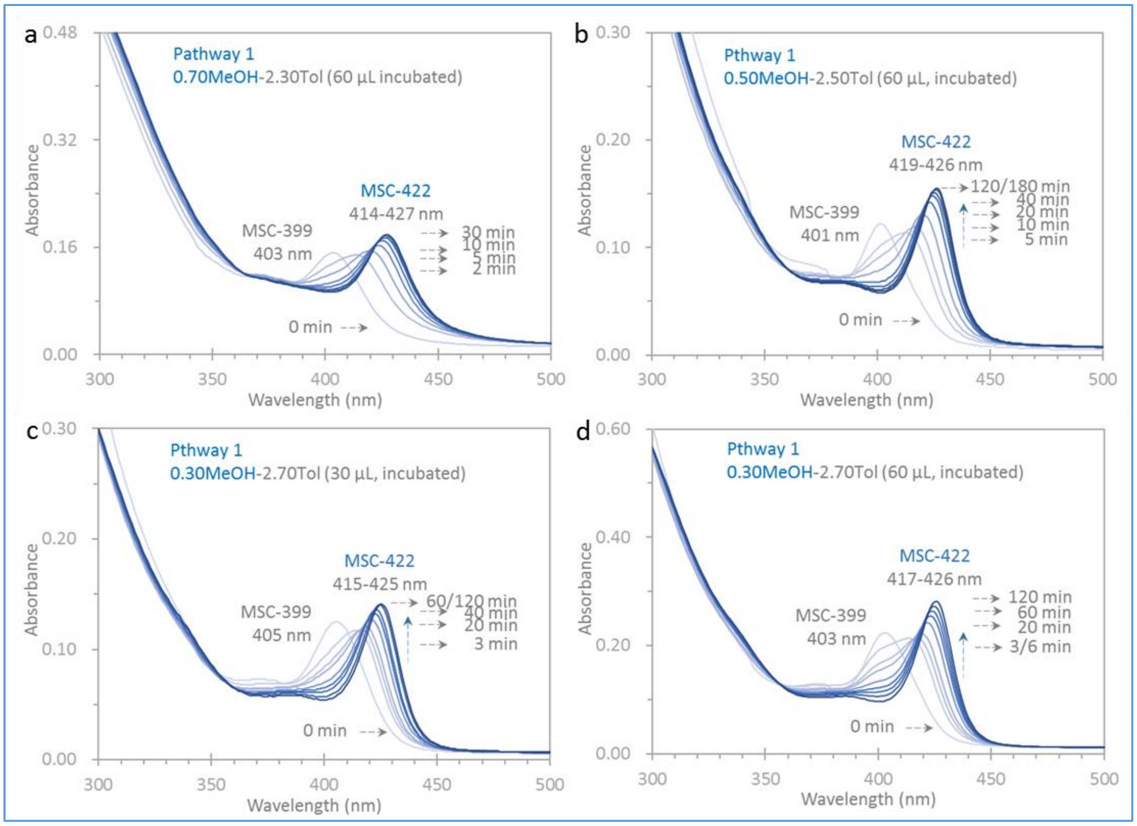

Figure S6-2. Pathway 1 in the four MeOH-containing dispersions. The spectra are collected from four dispersions with the MSC-399 sample (60 µL (a, b, and d) and 30 µL (c)) in the mixture of Tol and MeOH (0.70 mL (a), 0.50 mL (b), and 0.30 mL (c and d)). At 0 min, the peak is at 403 nm (a and d), 401 nm (b), and 405 nm (c), with a broad FWHM, indicating that the isomerization follows Pathway 1.

For Dispersion a, eight spectra are collected at 0, 2, and 5 min, with an interval of 5 min from 10 to 30 min.

For Dispersion b, nine spectra are collected with an interval of 5 min from 0 to 10 min, 20 min from 20 to 80 min, and at 120 and 180 min.

For Dispersions c and d, both nine spectra are collected at 0, 3, 6, and 10 min, with an interval of 10 min from 20 to 40 min, and at 60 and 120 min.

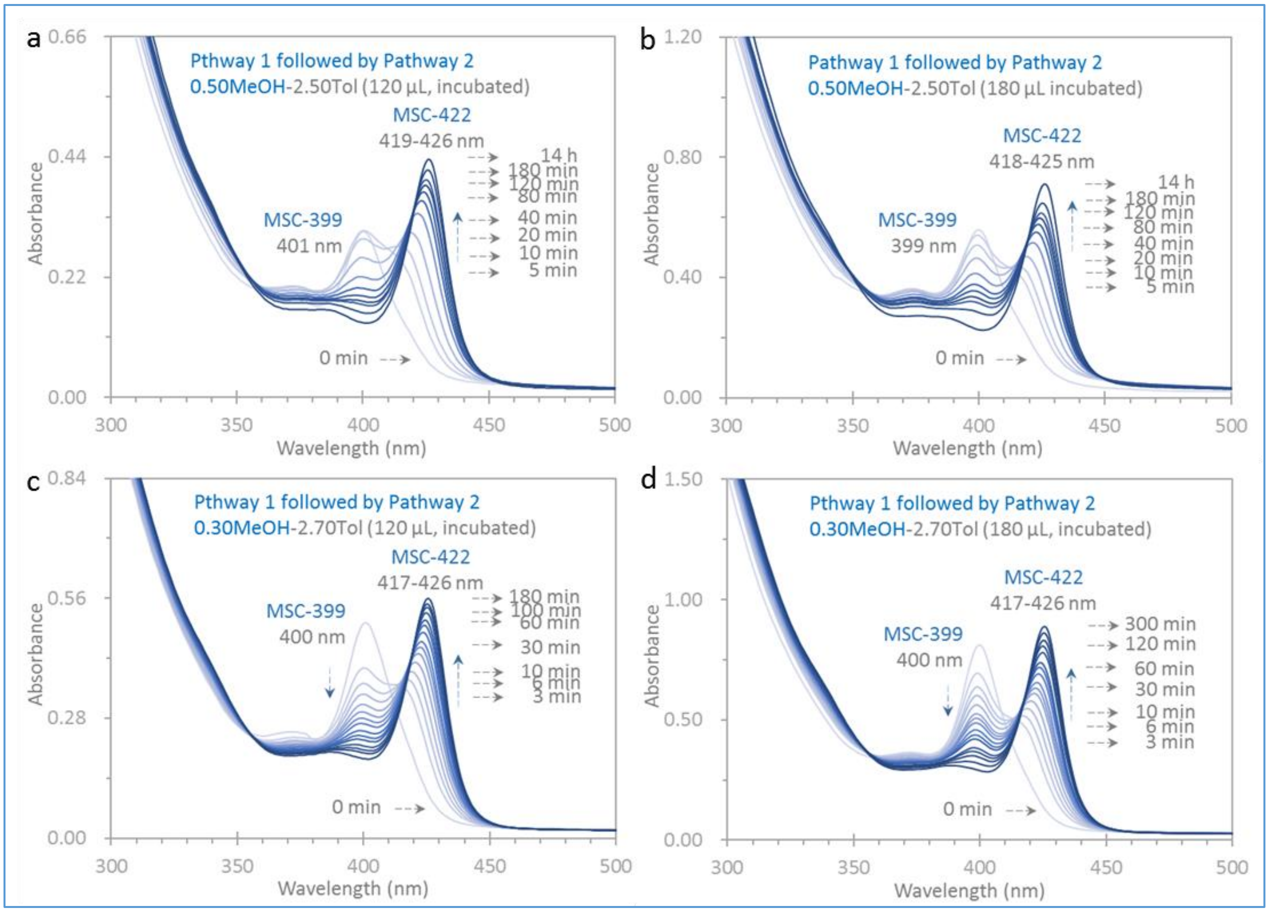

Figure S6-3. Pathway 2 after Pathway 1 in four MeOH-containing dispersions. The MSC-399 sample (120 µL (a and c) and 180 µL (b and d)) is dispersed in 2.50Tol-0.50MeOH (top panel, a and b) and 2.70Tol-0.30MeOH mixtures (bottom panel, c and d). Pathway 1 occur first, followed by Pathway 2. The relatively MeOH amount decreases from a to b, and from c to d, as well from a to c, and from b to d. The less the MeOH amount, the faster Pathway 2 is followed.

For Dispersions a and b, both 11 spectra are collected with an interval of 5 min from 0 to 10 min, 20 min from 20 to 120 min, and at 180 min and 14 h.

For Dispersions c and d, 15 and 16 spectra are collected respectively, at 0, 3, 6, and 10 min, with an interval of 5 min from 15 to 30 min, 10 min from 40 to 60 min, 20 min from 80 to 120 min, and at 180 min and at 300 min (for Dispersion d).

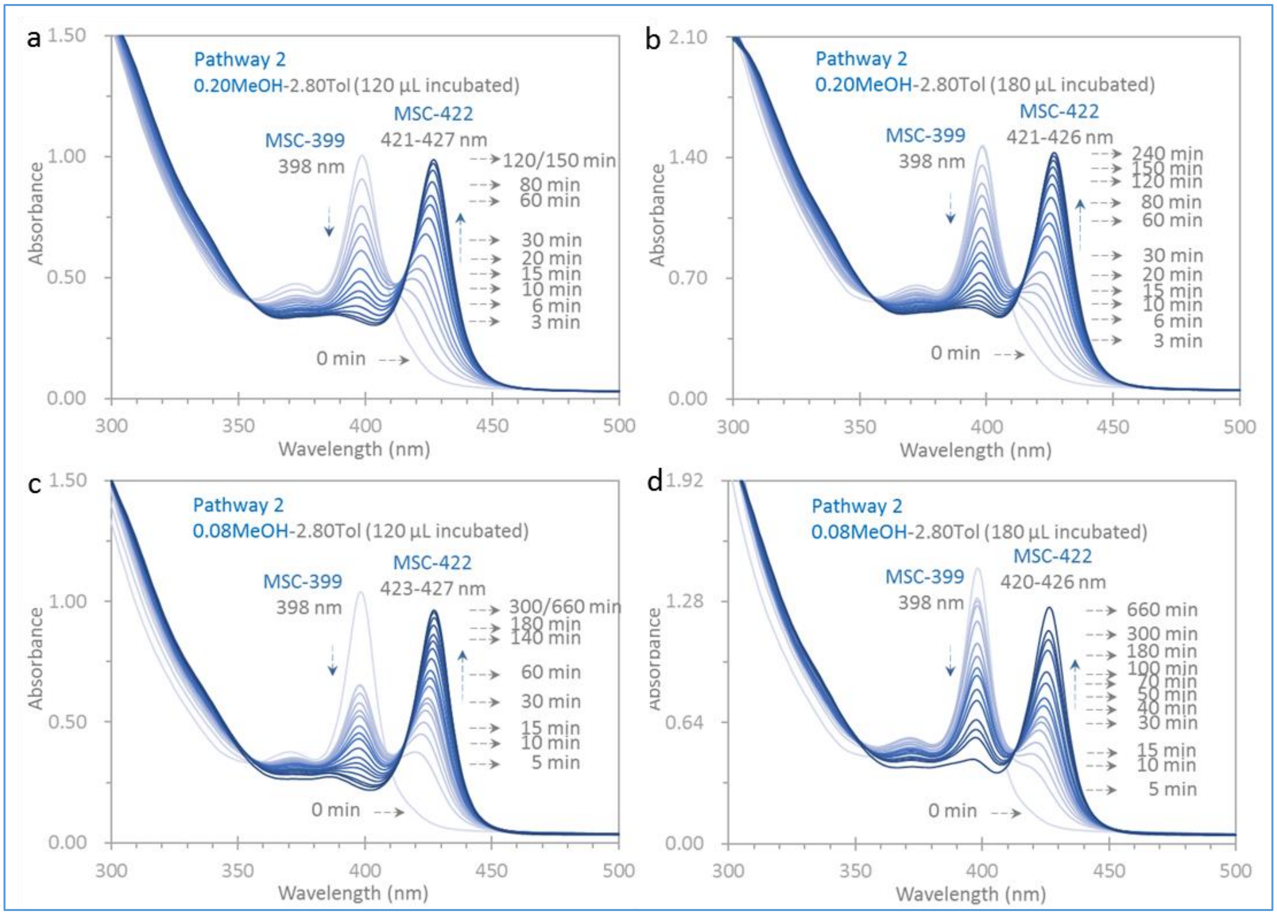

Figure S6-4. Pathway 2 mainly in four MeOH-containing dispersions. The MSC-399 sample (120 μL (a and c) and 180 μL (b and d)) is dispersed in 2.80Tol-0.20MeOH (top panel, a and b) and 2.92Tol-0.08MeOH mixtures (bottom panel, c and d). Pathway 2 is followed mainly with a distinctive isosbestic point. The relatively MeOH amount decreases from a to b, and from c to d, as well from a to c, and from b to d. The less the MeOH amount, the faster Pathway 2 is followed.

For Dispersion a, 14 spectra are collected at 0, 3, 6, and 10 min, with an interval of 5 min from 10 to 20 min, 10 min from 30 to 60 min, 20 min from 80 to 120 min, and at 150 min,

For Dispersion b, 17 spectra are collected at 0, 3, 6, and 10 min, with an interval of 5 min from 10 to 20 min, 10 min from 30 to 60 min, 20 min from 80 to 120 min, 30 min from 150 to 240 min.

For Dispersion c, 18 spectra are collected with an interval of 5 min from 0 to 30 min, and 10 min from 40 to 60 min, 20 min from 80 to 140 min, 60 min from 180 to 300 min, and at 660 min.

For Dispersion d, 15 spectra are collected with an interval of 5 min from 0 to 30 min, 10 min from 40 to 50 min, at 70 and 100 min, 60 min from 180 to 300 min, and at 660 min.

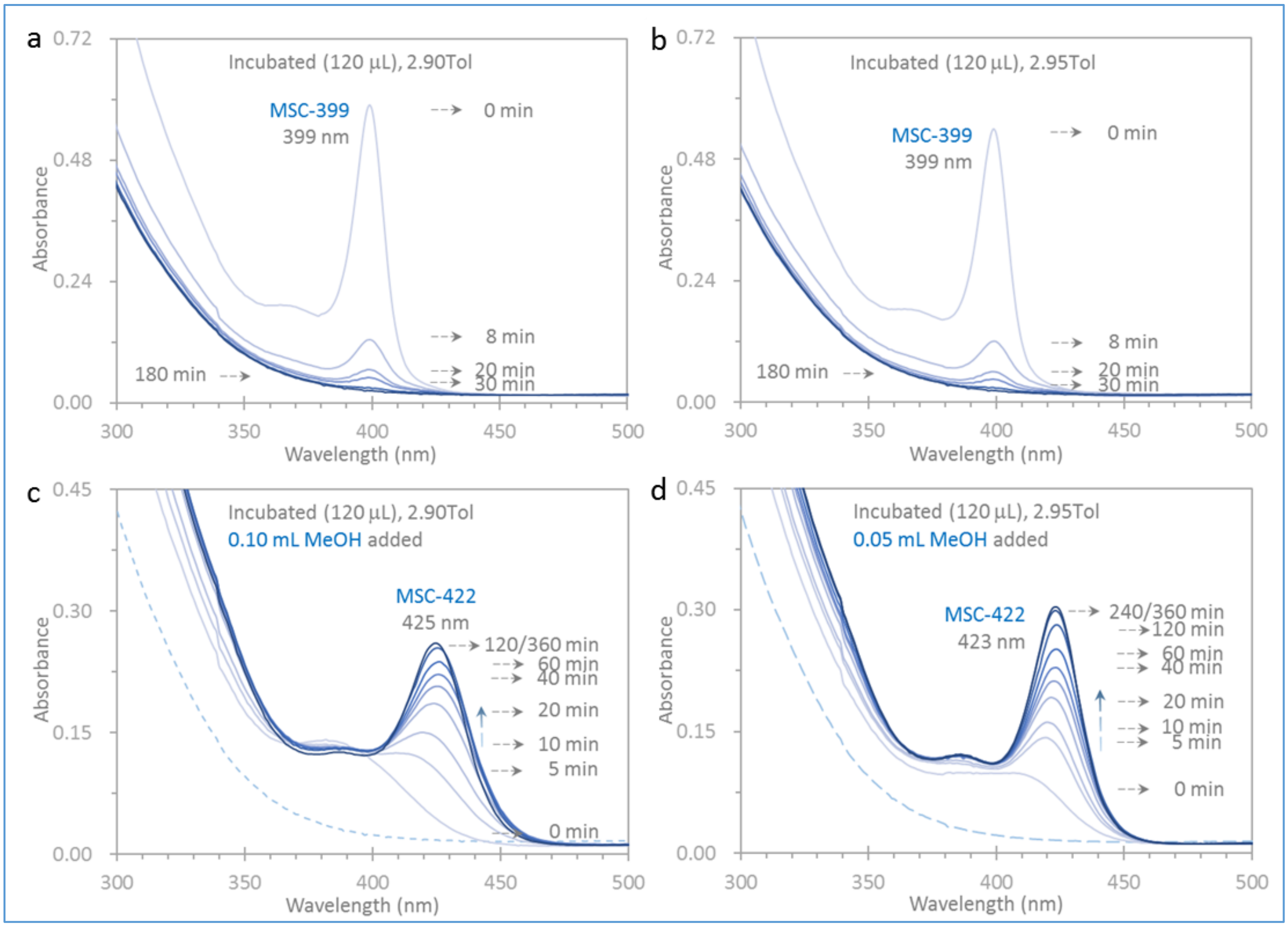

Figure S6-5. MeOH-induced CdTeSe MSC-422 in two Tol dispersions. Two dispersions are made with the MSC-399 sample dispersed in 2.90 (a) and 2.95 (b) mL of Tol. The spectra are collected at 0, 8, 20, 30, 60, and 180 min. At 0 min, in Dispersions a and b, MSC-399 peaking at 399 nm with OD of 0.57 (a) and 0.52 (b), and with a FWHM of 14 nm (a and b). MSC-399 disappears completely at 60 min. After the collection at the 180 min point, 0.10 (c) and 0.05 (d) mL of MeOH is added, respectively. The resulting dispersions are collected with 0, 5, 10, 20, 30, 40, 60, 120, 240 (for Dispersion d), and at 360 min.

In Dispersion c, a bump evolves at 5 min and becomes a peak at 10 min with an OD of 0.13 and a FWHM of 36 nm. MSC-422 reaches its maximum strength at 120 min with an OD of 0.23 and a FWHM of 30 nm; from 120 to 360 min, little change is observed.

In Dispersion d, MSC-422 evolves at 5 min, peaking at 420 nm with an OD of 0.13 and a FWHM of 29 nm. At 240 min, MSC-422 reaches its maximum with an OD of 0.29 and a FWHM of 23 nm.

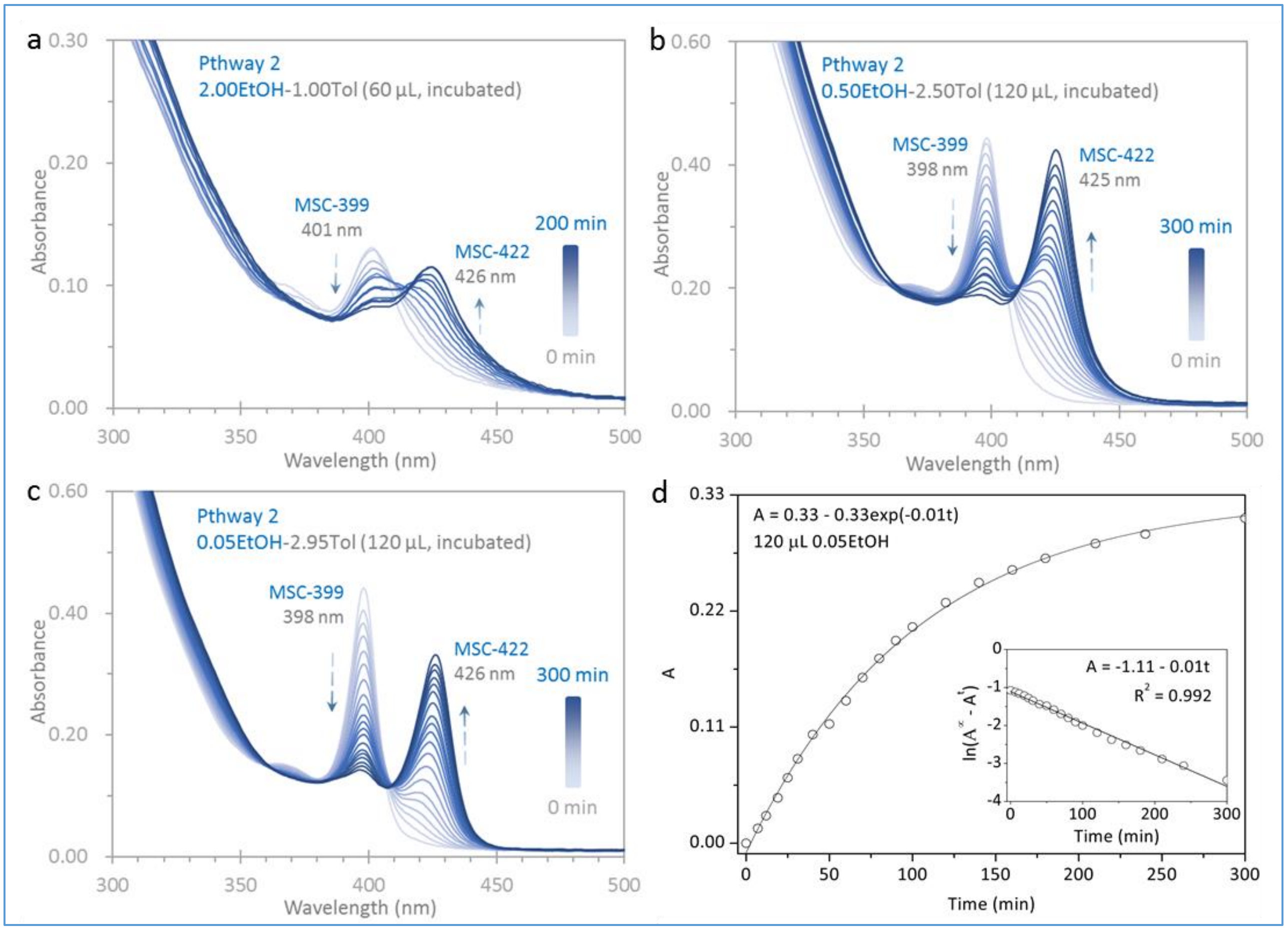

Figure S7-1. Three EtOH-containing dispersions. The three dispersions are made from the MSC-399 sample (60 µL (a), 120 µL (b and c)) in the mixture (3.00 mL) of Tol and EtOH (2.00 mL (a), 0.50 mL (b), and 0.05 mL (c)). EtOH has $M_w = 46$, $K_a = 10^{-16.0}$. In Dispersion a, Pathway 1 is followed first. In Dispersions b and c, Pathway 2 is followed mainly with a distinctive isosbestic point at ~410 nm. Part d shows that the evolution of MSC-422 in the reaction of Dispersion c follows the behavior of first-order reaction kinetics with a rate constant of 0.01 min^{-1}.

For Dispersion a, 12 spectra are collected at 0, 2, and 5 min, with an interval of 10 min from 10 to 30 min, 20 min from 50 to 70 min, and 30 min from 100 to 160 min, and at 200 min.

For Dispersion b, 21 spectra are collected with an interval of 6 min from 0 to 18 min, at 25 min, 10 min from 30 to 120 min, 20 min from 140 to 160 min, 30 min from 180 to 240 min, and at 300 min.

For Dispersion c, 20 spectra are collected at 0, 7, 12, 19, and 25 min, with an interval of 10 min from 30 to 100 min, and 20 min from 120 to 180 min, 30 min from 210 to 240 min, and at 300 min.

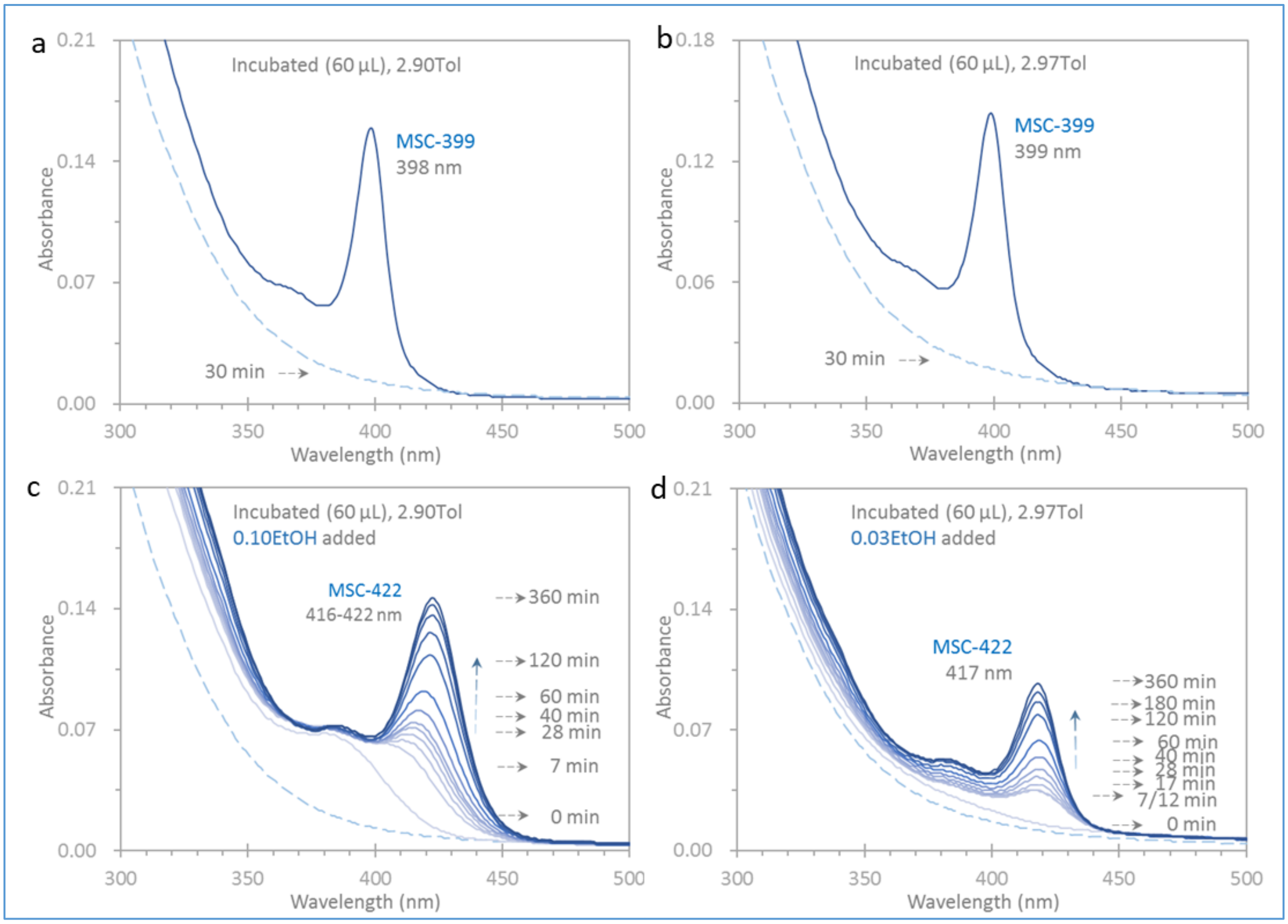

Figure S7-2. EtOH-induced CdTeSe MSC-422 in two Tol dispersions. The MSC-399 sample is dispersed in 2.90 (a) and 2.95 (b) mL Tol, and MSC-399 at 0 min has an OD of 0.16 (a) and 0.14 (b), and a FWHM of 15 nm (a and b). MSC-399 disappears completely at 30 min. Afterwards, 0.10 (c) and 0.05 (d) mL EtOH is added, respectively. 11 spectra are collected at 0, 7, 12, 17, 28, 40, 60, 120, 180, 240, and 360 min.

For Dispersion c, at 12 min, a peak has an OD of 0.06 and a FWHM of 30 nm. At 360 min, this peak redshifts to 422 nm with an OD of 0.14 and a FWHM of 25 nm.

For Dispersion d, MSC-422 evolves at 7 min, and keeps increasing. At 360 min MSC-422 has an OD of 0.09 and a FWHM of 18 nm.

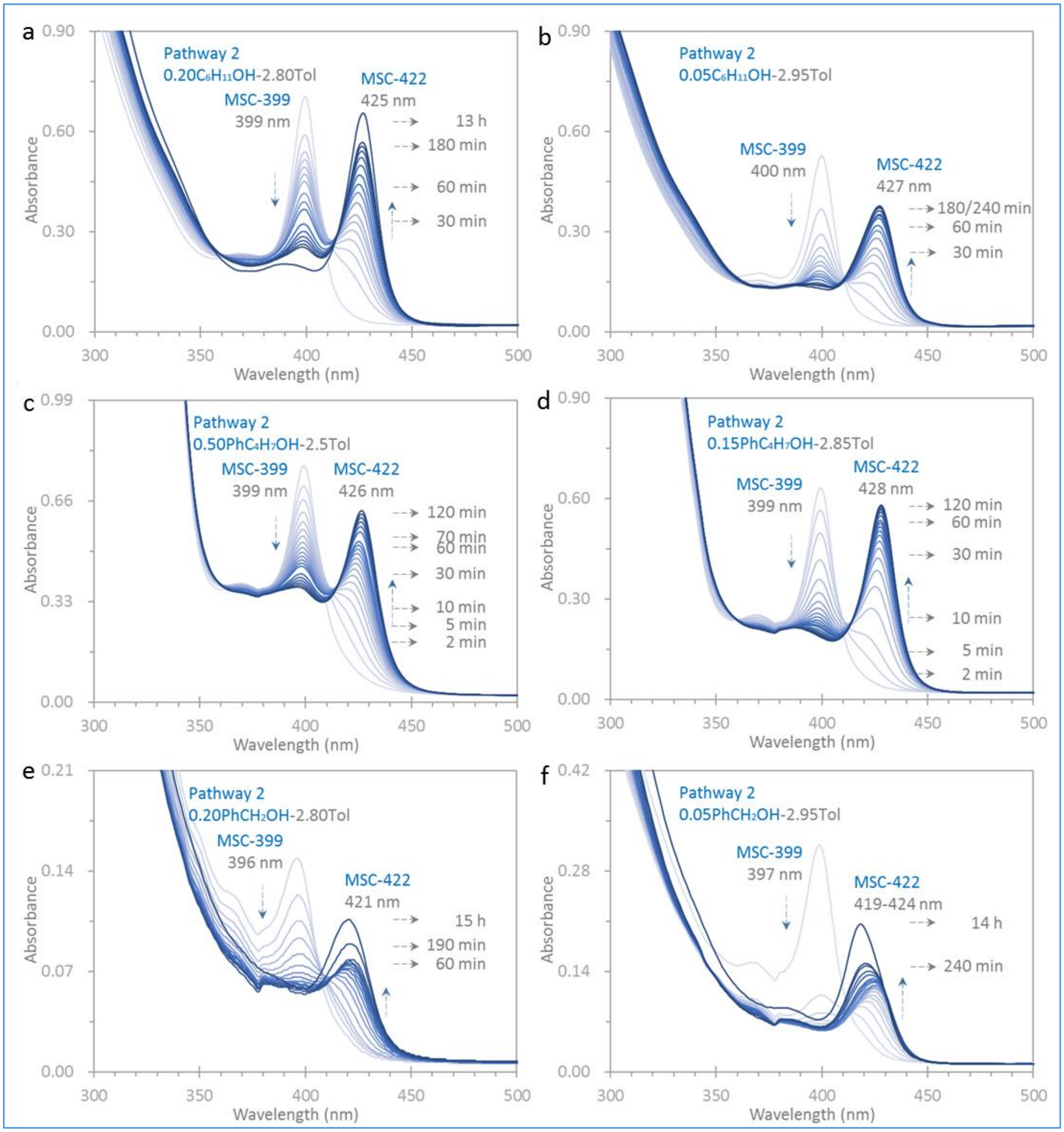

Figure S8-1. Optical absorption spectroscopy study of the isomerization from MSC-399 to MSC-422 in six alcohol-containing dispersions. Three alcohols with different steric hindrance and acidity, are cyclohexanol ($C_6H_{11}OH$, M_w = 100, K_a = $10^{-16.0}$) (a and b), 1,2,3,4–tetrahydro-1-naphthol (PhC_4H_7OH, M_w = 148, K_a = $10^{-14.3}$) (c and d), and phenylmethanol ($PhCH_2OH$, M_w = 94, K_a = $10^{-14.4}$) (e and f). The amount of $C_6H_{11}OH$ and $PhCH_2OH$ used is 0.20 (a and e) and 0.05 (b

and f) mL, and that of PhC_4H_7OH is 0.50 (c) and 0.15 (d) mL. The isomerization pathway is regulated by mainly by the acidity of the incoming ligand, as is also seen by Figure S8-2.

For Dispersions a and b, 17 spectra are collected with the interval of 5 min from 0 to 30 min, 10 min from 40 to 60 min, 20 min from 80 to 180 min, and at 240 min (for Dispersion b) and 13 h (for Dispersion a).

For Dispersions c and d, 20 spectra are collected at 0, 2, 5 min, and with the interval of 5 min from 10 to 60 min, and 10 min from 70 to 120 min.

For Dispersion e, 16 spectra are collected with the interval of 2 min from 0 to 6 min, 5 min from 10 to 50 min, at 60, 190 min, and at 15 h.

For Dispersion f, 18 spectra are collected with the interval of 2 min from 0 to 10 min, 5 min from 15 to 30 min, 10 min from 40 to 60 min, at 90 min, 60 min from 120 to 240 min, and at 14 h.

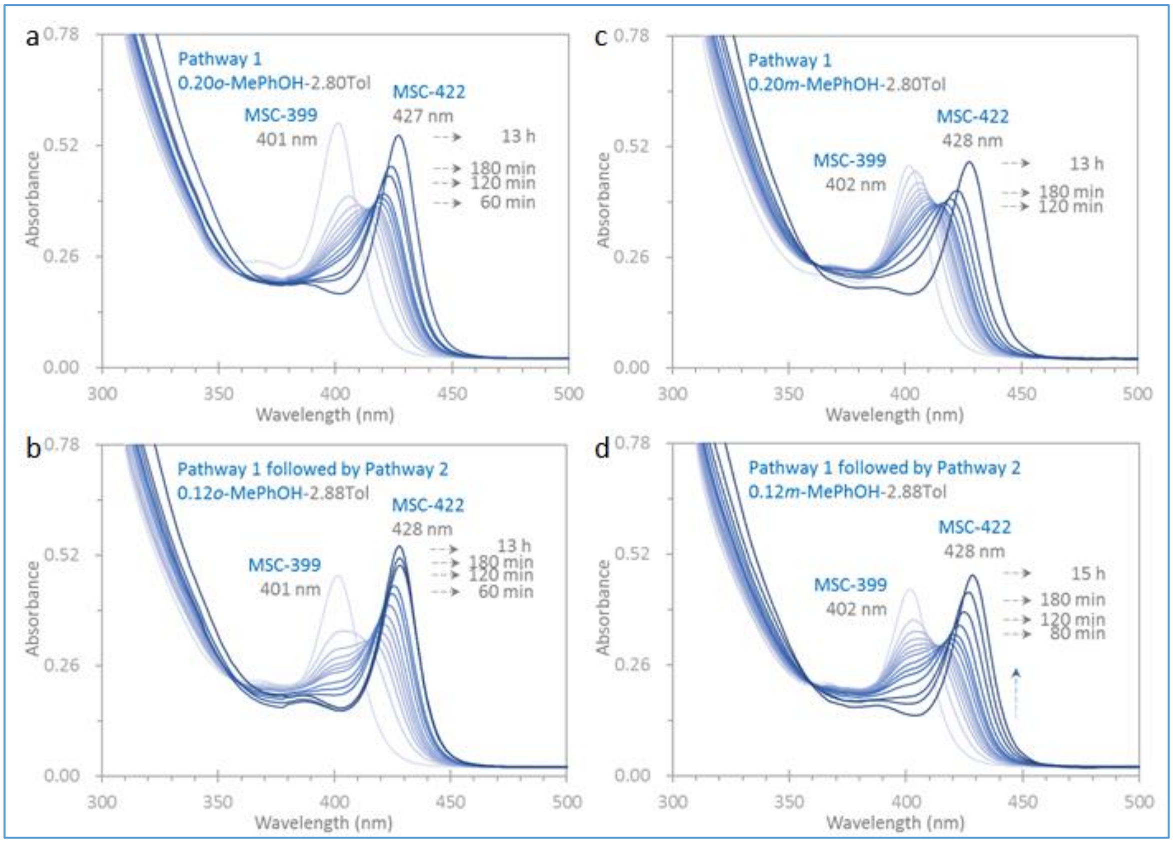

Figure S8-2. Four dispersions containing o/m-CH_3PhOH. o/m-CH_3PhOH is in the left/right panel (M_w = 108, K_a = $10^{-10.3}/10^{-10.1}$). The MSC-399 sample (120 μL) is dispersed in the mixture (3.00 mL) of Tol and o/m-CH_3PhOH (0.20 mL (top panel) and 0.12 mL (bottom panel)). The spectra are collected in situ with the interval of 5 min from 0 to 30 min, 10 min from 30 to 60 min, at 80 min (c and d), 60 min from 120 to 180 min, and at 13 h (a, b and c) and 15 h (d). When the amount of o/m-CH_3PhOH is relatively large (top panel), Pathway 1 is followed more. The pathway is regulated mainly by ligand acidity, as is also seen by Figure S8-1.

For Dispersions a and b, 13 spectra are collected with an interval of 5 min from 0 to 30 min, 10 min from 40 to 60 min, 60 min from 120 to 180 min, and at 13 h.

For Dispersions c and d, 14 spectra are collected with an interval of 5 min from 0 to 30 min, 10 min from 40 to 60 min, at 80 min, 60 min from 120 to 180 min, and at 13 h (c) and 15 h (d).

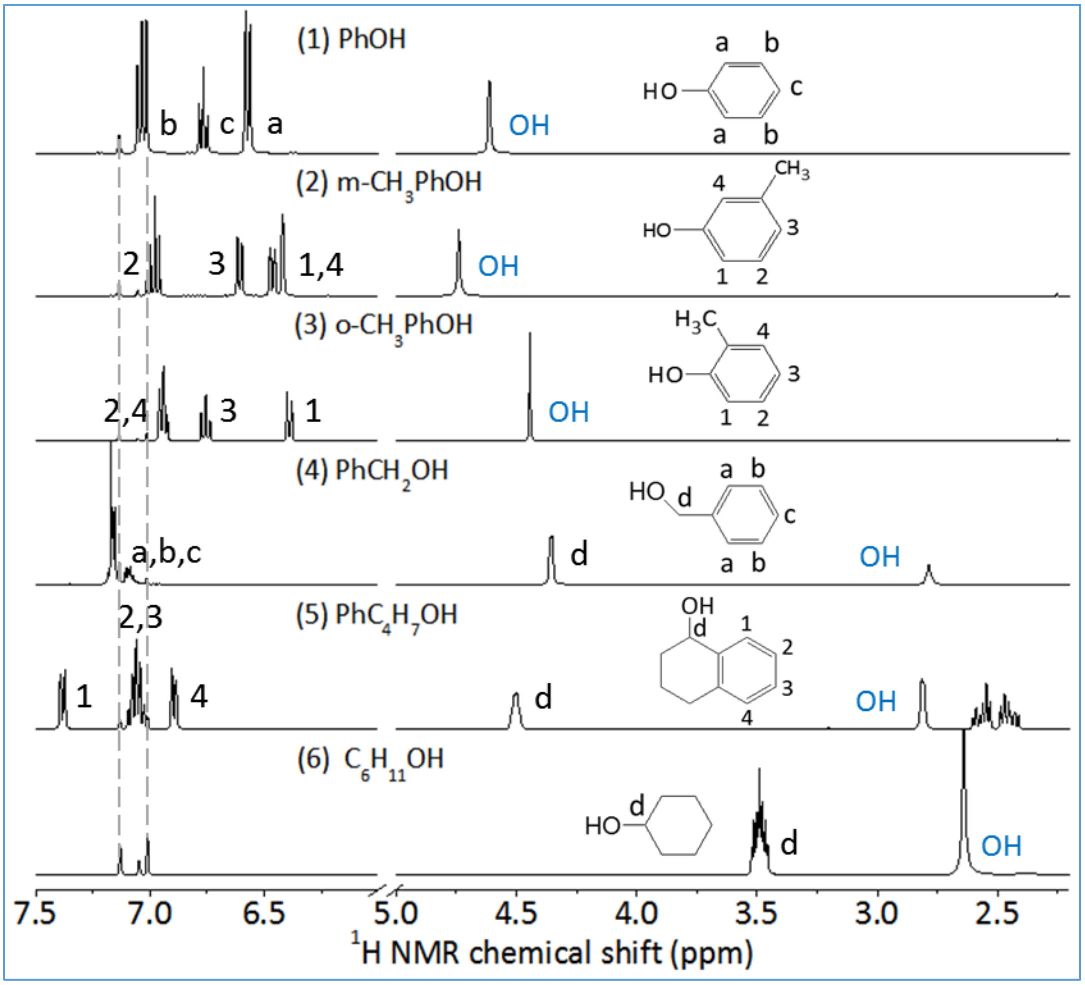

Figure S8-3. ^{1}H NMR of six alcohols in Tol-d_8 at room temperature. The six mixtures are prepared by mixing the alcohol and Tol-d_8 (560 μL), which are PhOH (20 μL, Trace 1, $K_a = 10^{-10.0}$), m-CH_3PhOH (32 μL, Trace 2, $K_a = 10^{-10.1}$), o-CH_3PhOH (40 μL, Trace 3, $K_a = 10^{-10.3}$), $PhCH_2OH$ (40 μL, Trace 4, $K_a = 10^{-14.4}$), PhC_4H_7OH (52 μL, Trace 5, $K_a = 10^{-14.3}$), and $C_6H_{11}OH$ (40 μL, Trace 6, $K_a = 10^{-16.0}$). The resulting solutions have similar molar concentrations. For Traces 1 and 6, the signals of −OH group are respectively located at 4.61, 4.74, 4.44, 2.79, 2.82, and 2.64 ppm. The acidity of the first three compounds seems to be larger than that of the last three compounds. The last three compounds are dealt with in Figure S8-1, while the second and third compound in Figure S8-2.

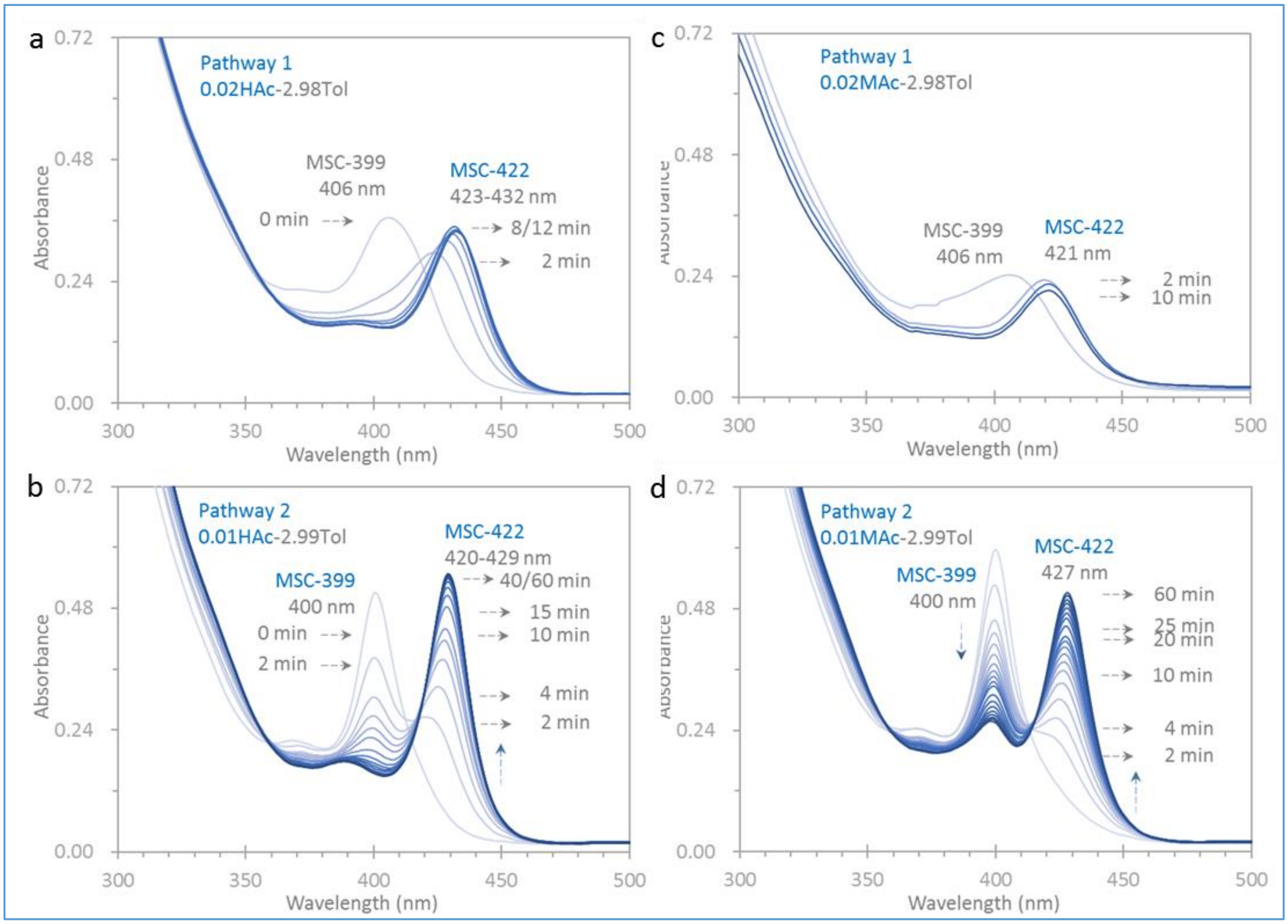

Figure S9. Carboxylic acid-containing dispersions. The acid is acetic acid (HAc, M_w = 60, $K_a = 10^{-4.8}$, left panel) and formic acid (MAc, M_w = 46, $K_a = 10^{-3.7}$, right panel). The four dispersions are prepared with the MSC-399 sample (120 μL) in the mixture (3.00 mL) of Tol and acid (0.02 mL (top panel) or 0.01 mL (bottom panel). When the acid amount is relatively large, Pathway 1 is followed. When the acid amount is relatively small, Pathway 2 is followed. Pathway 1 is faster in Dispersion b than Dispersion a. Pathway 2 is faster in Dispersion c than in Dispersion d. The incoming ligand with relatively large acidity prefers Pathway 1.

For Dispersion a, 7 spectra are collected with an interval of 2 min from 0 to 12 min.

For Dispersion b, 16 spectra are collected with an interval of 2 min from 0 to 10 min, and 5 min from 10 to 60 min.

For Dispersion c, 4 spectra are collected at 0, 2, 6, and 10 min.

For Dispersion d, 19 spectra are collected with an interval of 2 min from 0 to 20 min, and 5 min from 20 to 60 min.

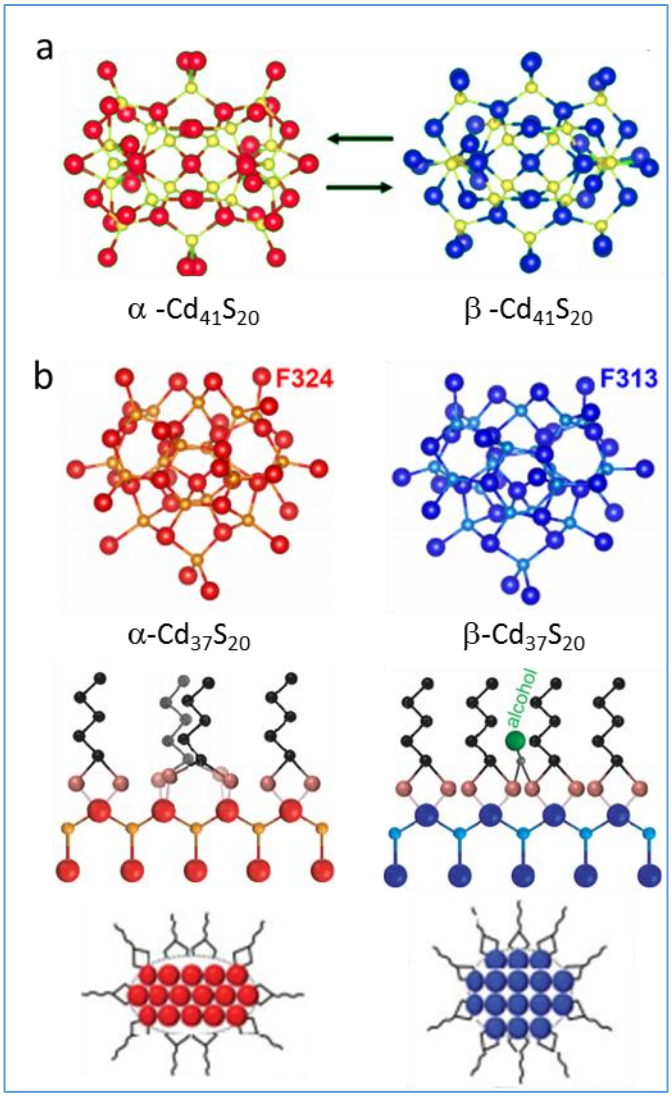

Figure S10-1. Examples of ongoing effects for the composition study of MSCs. A composition of $Cd_{41}S_{20}$ (a)[21] is announced this year. For CdS MSCs, CdS MSC-324 was reported in 1984,[22] and each cluster was suggested to have 17 to 32 Cd atoms in 2018.[23] A composition of $Cd_{37}S_{20}$ was claimed for both CdS MSC-313 and CdS MSC-324 in 2019 (b),[10] while $Cd_{37}S_{18}$ and $Cd_{37}S_{32}$ in 2022.[24,25] In a side note, the thermally-induced and/or alcohol-triggered isomerization between CdS MSC-311 and MSC-322 was documented.[10,5] For the MeOH-induced MSC-322 to MSC-311 isomerization, MeOH (green) was suggested to interact with the oleate ligand without ligand exchange.[10] For CdSe MSC-415 (with optical absorption peaking in the range of 403 to 420 nm), a composition of $Cd_{34}Se_{16}$ was reported in 1998,[26] $Cd_{33}Se_{33}$/$Cd_{34}Se_{34}$ with a core-caged structure in 2004,[27] $Cd_{84}Se_{56}$ in 2014,[28] ellipsoidal $Cd_{34}Se_{34}$ in 2018,[29] $Cd_{84}Se_{84}$ in 2021,[30] and $Cd_{32}Se_{16}$ in

2022.[25] The research on MSCs is in an early stage, searching for the composition of the MSC.

(5) Zhang, B.; Zhu, T.; Ou, M.; Rowell, N.; Fan, H.; Han, J.; Tan, L.; Dove, M. T.; Ren, Y.; Zuo, X.; Han, S.; Zeng, J.; Yu, K. Thermally-induced reversible structural isomerization in colloidal semiconductor CdS magic-size clusters. *Nat. Commun*. **2018**, *9*, 2499.

(10) Williamson, C. B.; Nevers, D. R.; Nelson, A.; Hadar, I.; Banin, U.; Hanrath, T.; Robinson, R. D. Chemically reversible isomerization of inorganic clusters. *Science* **2019**, *363*, 731−735.

(21) Shim, D.; Lee, J.; Kang, J. Multiscale isomerization of magic-sized inorganic clusters chemically driven by atomic-bond exchanges. *Chem. Mater.* **2022**, 34, 9527–9535.

(22) Fojtik, A.; Weller, H.; Koch, U.; Henglein, A. Photo-physics of extremely small CdS particles: Q-state CdS and magic agglomeration numbers. *Ber. Bunsenges. Phys. Chem.* **1984**, *88*, 969−977.

(23) Nevers, D. R.; Williamson, C. B.; Savitzky, B. H.; Hadar, I.; anin, U.; Kourkoutis, L. F.; Hanrath, T.; Robinson, R. D. Mesophase formation stabilizes high-purity magic-sized clusters. *J. Am. Chem. Soc.* **2018**, *140*, 3652−3662.

(24) Han, H.; Kallakuri, S.; Yao, Y.; Williamson, C. B.; Nevers, D. R.; Savitzky, B. H.; Skye, R. S.; Xu, M.; Voznyy, O.; Dshemuchadse, J.; Kourkoutis, L. F.; Weinstein, S. J.; Hanrath, T.; Robinson, R. D. Multiscale hierarchical structures from a nanocluster mesophase. *Nat. Mater*. **2022**, *21*, 518−525.

(25) He, L.; Luan, C.; Liu, S.; Chen, M.; Rowell N.; Wang; Z.; Li, Y.; Zhang, C.; Lu, J.; Zhang, M.; Liang, B.; Yu, k.; Transformations of magic-size clusters via precursor compound cation exchange at room temperature. *J. Am. Chem. Soc*. **2022**, *144*, 19060−19069.

(26) Ptatschek, V.; Schmidt, T.; Lerch, M.; Miiller, G.; Spanhel, L.; Emmerling, A.; Fncke, J.; Foitzik, A. H.; Langer, E. Quantized aggregation phenomena in II−VI-semiconductor colloids. *Ber. Bunsenges. Phys. Chem*. **1998**, *102*, 85−95.

(27) Kasuya, A.; Sivamohan, R.; Barnakov, Y. A.; Dmitruk, I. M.; Nirasawa, T.; Romanyuk, V. R.; Kumar, V.; Mamykin, S. V.; Tohji, K.; Jeyadevan, B.; Shinoda, K.; Kudo, T.; Terasaki, O.; Liu, Z.; Belosludov, R. V.; Sundararajan, V.; Kawazoe, Y. Ultra-stable nanoparticles of CdSe revealed from mass spectrometry. *Nat. Mater*. **2004**, *3*, 99−102.

(28) Beecher, A. N.; Yang, X.; Palmer, J. H.; LaGrassa, A. L.; Juhas, P.; Billinge, S. J.; Owen, J. S. Atomic structures and gram scale synthesis of three tetrahedral quantum dots. *J. Am. Chem. Soc.* **2014**, *136*, 10645−10653.

(29) Hsieh, T.; Yang, T.; Hsieh, C.; Huang, S.; Yeh, Y.; Chen, C.; Li, E.; Liu, Y. Unraveling the structure of magic-size $(CdSe)_{13}$ cluster pairs. *Chem. Mater.* **2018**, *30*, 5468−5477.

(30) Mule, A. S.; Mazzotti, S.; Rossinelli, A. A.; Aellen, M.; Prins, P. T.; Bok, J. C. V. D.; Solari, S. F.; Glauser, Y. M.; Kumar, P. V.; Riedinger, A.; Norris, D. J. Unraveling the growth mechanism of magic-sized semiconductor nanocrystals. *J. Am. Chem. Soc.* **2021**, *143*, 2037−2048.

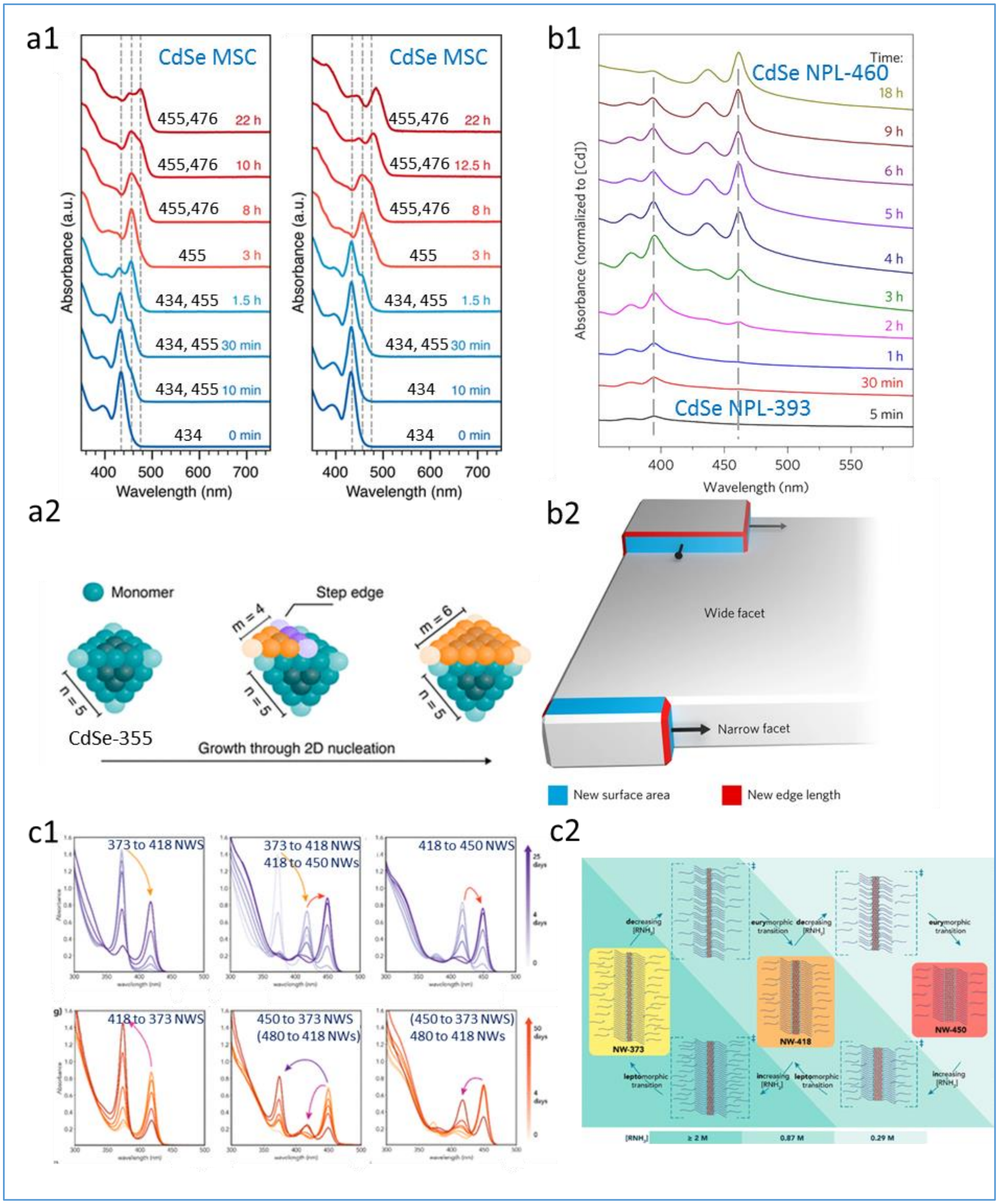

Figure S10-2. Examples of temporal evolution of optical absorption properties reported and corresponding transformation patterns suggested. Thanks to the optical absorption property, the pathway study for the formation and transformation has started (although the composition and structure is not clear yet (Figure S10-1)). The continuous redshift observed in MSCs suggests when a change directly occurs on the mother nano-species, a continuous rather than

step-wise shift is necessarily seen. When a change exhibits a step-wise shift, it shall not occur directly on the mother nano-species. The mother nano-species include 0D MSCs, 1D nanowires, 2D NPLs, and 0D quantum dots (QDs). Our results challenge the common hypothesis which claims that upon monomer addition, CdSe 0D clusters enlarge or 2D nanoplatelets (NPLs) become thicker, in which cases a step-wise redshift is observed; the intermediate does not have a measurable optical absorption.[30,31] But it is difficult to understand WHY the intermediate is transparent and not measurable.

(a1 and a2) For the redshift monitored from two reactions at 180 °C, the addition of one CdSe monolayer is suggested for the transformation of the CdSe MSCs; the addition is suggested to be on one of the four identical facets of a CdSe tetrahedra cluster. MSC-355, -380, 408, -434, -455, and -476 respectively have 5 to 10 monolayer.[30] The redshift seems to be step-wise. It is not easy to distinguish between the continuous and step-wise shift according to spectra that are offset (Figure S10-4).

(b1 and b2) For the step-wise shift monitored during a high temperature reaction at 200 °C, the transformation of the CdSe nanoplatelets (NPLs) from NPL-393 to NPL-460 is suggested, and is via the addition of one CdSe monolayer on the wide facet.[31] The NPL has a two-dimension (2D) morphology with 1D quantum confinement. NPL-393 has three layers of Cd and two layers of Se, while NPL-460 has four layers of Cd and three layers of Se. Different opinions have been expressed for the formation and transformation pathway of the NPLs.[32-42] A PC-enabled pathway has been argued (Figure S10-3).[36-38] At the same time, it has been demonstrated that the ready self-assembly of zero-dimension (0D) MSCs with three-dimension (3D) quantum confinement results in the formation of the 2D NPL, and they display similar static optical absorption with a sharp doublet.[36-42]

(c1 and c2) For the step-wise shift from 373 nm to 450 nm via 418 nm monitored during room temperature reactions, the transformation of 1D CdTe nanowires (NWs) with 2D quantum confinement from NW-373 to NW-450 via NW-418 is suggested with an increase of the diameter.[43] It is difficult to understand how these NWs "jump/transform" from one to the other without intermediates. A different explanation is that the nano-species are 0D MSCs and the transformation is PC-enabled.[9,16,17]

(9) He, L.; Luan, C.; Rowell, N.; Zhang, M.; Chen, X.; Yu, K. Transformations among colloidal semiconductor magic-size clusters. *Acc. Chem. Res.* **2021**, *54*, 776–786.

(16) Luan, C.; Tang, J.; Rowell, N.; Zhang, M.; Huang, W.; Fan, H.; Yu, K. Four types of CdTe magic-size clusters from one prenucleation stage sample at room temperature. *J. Phys. Chem. Lett*. **2019**, *10*, 4345–4353.

(17) Shen, Q.; Luan, C.; Rowell, N.; Zhang, M.; Wang, K.; Willis, M.; Chen, X.; Yu, K. Reversible transformations at room temperature among three types of CdTe magic-size clusters. *Inorg. Chem*. **2021**, *60*, 4243–4251.

(30) Mule, A. S.; Mazzotti, S.; Rossinelli, A. A.; Aellen, M.; Prins, P. T.; van der Bok, J. C.; Solari, S. F.; Glauser, Y. M.; Kumar, P. V.; Riedinger, A.; Norris, D. J. Unraveling the growth mechanism of magic-sized semiconductor nanocrystals. *J. Am. Chem. Soc*. **2021**, *143*, 2037–2048.

(31) Riedinger, A.; Ott, F. D.; Mule, A.; Mazzotti, S.; Knusel, P. N.; Kress, S. J. P.; Prins, F.; Erwin, S. C.; Norris, D. J. An intrinsic growth instability in isotropic materials leads to quasi-two-dimensional nanoplatelets. *Nat. Mater*. **2017**, *16*, 743–748.

(32) Ithurria, S.; Dubertret, B. Quasi 2D colloidal CdSe platelets with thicknesses controlled at the atomic level. *J. Am. Chem. Soc.* **2008**, *130*, 16504–16505.

(33) Ithurria, S.; Bousquet, G.; Dubertret, B. Continuous transition from 3D to 1D confinement observed during the formation of CdSe nanoplatelets. *J. Am. Chem. Soc*. **2011**, *133*, 3070–3077.

(34) Chen, Y.; Chen, D.; Li, Z.; Peng, X. Symmetry-breaking for formation of rectangular CdSe two-dimensional nanocrystals in zinc-blende structure. *J. Am. Chem. Soc.* **2017**, *139*, 10009–10019.

(35) Moghaddam, N.; Dabard, C.; Dufour, M.; Po, H.; Xu, X.; Pons, T.; Lhuillier, E.; Ithurria, S. Surface modification of CdE (E: S, Se, and Te) nanoplatelets to reach thicker nanoplatelets and homostructures with confinement-induced intraparticle type I energy level alignment. *J. Am. Chem. Soc*. **2021**, *143*, 1863–1872.

(36) Liu, Y.; Willis, M.; Rowell, N.; Luo, W.; Fan, H.; Han, S.; Yu, K. Effect of small molecule additives in the prenucleation stage of semiconductor CdSe quantum dots. *J. Phys. Chem. Lett*. **2018**, *9*, 6356−6363.

(37) Zhu, J.; Cao, Z.; Zhu, Y.; Rowell, N.; Li, Y.; Wang, S.; Zhang, C.; Jiang, G.; Zhang, M.; Zeng, J.; Yu, K. Transformation pathway from CdSe magic-size clusters with absorption doublets at 373/393 nm to clusters at 434/460 nm. *Angew. Chem. Int. Ed*. **2021**, *60*, 20358–20365.

(38) Cao, Z.; Zhu, J.; Peng, J.; Meng, N.; Bian, F.; Luan, C.; Zhang, M.; Li, Y.; Yu, K.; Zeng, J. Transformation pathway from CdSe nanoplatelets with absorption doublets at 373/393 nm to nanoplatelets at 434/460 nm. *J. Phys. Chem. Lett*. **2022**, *13*, 3983−3989.

(39) Ouyang, J.; Zaman, M. B.; Yan, F. J.; Johnston, D.; Li, G.; Wu, X.; Leek, D.; Ratcliffe, C. I.; Ripmeester, J. A.; Yu, K. Multiple families of magic-sized CdSe nanocrystals with strong bandgap photoluminescence via noninjection one-pot syntheses. *J. Phys. Chem. C* **2008**, *112*, 13805−13811.

(40) Yu, K.; Ouyang, J.; Zaman, M. B.; Johnston, D.; Yan, F. J.; Li, G.; Ratcliffe, C. I.; Leek, D. M.; Wu, X.; Stupak, J.; Jakubek, Z.; Whitfield, D. Single-sized CdSe nanocrystals with bandgap

photoemission via non-injection one-pot approach. *J. Phys. Chem. C* **2009**, *113*, 3390−3401.
(41) Liu, Y.; Zhang, B.; Fan, H. S.; Rowell, N.; Willis, M.; Zheng, X. T.; Che, R. C.; Han, S.; Yu, K. Colloidal CdSe 0-dimension nanocrystals and their self-assembled 2-dimension structures. *Chem. Mater*. **2018**, *30*, 1575−1584.
(42) Liu, Y.; Rowell, N.; Willis, M.; Zhang, M.; Wang, S.; Fan, H.; Huang, W.; Chen, X.; Yu, K. Photoluminescent colloidal nanohelices self-assembled from CdSe magic-size clusters via nanoplatelets. *J. Phys. Chem. Lett*. **2019**, *10*, 2794−2801.
(43) Busatto, S.; Spallacci, C.; Meeldijk, J. D.; Howes, S.; Donega, C. de M. Room-temperature interconversion between ultrathin CdTe magic-size nanowires induced by ligand shell dynamics. *J. Phys. Chem. C* **2022**, *126*, 15280–15297.

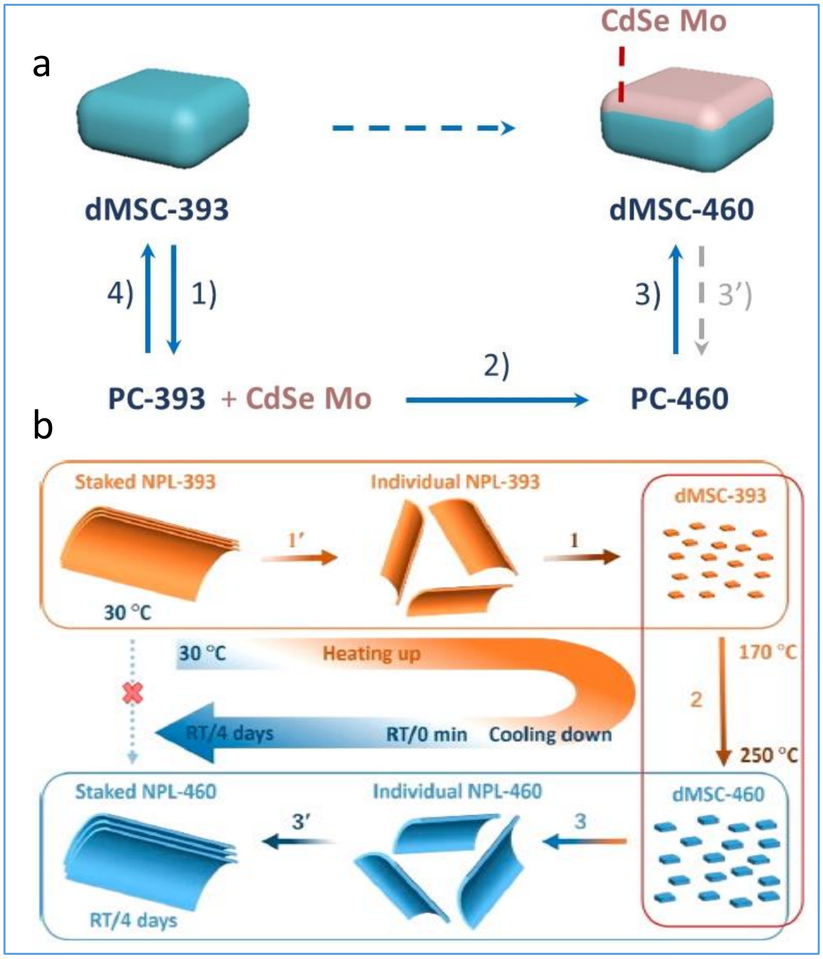

Figure S10-3. A PC-assisted formation and transformation argued.[36-38] (a) For the step-wise shift observed from 393 to 460 nm, the addition of CdSe monomers occurs on PC-393, and PC-460 is formed (Step 2).[37] (b) When temperature is increased from 30 to 170 °C, NPL-393 transforms to

dMSC-393. At 250 °C, dMSC-460 is seen (Step 2) due to the addition of CdSe monomers occurs on PC-393; when the temperature is lowed back to room temperature (RT), NPL-460 is observed from the self-assembly of dMSC-460.[38] The 0D MSCs (as labelled dMSCs) are suggested to self-assemble into 2D NPLs readily at relatively low temperatures.[36-42] The 0D MSCs and the assembled 2D NPLs display similar static optical absorption with a sharp doublet.[36-38,41,42]

(36) Liu, Y.; Willis, M.; Rowell, N.; Luo, W.; Fan, H.; Han, S.; Yu, K. Effect of small molecule additives in the prenucleation stage of semiconductor CdSe quantum dots. *J. Phys. Chem. Lett*. **2018**, *9*, 6356−6363.

(37) Zhu, J.; Cao, Z.; Zhu, Y.; Rowell, N.; Li, Y.; Wang, S.; Zhang, C.; Jiang, G.; Zhang, M.; Zeng, J.; Yu, K. Transformation pathway from CdSe magic-size clusters with absorption doublets at 373/393 nm to clusters at 434/460 nm. *Angew. Chem. Int. Ed*. **2021**, *60*, 20358–20365.

(38) Cao, Z.; Zhu, J.; Peng, J.; Meng, N.; Bian, F.; Luan, C.; Zhang, M.; Li, Y.; Yu, K.; Zeng, J. Transformation pathway from CdSe nanoplatelets with absorption doublets at 373/393 nm to nanoplatelets at 434/460 nm. *J. Phys. Chem. Lett*. **2022**, *13*, 3983−3989.

(39) Ouyang, J.; Zaman, M. B.; Yan, F. J.; Johnston, D.; Li, G.; Wu, X.; Leek, D.; Ratcliffe, C. I.; Ripmeester, J. A.; Yu, K. Multiple families of magic-sized CdSe nanocrystals with strong bandgap photoluminescence via noninjection one-pot syntheses. *J. Phys. Chem. C* **2008**, *112*, 13805−13811.

(40) Yu, K.; Ouyang, J.; Zaman, M. B.; Johnston, D.; Yan, F. J.; Li, G.; Ratcliffe, C. I.; Leek, D. M.; Wu, X.; Stupak, J.; Jakubek, Z.; Whitfield, D. Single-sized CdSe nanocrystals with bandgap photoemission via non-injection one-pot approach. *J. Phys. Chem. C* **2009**, *113*, 3390−3401.

(41) Liu, Y.; Zhang, B.; Fan, H. S.; Rowell, N.; Willis, M.; Zheng, X. T.; Che, R. C.; Han, S.; Yu, K. Colloidal CdSe 0-dimension nanocrystals and their self-assembled 2-dimension structures. *Chem. Mater*. **2018**, *30*, 1575−1584.

(42) Liu, Y.; Rowell, N.; Willis, M.; Zhang, M.; Wang, S.; Fan, H.; Huang, W.; Chen, X.; Yu, K. Photoluminescent colloidal nanohelices self-assembled from CdSe magic-size clusters via nanoplatelets. *J. Phys. Chem. Lett*. **2019**, *10*, 2794−2801.

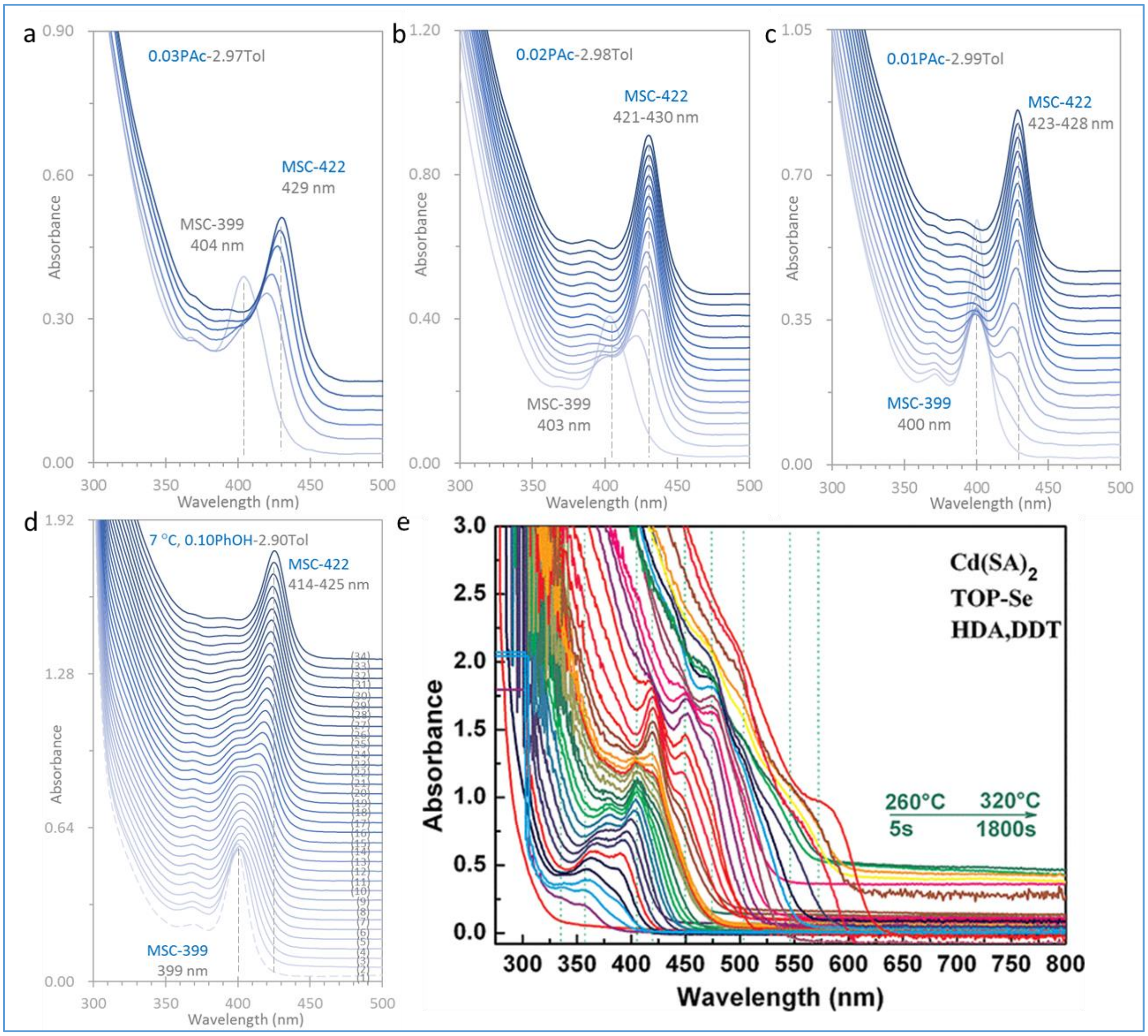

Figure S10-4. Temporal evolution of optical absorption spectra with and without offset. Parts a to e are respectively those for Figures 1d, 1e, 1f, and 5a, but with offset. Part e,[44] the absorption peak redshifts from 330 to 475 nm (without and with offset), when the reaction temperature increases from 260 to 320 °C. The redshift pattern is both uninterrupted and step-wise. The presentation with offset seems to obscure some of the essential information, regarding how the transformation proceeds. It is helpful to revisit previously-reported redshifts (especially those with offset), for a better understanding of how MSCs transform.[44,30] A thorough understanding of the pathway that underpins the isomerization should assist cluster engineering toward innovative applications such as in electronics. Eventually advanced single-

MSC switches may assist the development of sensitive and cooperative sensors, high data capacity storage in switchable memories, and efficient motors and shuttles required in nanoscale processes. Thus, more consideration should be paid to the redshift presentation (with and without offset) since this detail is an essential indicator regarding how the transformation occurs.

(30) Mule, A. S.; Mazzotti, S.; Rossinelli, A. A.; Aellen, M.; Prins, P. T.; van der Bok, J. C.; Solari, S. F.; Glauser, Y. M.; Kumar, P. V.; Riedinger, A.; Norris, D. J. Unraveling the growth mechanism of magic-sized semiconductor nanocrystals. *J. Am. Chem. Soc*. **2021**, *143*, 2037–2048.
(44) Sun, M.; Yang, X. Phosphine-free synthesis of high-quality CdSe nanocrystals in noncoordination solvents: “activating agent” and “nucleating agent” controlled nucleation and growth. *J. Phys. Chem. C* **2009**, *113*, 8701–8709.

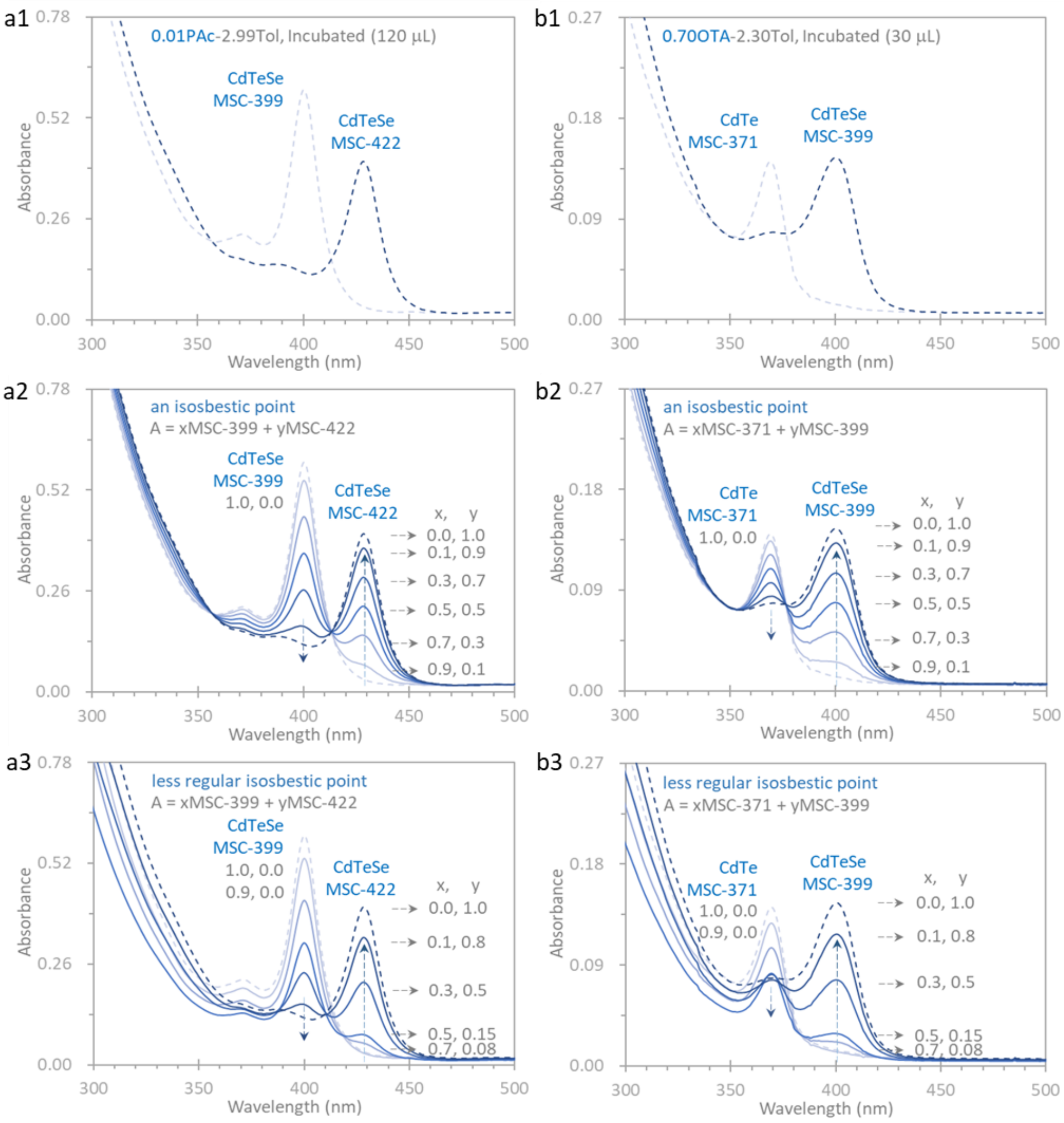

Figure S11. Simulation of the presence of an isosbestic point in a PC-assisted transformation. The isomerization is shown from CdTeSe MSC-399 to CdTeSe MSC-422 in the left panel. The transformation from CdTe MSC-371 to CdTeSe MSC-399 is in the right panel. The isomerization and transformation is PC-assisted, and has the three key steps illustrated in Scheme 1 and Figure S1.2f and Figure S3-1. In Parts a1 and b1 (top panel), the dashed traces are experimental optical absorption spectra. Based on them, a spectral simulation is performed. The simulated spectra show isosbestic points that are either regular (Parts a2 and b2, middle panel) or distorted (Parts a3 and b3, bottom panel).

The two spectra in Part a1 are those for 0 min (CdTeSe MSC-399) and 45 min (CdTeSe MSC-422) in Figure 1f; the incubated sample (120 μL) is dispersed in the mixture of Tol (2.99 mL) and PAc (0.01 mL). The two spectra in Part b1 are those for 0 min (CdTe MSC-371) and 90 min (CdTeSe MSC-399) in Figure 1b of Reference 28 (in the main text); an incubated sample (30 μL) is dispersed in Tol (2.3 mL); when CdTeSe MSC-399 disappears completely, OTA (0.70 mL) is added.

When an isosbestic point is detected, a direct transformation from A to B occurs.[45] Also, an indirect transformation may happen which is PC-assisted. For the latter case of the MSC-1 to MSC-2 transformation (that via MSC-1 to PC-1, then to PC-2, and to MSC-2), the simulation (Parts a2, a3, b2, and b3) is performed with the molar fraction of x for MSC-1 (reactant) and of y for MSC-2 (product). Each simulated spectrum in Parts a2 and a3 is obtained with Equation (1), while Equation (2) is used for each in Parts b2 and b3.

$$A = x*MSC\text{-}399 + y*MSC\text{-}422 \qquad (1)$$

$$A = x*MSC\text{-}371 + y*MSC\text{-}399 \qquad (2)$$

In Parts a2 and b2 (middle panel), we assume

$$x + y = 1 \qquad (3)$$

In this case, the PC-1 to PC-2 step is rate-determining, and a regular isosbestic point is obtained. The transformation appears to be “direct”.

In Parts a3 and b3 (bottom panel), we assume

$$x + y + z = 1 \qquad (4)$$

where z is the molar fraction of the PC-2; it is easy to understand that z represents the amount of mass missing from the MSCs during the course of the transformation. Under such a case, the PC-2 to MSC-2 step is rate-determining. During the transformation, the values of $z = 1 – (x + y)$ takes on 0, 0.10, 0.22, 0.35, 0.65, 0.10, and 0, as indicated. A distorted isosbestic point is obtained.

Let us testify further the result from our simulation that a regular or distorted isosbestic point is

obtained when Step 2b or Step 2c is rate-determining during the PC-assisted transformation (Pathway 2). At the wavelength λ of the isosbestic point (Parts a1 and b1), MSC-1 and MSC-2 have the same strength (optical density) as shown by Equation (5).

$$A_{1\lambda} = A_{2\lambda} \quad (5)$$

For MSC-1 and MSC-2, we then define the strength ratios at the wavelengths of the isosbestic point and the peaks of a_1 and a_2, respectively,

$$a_1 = A_{1\lambda}/A_{1peak} \quad (6)$$

$$a_2 = A_{2\lambda}/A_{2peak} \quad (7)$$

$$a_1 * A_{1peak} = a_2 * A_{2peak} \quad (8)$$

When MSC-1 transforms to MSC-2 at time t, they both contribute to the strength at the wavelength λ of the isosbestic point:

$$A_{\lambda t} = A_{1\lambda t} + A_{2\lambda t} \quad (9)$$

$$A_{1\lambda t} = x * a_1 * A_{1peak} \quad (10)$$

$$A_{2\lambda t} = y * a_2 * A_{2peak} \quad (11)$$

For Parts a2 and b2 with x + y = 1,

$$A_{\lambda t} = x * a_1 * A_{1peak} + y * a_2 * A_{2peak}$$

$$= (x + y) * a_1 * A_{1peak} = a_1 * A_{1peak} \quad (12)$$

$A_{\lambda t}$ is constant during the transformation, suggesting that the isosbestic point is regular.

For Parts a3 and b3 with x + y + z = 1,

$$A_{\lambda t} = x * a_1 * A_{1peak} + y * a_2 * A_{2peak}$$

$$= (x + y) * a_1 * A_{1peak}$$

$$= (1 - z) a_1 * A_{1peak} \quad (13)$$

$A_{\lambda t}$ varies with the molar fraction of the PC during a transformation; thus, the "isosbestic point" is distorted. This distortion suggests a measure of the amount of the otherwise invisible PC that is present during a transformation.

In a side note, Pathway 1 and Pathway 2 may occur in a dispersion, and the "isosbestic point" can be also distorted. Here, we only discuss the case when the PC-assisted pathway, Pathway 2, dominates; the isosbestic point can be either regular or distorted.

(45) Endo, M.; Fukui, T.; Jung, S.; Yagai, S.; Takeuchi, M.; Sugiyasu, K. Photoregulated living supramolecular polymerization established by combining energy landscapes of photoisomerization and nucleation-elongation processes. *J. Am. Chem. Soc*. **2016**, *138*, 14347–14353.

REFERENCE:

(1) Zhu, Y.; Wei, D.; Zhang, W.; Tang, M. New theoretical insights into several common hydrogen migration reactions. *Univ. Chem.* **2014***, 29*, 52–57.

(2) Wilson, E. Isomerization of hydrocarbons. *Chem. Rev.* **1937**, *21*, 129−167.

(3) Bergmann, E. Isomerization of unsaturated hydrocarbons. *Chem. Rev.* **1941**, *29*, 529−551.

(4) Wyman, G. M. The cis-trans isomerization of conjugated compounds. *Chem. Rev*. **1955**, *55*, 625–657.

(5) Zhang, B.; Zhu, T.; Ou, M.; Rowell, N.; Fan, H.; Han, J.; Tan, L.; Dove, M. T.; Ren, Y.; Zuo, X.; Han, S.; Zeng, J.; Yu, K. Thermally-induced reversible structural isomerization in colloidal semiconductor CdS magic-size clusters. *Nat. Commun*. **2018**, *9*, 2499.

(6) Zhu, D.; Hui, J.; Rowell, N.; Liu, Y.; Chen, Q. Y.; Steegemans, T.; Fan, H.; Zhang, M.; Yu, K. Interpreting the ultraviolet absorption in the spectrum of 415 nm-bandgap CdSe magic-size clusters. *J. Phys. Chem. Lett.* **2018**, *9*, 2818−2824.

(7) Luan, C.; Gokcinar Ö. Ö.; Rowell, N; Kreouzis, T.; Han, S.; Zhang, M.; Fan, H.; Yu, K. Evolution of two types of CdTe magic-size clusters from a single induction period sample. *J. Phys. Chem. Lett.* **2018**, *9*, 5288–5295.

(8) Yang, Y.; Li, Y.; Luan, C.; Rowell, N.; Wang, S.; Zhang, C.; Huang, W.; Chen, X.; Yu, K. Transformation pathways in colloidal CdTeSe magic-size clusters. *Angew. Chem. Int. Ed*. **2022**, *61*, e202114551.

(9) He, L.; Luan, C.; Rowell, N.; Zhang, M.; Chen, X.; Yu, K. Transformations among colloidal semiconductor magic-size clusters. *Acc. Chem. Res.* **2021**, *54*, 776−786.

(10) Williamson, C. B.; Nevers, D. R.; Nelson, A.; Hadar, I.; Banin, U.; Hanrath, T.; Robinson, R. D. Chemically reversible isomerization of inorganic clusters. *Science* **2019**, *363*, 731−735.

(11) Liu, M.; Wang, K.; Wang, L.; Han, S.; Fan, H.; Rowell, N.; Ripmeester, J. A.; Renoud, R.; Bian, F.; Zeng, J.; Yu, K. Probing intermediates of the induction period prior to nucleation and growth of semiconductor quantum dots. *Nat. Commun*. **2017**, *8*, 15467.

(12) Zhu, T.; Zhang, B.; Zhang, J.; Lu, J.; Fan, H.; Rowell, N.; Ripmeester, J. A.; Han, S.; Yu, K. Two-step nucleation of CdS magic-size nanocluster MSC-311. *Chem. Mater*. **2017**, *29*, 5727−5735.

(13) Wang, L.; Hui, J.; Tang, J.; Rowell, N.; Zhang, B.; Zhu, T.; Zhang, M.; Hao, X.; Fan, H.; Zeng, J.; Han, S.; Yu, K. Precursor self-assembly identified as a general pathway for colloidal semiconductor magic-size clusters. *Adv. Sci*. **2018**, *5*, 1800632.

(14) Rubin, V. C. Galaxy dynamics and the mass density of the universe, *Proc. Natl. Acad. Sci.* **1993**, *90*, 4814–4821.

(15) Luan, C.; Shen, Q.; Rowell, N.; Zhang, M.; Chen, X.; Huang, W.; Yu, K. A real-time in-situ demonstration of direct and indirect transformation pathways in CdTe magic-size clusters at room temperature. *Angew. Chem. Int. Ed.* **2022**, *61*, e202205784.

(16) Luan, C.; Tang, J.; Rowell, N.; Zhang, M.; Huang, W.; Fan, H.; Yu, K. Four types of CdTe magic-size clusters from one prenucleation stage sample at room temperature. *J. Phys. Chem. Lett*. **2019**, *10*, 4345−4353.

(17) Shen, Q.; Luan, C.; Rowell, N.; Zhang, M.; Wang, K.; Willis, M.; Chen, X.; Yu, K. Reversible transformations at room temperature among three types of CdTe magic-size clusters. *Inorg. Chem*. **2021**, *60*, 4243−4251.

(18) Gao, D.; Hao, X.; Rowell, N.; Kreouzis, T.; Lockwood, D. J.; Han, S.; Fan, H.; Zhang, H.; Zhang, C.; Jiang, Y.; Zeng, J.; Zhang, M.; Yu, K. Formation of colloidal alloy semiconductor CdTeSe magic-size clusters at room temperature. *Nat. Commun*. **2019**, *10*, 1674.
(19) Zhang, H.; Luan, C.; Gao, D.; Zhang, M.; Rowell, N.; Willis, M.; Chen, M.; Zeng, J.; Fan, H.; Huang, W.; Chen, X.; Yu, K. A room-temperature formation pathway for CdTeSe alloy magic-size clusters. *Angew. Chem. Int. Ed*. **2020**, *59*, 16943−16952.
(20) Shubina, E. S.; Belkova, N. V.; Krylov, A. N.; Vorontsov, E. V.; Epstein, L. M.; Gusev, D. G.; Niedermann, M.; Berke, H. S. Evidence for intermolecular M-H···H-OR hydrogen bonding: interaction of $WH(CO)_2(NO)L_2$ hydrides with acidic alcohols. *J. Am. Chem. Soc.* **1996**, *118*, 1105-1112.
(21) Shim, D.; Lee, J.; Kang, J. Multiscale isomerization of magic-sized inorganic clusters chemically driven by atomic-bond exchanges. *Chem. Mater.* **2022**, 34, 9527–9535.
(22) Fojtik, A.; Weller, H.; Koch, U.; Henglein, A. Photo-physics of extremely small CdS particles: Q-state CdS and magic agglomeration numbers. *Ber. Bunsenges. Phys. Chem.* **1984**, *88*, 969−977.
(23) Nevers, D. R.; Williamson, C. B.; Savitzky, B. H.; Hadar, I.; Banin, U.; Kourkoutis, L. F.; Hanrath, T.; Robinson, R. D. Mesophase formation stabilizes high-purity magic-sized clusters. *J. Am. Chem. Soc.* **2018**, *140*, 3652−3662.
(24) Han, H.; Kallakuri, S.; Yao, Y.; Williamson, C. B.; Nevers, D. R.; Savitzky, B. H.; Skye, R. S.; Xu, M.; Voznyy, O.; Dshemuchadse, J.; Kourkoutis, L. F.; Weinstein, S. J.; Hanrath, T.; Robinson, R. D. Multiscale hierarchical structures from a nanocluster mesophase. *Nat. Mater*. **2022**, *21*, 518−525.
(25) He, L.; Luan, C.; Liu, S.; Chen, M.; Rowell N.; Wang; Z.; Li, Y.; Zhang, C.; Lu, J.; Zhang, M.; Liang, B.; Yu, k.; Transformations of magic-size clusters via precursor compound cation exchange at room temperature. *J. Am. Chem. Soc*. **2022**, *144*, 19060−19069.
(26) Ptatschek, V.; Schmidt, T.; Lerch, M.; Miiller, G.; Spanhel, L.; Emmerling, A.; Fncke, J.; Foitzik, A. H.; Langer, E. Quantized aggregation phenomena in II−VI-semiconductor colloids. *Ber. Bunsenges. Phys. Chem*. **1998**, *102*, 85−95.
(27) Kasuya, A.; Sivamohan, R.; Barnakov, Y. A.; Dmitruk, I. M.; Nirasawa, T.; Romanyuk, V. R.; Kumar, V.; Mamykin, S. V.; Tohji, K.; Jeyadevan, B.; Shinoda, K.; Kudo, T.; Terasaki, O.; Liu, Z.; Belosludov, R. V.; Sundararajan, V.; Kawazoe, Y. Ultra-stable nanoparticles of CdSe revealed from mass spectrometry. *Nat. Mater*. **2004**, *3*, 99−102.
(28) Beecher, A. N.; Yang, X.; Palmer, J. H.; LaGrassa, A. L.; Juhas, P.; Billinge, S. J.; Owen, J. S. Atomic structures and gram scale synthesis of three tetrahedral quantum dots. *J. Am. Chem. Soc.* **2014**, *136*, 10645−10653.
(29) Hsieh, T.; Yang, T.; Hsieh, C.; Huang, S.; Yeh, Y.; Chen, C.; Li, E.; Liu, Y. Unraveling the structure of magic-size $(CdSe)_{13}$ cluster pairs. *Chem. Mater.* **2018**, *30*, 5468−5477.
(30) Mule, A. S.; Mazzotti, S.; Rossinelli, A. A.; Aellen, M.; Prins, P. T.; Bok, J. C. V. D.; Solari, S. F.; Glauser, Y. M.; Kumar, P. V.; Riedinger, A.; Norris, D. J. Unraveling the growth mechanism of magic-sized semiconductor nanocrystals. *J. Am. Chem. Soc.* **2021**, *143*, 2037−2048.
(31) Riedinger, A.; Ott, F. D.; Mule, A.; Mazzotti, S.; Knusel, P. N.; Kress, S. J. P.; Prins, F.; Erwin, S. C.; Norris, D. J. An intrinsic growth instability in isotropic materials leads to quasi-two-dimensional nanoplatelets. *Nat. Mater*. **2017**, *16*, 743–748.

(32) Ithurria, S.; Dubertret, B. Quasi 2D colloidal CdSe platelets with thicknesses controlled at the atomic level. *J. Am. Chem. Soc.* **2008**, *130*, 16504–16505.
(33) Ithurria, S.; Bousquet, G.; Dubertret, B. Continuous transition from 3D to 1D confinement observed during the formation of CdSe nanoplatelets. *J. Am. Chem. Soc*. **2011**, *133*, 3070–3077.
(34) Chen, Y.; Chen, D.; Li, Z.; Peng, X. Symmetry-breaking for formation of rectangular CdSe two-dimensional nanocrystals in zinc-blende structure. *J. Am. Chem. Soc.* **2017**, *139*, 10009–10019.
(35) Moghaddam, N.; Dabard, C.; Dufour, M.; Po, H.; Xu, X.; Pons, T.; Lhuillier, E.; Ithurria, S. Surface modification of CdE (E: S, Se, and Te) nanoplatelets to reach thicker nanoplatelets and homostructures with confinement-induced intraparticle type I energy level alignment. *J. Am. Chem. Soc*. **2021**, *143*, 1863–1872.
(36) Liu, Y.; Willis, M.; Rowell, N.; Luo, W.; Fan, H.; Han, S.; Yu, K. Effect of small molecule additives in the prenucleation stage of semiconductor CdSe quantum dots. *J. Phys. Chem. Lett*. **2018**, *9*, 6356−6363.
(37) Zhu, J.; Cao, Z.; Zhu, Y.; Rowell, N.; Li, Y.; Wang, S.; Zhang, C.; Jiang, G.; Zhang, M.; Zeng, J.; Yu, K. Transformation pathway from CdSe magic-size clusters with absorption doublets at 373/393 nm to clusters at 434/460 nm. *Angew. Chem. Int. Ed*. **2021**, *60*, 20358–20365.
(38) Cao, Z.; Zhu, J.; Peng, J.; Meng, N.; Bian, F.; Luan, C.; Zhang, M.; Li, Y.; Yu, K.; Zeng, J. Transformation pathway from CdSe nanoplatelets with absorption doublets at 373/393 nm to nanoplatelets at 434/460 nm. *J. Phys. Chem. Lett*. **2022**, *13*, 3983−3989.
(39) Ouyang, J.; Zaman, M. B.; Yan, F. J.; Johnston, D.; Li, G.; Wu, X.; Leek, D.; Ratcliffe, C. I.; Ripmeester, J. A.; Yu, K. Multiple families of magic-sized CdSe nanocrystals with strong bandgap photoluminescence via noninjection one-pot syntheses. *J. Phys. Chem. C* **2008**, *112*, 13805−13811.
(40) Yu, K.; Ouyang, J.; Zaman, M. B.; Johnston, D.; Yan, F. J.; Li, G.; Ratcliffe, C. I.; Leek, D. M.; Wu, X.; Stupak, J.; Jakubek, Z.; Whitfield, D. Single-sized CdSe nanocrystals with bandgap photoemission via non-injection one-pot approach. *J. Phys. Chem. C* **2009**, *113*, 3390−3401.
(41) Liu, Y.; Zhang, B.; Fan, H. S.; Rowell, N.; Willis, M.; Zheng, X. T.; Che, R. C.; Han, S.; Yu, K. Colloidal CdSe 0-dimension nanocrystals and their self-assembled 2-dimension structures. *Chem. Mater*. **2018**, *30*, 1575−1584.
(42) Liu, Y.; Rowell, N.; Willis, M.; Zhang, M.; Wang, S.; Fan, H.; Huang, W.; Chen, X.; Yu, K. Photoluminescent colloidal nanohelices self-assembled from CdSe magic-size clusters via nanoplatelets. *J. Phys. Chem. Lett*. **2019**, *10*, 2794−2801.
(43) Busatto, S.; Spallacci, C.; Meeldijk, J. D.; Howes, S.; Donega, C. de M. Room-temperature interconversion between ultrathin CdTe magic-size nanowires induced by ligand shell dynamics. *J. Phys. Chem. C* **2022**, *126*, 15280–15297.
(44) Sun, M.; Yang, X. Phosphine-free synthesis of high-quality CdSe nanocrystals in noncoordination solvents: “activating agent” and “nucleating agent” controlled nucleation and growth. *J. Phys. Chem. C* **2009**, *113*, 8701–8709.
(45) Endo, M.; Fukui, T.; Jung, S.; Yagai, S.; Takeuchi, M.; Sugiyasu, K. Photoregulated living supramolecular polymerization established by combining energy landscapes of photoisomerization and nucleation-elongation processes. *J. Am. Chem. Soc*. **2016**, *138*,

14347–14353.
